# Artificial intelligence: the human response to approach the complexity of big data in biology

**DOI:** 10.1093/gigascience/giaf057

**Published:** 2025-06-12

**Authors:** Giovanni Melandri, Georges R-Radohery, Chloé Beaumont, Sara M de Cripan, Coralie Muller, Luca Piras, Maria Alcina Pereira, Andreia Ferreira Salvador, Xavier Domingo-Almenara, Marie Bolger, Sophie Colombié, Sylvain Prigent, Biotza Gutierrez Arechederra, Nuria Canela Canela, Pierre Pétriacq

**Affiliations:** University of Bordeaux, INRAE, UMR 1332 BFP, Villenave d’Ornon 33140, France; School of Plant Sciences, University of Arizona, Tucson, AZ 85721, USA; University of Bordeaux, INRAE, UMR 1332 BFP, Villenave d’Ornon 33140, France; University of Bordeaux, INRAE, UMR 1332 BFP, Villenave d’Ornon 33140, France; Centre for Omics Sciences (COS), Eurecat—Technology Centre of Catalonia & Rovira i Virgili University Joint Unit, Unique Scientific and Technical Infrastructures (ICTS), Reus, Catalonia 43204, Spain; University of Bordeaux, INRAE, UMR 1332 BFP, Villenave d’Ornon 33140, France; Inria, Univ. Bordeaux, INRAE, F-33400, Talence, France; EURECAT—Technology Centre of Catalonia, Barcelona, Catalonia 08005, Spain; Centre of Biological Engineering, University of Minho, 4704-553 Braga, Portugal; LABBELS—Associate Laboratory, Braga/Guimarães, Portugal; Centre of Biological Engineering, University of Minho, 4704-553 Braga, Portugal; LABBELS—Associate Laboratory, Braga/Guimarães, Portugal; Centre for Omics Sciences (COS), Eurecat—Technology Centre of Catalonia & Rovira i Virgili University Joint Unit, Unique Scientific and Technical Infrastructures (ICTS), Reus, Catalonia 43204, Spain; Department of Electrical, Electronic and Control Engineering (DEEEA), Universitat Rovira i Virgili, Tarragona, Catalonia 43007, Spain; Institute of Bio- and Geosciences, IBG-4: Bioinformatics, Forschungszentrum Jülich, CEPLAS, BioSC, Jülich 52429, Germany; University of Bordeaux, INRAE, UMR 1332 BFP, Villenave d’Ornon 33140, France; Bordeaux Metabolome, MetaboHUB, PHENOME-EMPHASIS, Villenave d’Ornon 33140, France; University of Bordeaux, INRAE, UMR 1332 BFP, Villenave d’Ornon 33140, France; Bordeaux Metabolome, MetaboHUB, PHENOME-EMPHASIS, Villenave d’Ornon 33140, France; EURECAT—Technology Centre of Catalonia, Barcelona, Catalonia 08005, Spain; Centre for Omics Sciences (COS), Eurecat—Technology Centre of Catalonia & Rovira i Virgili University Joint Unit, Unique Scientific and Technical Infrastructures (ICTS), Reus, Catalonia 43204, Spain; University of Bordeaux, INRAE, UMR 1332 BFP, Villenave d’Ornon 33140, France; Bordeaux Metabolome, MetaboHUB, PHENOME-EMPHASIS, Villenave d’Ornon 33140, France

**Keywords:** artificial intelligence, machine learning, deep learning, omics, life science, biology

## Abstract

Since the late 2010s, artificial intelligence (AI), encompassing machine learning and propelled by deep learning, has transformed life science research. It has become a crucial tool for advancing the computational analysis of biological processes, the discovery of natural products, and the study of ecosystem dynamics. This review explores how the rapid increase in high-throughput omics data acquisition has driven the need for AI-based analysis in life sciences, with a particular focus on plant sciences, animal sciences, and microbiology. We highlight the role of omics-based predictive analytics in systems biology and innovative AI-based analytical approaches for gaining deeper insights into complex biological systems. Finally, we discuss the importance of FAIR (findable, accessible, interoperable, reusable) principles for omics data, as well as the future challenges and opportunities presented by the increasing use of AI in life sciences.

## Background

### The explosion of omics requires artificial intelligence in the study of life sciences

In the past 2 decades, research and society have entered the “big data” era of life sciences. Technological advances have enhanced our ability to measure qualitative and quantitative variations of internal biological molecules (e.g., DNA, RNA, proteins, metabolites) and phenotypes, making the acquisition of large and complex omics datasets within a single experiment increasingly common.

The explosion of omics data in life sciences began with genomics, which was driven by the emergence of DNA next-generation sequencing (NGS) platforms nearly 20 years ago. While the groundbreaking discovery of the Sanger DNA sequencing method dates back to the 1970s, it took 3 decades for the advent of second-generation short-read sequencing-based NGS to further revolutionize DNA sequencing, dramatically increasing its affordability and throughput. This has led to the *de novo* assembly of thousands of animal and plant genomes [1, 2] and to the discovery of millions of genome-wide single nucleotide polymorphic (SNP) variants. High-throughput analysis of multiple gene transcripts (i.e., transcriptomics) began in the mid-1990s with the introduction of hybridization-based microarray technologies. However, it was not until the 2000s that NGS enabled a more accurate assessment of the qualitative and quantitative diversity (e.g., large dynamic range of expression levels and alternative splicing variants) of messenger RNAs. This technique, known as RNA sequencing (RNA-seq), uses NGS to sequence complementary DNAs (cDNAs) derived from RNA transcripts [3, 4]. The current third-generation single-molecule sequencing technologies (e.g., PacBio and Oxford Nanopore Technologies) have further improved the read length, throughput, and accuracy of data collection in the field of genomics and transcriptomics [5, 6]. The field of proteomics and metabolomics relies on the use of mass spectrometry (MS) techniques to explore the diversity of proteins and metabolites in both a qualitative and quantitative manner. Although mass spectrometers have been available since the late 1940s, it was their integration with gas chromatography (GC) or liquid chromatography (LC) and the development of ionization techniques such as electrospray ionization (ESI) and matrix-assisted laser desorption ionisation (MALDI) in the late 1980s that truly expanded their application to biological research [7, 8]. There are various ionization techniques in mass spectrometry and electronic impact ionization that, while historically important for profiling primary compounds of biological samples, have been largely superseded by softer ionization methods such as ESI and MALDI. These newer techniques are more suitable for analyzing biomolecules as they cause less fragmentation and tend to preserve the integrity of molecules during ionization. Over the past 20 years, the advancement of high-resolution (HR) MS has been crucial in significantly enhancing the identification of proteins and metabolites. This has driven the widespread application of proteomics and metabolomics in the analysis of complex biological samples [9, 10].

Recent advancements in imaging technologies have improved life science research, benefiting not only the medical field [11] but also plant sciences. The field of plant/crop phenomics has rapidly evolved due to breakthroughs in sensor technology, machine vision, and automation technology [12]. Today, automated, noninvasive, high-throughput imaging and sensor technologies generate vast amounts of image and sensor data, presenting both opportunities and challenges for analysis.

**Figure 1:**
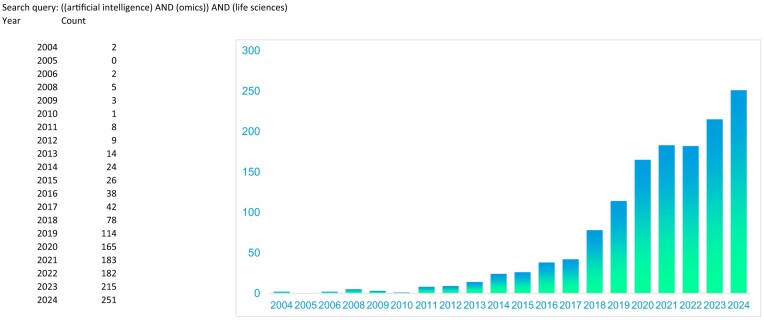
Number of publications found in PubMed including [artificial intelligence] AND [omics] AND [life sciences] from 2004 to 2024. In total, 1,362 publications were found (19 September 2024). Considering the past 20 years, a literature search using the queries [omics] AND [artificial intelligence] AND [life sciences] confirms that AI in life sciences is a rapidly expanding field of research.

The ability to generate high-throughput large-scale omics data through advanced technologies offers an unprecedented opportunity for exploring the complexity of biological systems in depth. Furthermore, integrating multiple omics datasets from a single experiment facilitates a “holistic” approach, revealing the potential of how the “molecular endophenome” (at the cellular/tissue level) is regulated and connected with the “external phenome” of biological organisms. However, disentangling and deciphering the intricate relationships among tens of thousands (sometimes millions) of molecular variables (i.e., SNPs, transcripts, proteins, and metabolites), which are interconnected among themselves and with the final phenotype, has been a major challenge in biological research over the past 2 decades [13, 14]. The use of high-dimensional solutions on complex omics datasets to address fundamental biological questions exceeds the capacity of the human brain. This requires a computer-based analytical approach, which can benefit from the constant improvements in machine processing power at all levels (single machine or physical/cloud-based clusters). For these reasons, “artificial intelligence” (AI) has emerged as a key tool in life science research (Fig. 1), with the expectation that AI will lead or assist in most of the future biological discoveries.

### Artificial intelligence, machine learning, and deep learning

Despite its widespread use, the term AI remains an elusive “buzzword.” From a scientific perspective, the difficulty in defining AI is associated with the complexity of the concept of intelligence *per se* and with the fact that, despite a resurgence of interest in AI started in the 1990s, fast progresses in AI research rapidly developed only from the 2010s, and, thus, this field of research is far from reaching a level of maturity that can be translated into a clear definition [15].

**Figure 2:**
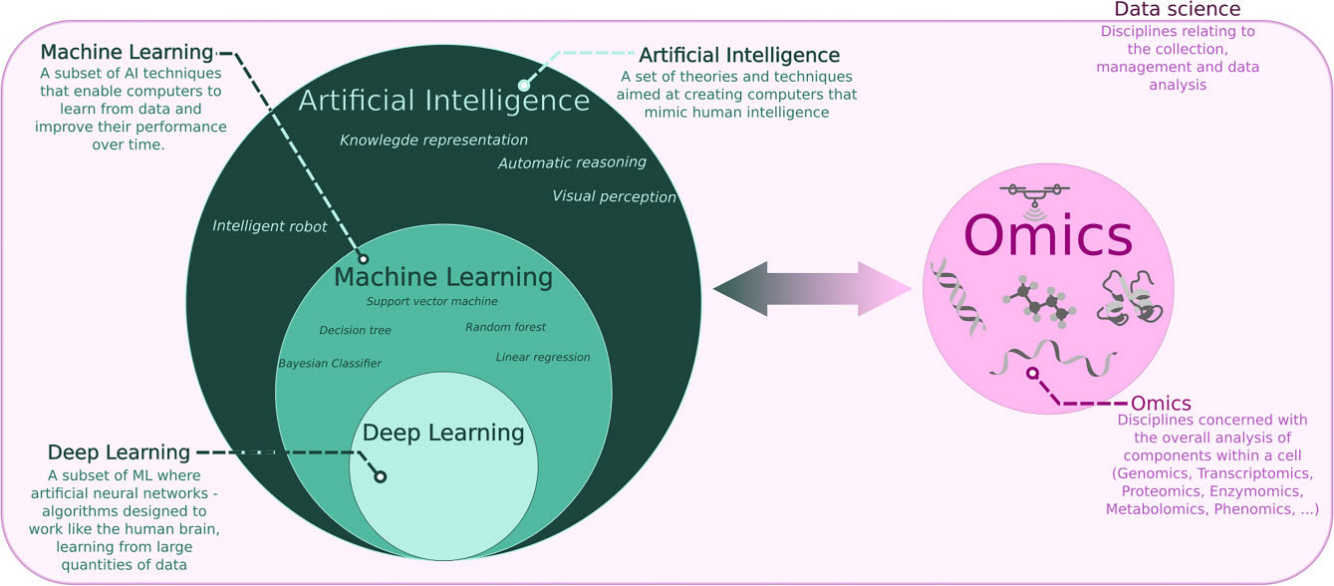
Data science in the era of artificial intelligence, machine learning and deep learning: a dynamic schematic breakdown.

Oversimplifying, AI can be considered a branch of computer science focused on programming machines (typically 1 or more computers) to perform a specific tasks by learning from the information present in specific dataset(s) [16] (Fig. 2). This definition is appropriate only for “artificial narrow intelligence” or “weak AI,” which is currently used for many routine and nearly ubiquitous applications such as spam filtering, speech recognition, language translation, online advertising, image tagging, and so on. However, this definition is not accurate for “artificial general intelligence” or “artificial super intelligence,” which are both still far from being achieved. These forms of AI aim to develop machines capable of learning and understand from data in ways that are comparable to or surpass human intelligence [17].

**Figure 3:**
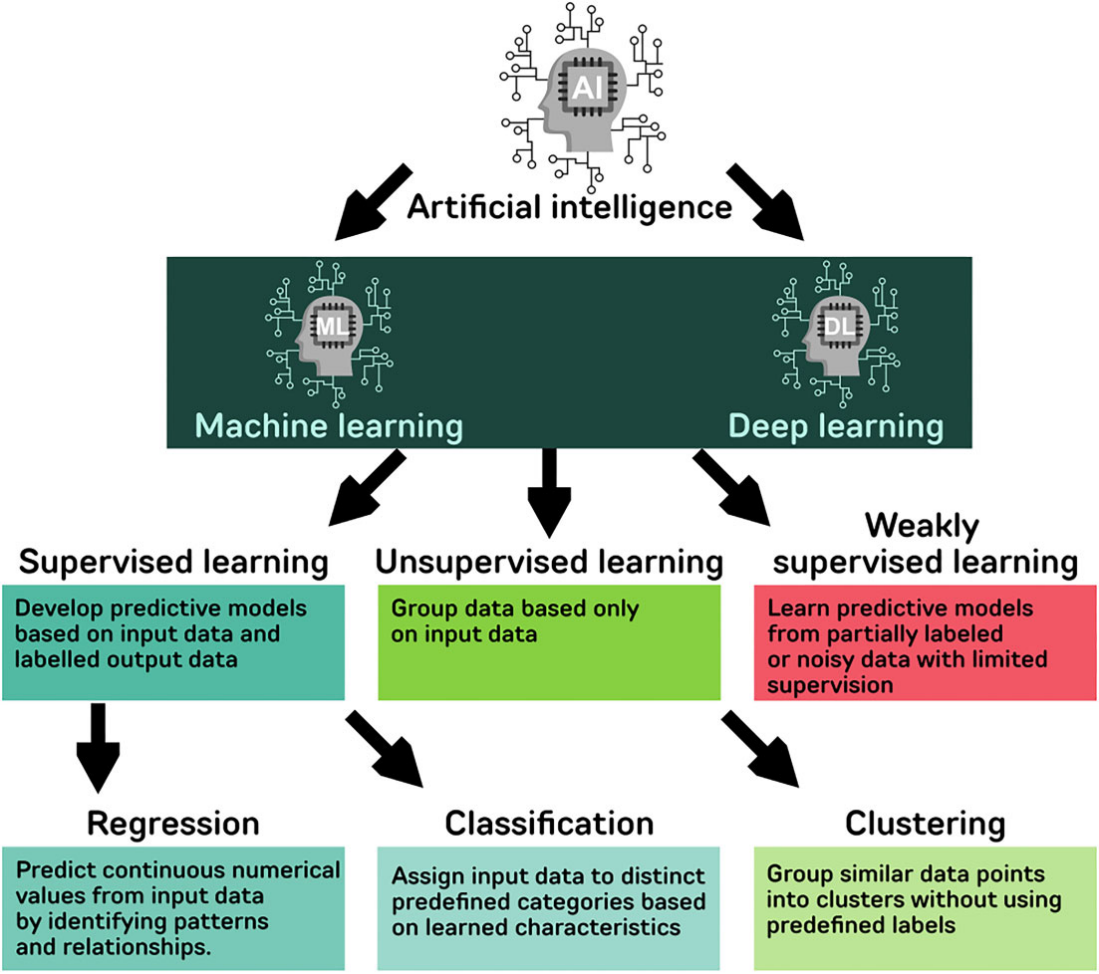
Major approaches in machine learning and deep learning.

Considering “artificial narrow intelligence” (hereafter AI will refer to this term) and, particularly, its most popular subfield “machine learning” (ML), the “learning” feature defines the process of using an algorithm that finds complex patterns in the training data and translates them into an object-level algorithm (such as a model of a domain problem), which, in turn, is able to make predictions about unobserved data. It is in the context of ML that biological research has benefited the most from the use of large and complex omics data [18, 19]. Biological data-based ML models have the double target of (i) accurately predicting experimental data and (ii) using this predicting ability to inform and direct the efforts of future research. When developing ML models, the characteristics of the training data determine the learning approach. Training data refer to the dataset used to teach an ML model, and a key distinction is whether these training data include annotations, which determine the learning method applied. Training data can be labeled or unlabeled. Labeled data contain explicit tags, such as categories or numerical values, allowing the model to learn from predefined outcomes. When the data are labeled, the model follows a supervised learning approach. In contrast, unlabeled data lack predefined tags, requiring the model to extract patterns and relationships independently—a process known as unsupervised learning (Fig. 3) [20]. On the contrary, if the same data are labeled (with qualitative or quantitative tags), the ML model is defined as based on “supervised” learning. Unsupervised ML models are mainly used to deal with clustering problems where the algorithms (e.g., K-means clustering or DBSCAN clustering) find relationships in the overall structure of the training data [20]. In supervised learning, the algorithm uses the provided labels as a guide to map data points to specific outcomes or classifications.

Machine learning is built on a few fundamental algorithms that serve as the foundation for more advanced techniques [21]. Here, we focus on a nonexhaustive list of these algorithms, particularly those that are interpretable and can clarify the importance of each variable in making predictions. This interpretability is especially valuable in life sciences, where it allows for a thorough utilization of information found in omics data—such as genomics, proteomics, and metabolomics—to uncover biological insights [22].

First, linear regression, used in supervised learning, predicts a continuous target variable by establishing a linear relationship between inputs and outputs and adjusting parameters to minimize the difference between expected and actual values. Linear regression is highly interpretable, as it establishes a clear linear relationship between input features and the target variable, allowing a straightforward understanding of how changes in each input affect the predicted outcome. The training process involves iterative adjustments to reduce prediction errors, often guided by optimization techniques [23]. Linear regression forms the basis for methods like Ridge and Lasso regression, which incorporate penalties to mitigate overfitting, where the model performs well on the training data but poorly on new data, by constraining model complexity [24]. These extensions enhance robustness and inform the weight adjustment mechanisms central to neural networks, demonstrating its role as a building block in machine learning [25].

Next, support vector machines (SVMs) address classification by identifying an optimal boundary that maximizes the distance to the nearest data points, which are known as support vectors. The support vectors provide insight into which data points are most crucial for the classification boundary. By examining these vectors and their corresponding features, one can infer which aspects of the data are influential in decision-making. For datasets where linear separation is infeasible, SVMs employ kernel functions—such as polynomial or radial basis functions—to transform the data into a higher-dimensional space, enabling complex separations [26]. This emphasis on margin maximization and spatial transformation influences modern deep learning architectures, notably in convolutional neural networks, where kernel-based operations are prevalent [27].

Decision trees, another supervised learning approach, partition the feature space into distinct regions based on threshold values applied to input variables. Criteria that maximize class separation, such as reducing impurity (e.g., Gini index) or minimizing prediction variance for regression tasks, determine these splits. Their interpretability—from clear, rule-based decisions—makes them particularly appealing for applications requiring transparency, such as omics-driven research [28]. Moreover, integrating them into ensemble methods like random forests, where multiple trees vote to enhance accuracy, or gradient-boosted trees, which iteratively refine predictions, amplifies their utility. These ensembles illustrate how decision trees evolve into robust predictive tools [29].

We take as the last example naive Bayes, which offers a probabilistic framework for classification. It assumes that features are independent within each class and uses probabilities to determine the most likely class for a given set of data. By applying Bayes’s theorem, it calculates how likely something belongs to a specific category based on past data. Hence, a naive Bayes classifier provides probabilities for each class rather than hard classifications. Furthermore, each feature’s contribution to the final decision can be calculated based on its likelihood of occurrence on each class, thus giving a strong interpretability to the model. However, assumptions like data independence could be unrealistic for biological data. Naive Bayes forms the basis for more advanced probabilistic models, like Bayesian networks. A Bayesian network extends the naive Bayes classifier by allowing dependencies between variables, unlike naive Bayes, which assumes all features are conditionally independent given the class label. It represents a probabilistic graphical model where nodes (variables) have directed edges (dependencies) between them [30].

Since its formal introduction in 2006, deep learning (DL) [31], based on diverse artificial neural network (ANN) algorithms, has further boosted the use of ML in many fields of research, particularly in speech recognition and image analysis [32] but also in the biological field, such as in regulatory genomics and protein classification [33, 34] (Fig. 3). Advanced DL-based models represent the state-of-the-art of prediction accuracy in biological sciences [35, 36]. Nevertheless, they require the availability of very large-scale training data (with an associated high computational demand), and their interpretation remains elusive (they are often referred to as “black-box models”), with this elusiveness representing a limitation in biological experiments involving omics data for which identifying the most important predicting features and feature combinations is of primary importance [37]. Thus, when research is aimed at better understanding the functioning of biological systems, DL-based models are still difficult to be commonly applied [38, 39]. It is also for these reasons that in a society where AI algorithms are becoming more central than ever before in all aspects of our daily life, the concepts of “interpretable ML” and “explainable AI” are gaining an always increasing attention and importance [34, 40].

### Multiomics integration for ML analysis

As mentioned above, innovations in high-throughput acquisition of different omics data from single experiments are now enabling capturing different layers of biological complexity. In fact, application of omics approaches, such as transcriptomic, proteomics, and metabolomics [41], to large diversity panels and/or samples holds significant promise for unraveling the complexity of living systems. Despite their overall potential for discovery, the diverse nature of omics data acquired by different technological platforms requires the use of integration strategies to effectively harness their complementary information. Recent advances in multiomics analysis have been made possible by the development of various tools and methods that can resolve the heterogeneous nature of biological datasets, enabling their effective integration. Notably, consensus orthogonal partial least squares discriminant analysis (OPLS-DA) has emerged as an effective strategy for fusing multiomics data, combining multiple kernel learning with OPLS-DA [42]. The *mixOmics* R package provides a variety of multivariate methods for integrating omics datasets, including extensions of “Projection to Latent Structure” models for discriminant analysis and molecular signature identification [43]. Additionally, ML techniques, such as network-based diffusion and DL, are increasingly used to capture complex nonlinear associations in multiomics data [44]. Among the available R resources, packages such *moiraine* [45] provide a range of integrative methods for multiomics analyses, including sPLS and DIABLO from the *mixOmics* package [43], sO2PLS from the *OmicsPLS* package [46], and MOFA and MEFISTO from the *MOFA2* package [47].

### AI-based analysis of omics data in the fields of plant sciences, animal sciences, and microbial sciences

International initiatives are thriving in the field of AI-based analysis of omics data, aiming to advance the discovery of genotype–phenotype relationships. One such example is the *GLOMICAVE* project (GLobal OMIC data integration on Animal, Vegetal and Environment sectors), an international project that involves all the authors of this review paper. *GLOMICAVE* has created an innovative digital platform that connects genotype to phenotype through Big Data analytics and AI, using extensive public and experimental omic datasets [https://glomicave.eu/]. Likewise, cloud-based platforms like HiOmics offer a comprehensive analysis of biomedical large-scale omics data [48]. Such projects aim to facilitate the analysis of primary data and support large-scale omics experiments, thereby enhancing the utility of omics data on a massive scale and deepening our understanding of entire biological systems. In line with *GLOMICAVE*, and considering that the medical field has been extensively examined from an AI perspective, this review focuses on relevant applications from plant, animal, and microbial sciences.

#### Plant sciences and AI

The explosion of omics has radically transformed research in plant sciences, simultaneously driving the need for ML to handle datasets characterized by high complexity and dimensionality. A paradigmatic example is plant phenomics, which has rapidly shifted from a promising research sector with the potential of bridging the gap with genomic advances to becoming a widespread tool in plant and crop sciences [12]. This rapid progress was enabled by integrating advanced sensors and imaging technologies (e.g., RGB, multispectral, hyperspectral, thermal, and fluorescence cameras and sensors) with unmanned aerial vehicles (or drones) and ground robots, which are able to collect high-throughput phenotyping data. Approaches based on ML algorithms are now a practical and effective strategy for extracting traits and features from massive amounts of imaging- and sensor-based data. DL algorithms (e.g., convolutional neural networks [CNNs]) show the highest versatility and success in image-based plant phenotyping. These algorithms are particularly effective in predicting the effects of biotic and abiotic stresses [49, 50] and enabling rapid and accurate diagnostics of plant diseases [51]. Additionally, AI applications in root system architecture image analysis are emerging as crucial tools for improving this understudied field of research, which holds significant potential to boost a “Second Green Revolution” in agriculture [52]. Plant breeding is another branch of plant science that has been radically transformed by genomic advances, with breeders increasingly relying on genome-wide SNP marker-based genomic prediction (GP) to accelerate genetic gains for target traits in crops. Classic GP models are based on best linear unbiased prediction, but efforts to develop new ML-based and improved GP algorithms are ongoing [53]. Furthermore, different sources of nongenetic variability and nonadditive modes of gene action have made the choice and implementation of GP models challenging for improving complex plant traits, such as biomass and crop yield [54]. One possible solution to this problem is to incorporate other genome-to-phenome intermediate omics data (e.g., transcriptomics, proteomics, metabolomics) into the GP models to enhance their accuracy and predictive power [55]. The potential of ML models based on single intermediate omics, particularly metabolomics, the omics layer closer to the phenotype, has been demonstrated for the accurate prediction of crop yield, notably in maize [56] and rice [57, 58]. However, for plant breeding applications, the integration of large, highly dimensional, and “noisy” omics datasets for complex trait prediction remains a challenging field of study. This challenge will require the use of ML/DL techniques, leveraging their superior capability for Big Data analytics to effectively handle the complexity and scale of these datasets [59]. Interestingly, recent studies have highlighted innovative approaches in metabolomics-based ML prediction of plant complex traits showing innovative routes to identify breeding targets for plant improvement. For example, Colantonio et al. [60] identified candidate metabolites acting as fruit flavor enhancers and suppressors by metabolomics-based ML prediction of tomato and blueberry fruit flavor profiles. In efforts to improve plant tolerance to abiotic stress, Dussarrat et al. [61] applied a holistic ML prediction approach on environmental adaptations based on the multispecies metabolome of plants collected in the Atacama desert. This revealed a core set of metabolite targets for extreme climate resilience (sugars, stress-related amino acids, hormones, and antioxidants, including phenolics and major redox buffers).

#### Animal sciences and AI

Modern biotechnologies, bio-sensing hardware, and IT infrastructure have led to a high-throughput data collection era in livestock management, driving the need for faster and more efficient computational methods. While traditional information sources in animal breeding included phenotype and pedigree data, the field is increasingly incorporating genomic data such as SNPs, gene annotations, metabolic pathways, protein interaction networks, gene expression, and protein structure information. These data can enhance trait predictions and improve our understanding of the underlying biological phenotypes [62]. Despite these advancements in animal genetics, many challenges still persist. The widespread adoption of omics technologies is hindered by high cost and the need for expertise across diverse fields. Accurate recording of phenotypic data and population/sample size are other constraints that need to be addressed. However, omics technologies have shown their potential to identify superior and disease-resistant animals at an early stage [63]. For example, the metabolomes of healthy and unhealthy chickens were characterized and compared using untargeted mass spectrometry metabolomics [64]. Researchers were able to accurately distinguish chicken health status in multiple countries using a random forest (RF)–based ML model. This approach used raw mass spectrometry signals (unannotated *m/z* values) as input features, effectively overcoming one of the primary limitations of untargeted metabolomics: the need for metabolite annotation and identification. The use of ML models in animal breeding has recently attracted interest due to their exceptional flexibility and ability to capture patterns in large, noisy datasets [65]. For instance, gradient tree boosting (GTB) has proven to be an effective ML algorithm for predicting different breeding values. GTB-based models have identified a subset of genes contributing to feed efficiency in growing pigs using muscle transcriptome data [66]. The potential of combining metagenomics, metatranscriptomics, and metabolomics data was evaluated in rumen content, demonstrating their value as predictive markers for feed efficiency and their potential applications for selecting cows with high feed efficiency [67]. By using a RF-based model, they were able to predict feed efficiency using a preselected set of metabolites associated with this trait. Antimicrobial-resistant microorganisms pose significant challenges in livestock farming. A recent study [68] evaluated 10 supervised learning classifiers to predict *Escherichia coli* strains susceptible or resistant to 26 different antimicrobials using whole-genome shotgun sequencing in intensive poultry farming. This findings provided evidence of transmissible drug resistance in food-producing animals, which has contributed to the emergence of drug resistance in zoonotic pathogens.

#### Microbial sciences and AI

Microorganisms exist naturally in microbial communities and establish multiple interactions between each other and with their hosts. Omics experiments play a crucial role in studying these microorganisms in their natural environments, eliminating the need for their isolation and cultivation. However, interpreting omics information and integrating results from different studies remains challenging due to the complexity of omics data. AI has been increasingly applied to help interpret the variations found in microbial communities, particularly in the human microbiome and its relationship to health and disease [69–71]. In the field of environmental microbiology, recent reviews have highlighted major developments in the application of ML to microbial ecology omics [72]. This approach has been primarily applied to omics experiments using 16S rRNA gene sequencing data, which provide taxonomic information on microbial communities. RF-based ML architecture has been widely used due to its ease of implementation, interpretation, low cost, and the requirement of less data compared with DL [72]. Nevertheless, other ML algorithms, such as naive Bayes (NB), SVM, and KNN, have also been applied in the microbiology field [73]. In microbial ecology, the main objective of ML has been to predict the presence of certain microbes (e.g., microbial bioindicators, predicting environmental pollution, and key microbes affecting the performance of biotechnological processes), as well as to predict microbe-microbe and microbe–host interactions and facilitate data mining [72, 73]. For example, in the particular case of anaerobic digestion microbiology (a biotechnological process in which organic waste is converted to methane by microbial communities), there are several studies on AI applied to omics data. Three different algorithms (i.e., linear regression, SVM, and RF regression) were used to predict the production of medium-chain carboxylates, based on microbial community dynamics (16S rRNA) and the bioreactor’s productivity data. This study concluded that RF regression was the most effective algorithm for this task [74]. Similarly, another study compared 6 different ML algorithms—namely, GLMNET, RF, NNET, KNN, SVM, and extreme gradient boosting (XGBOOST)—to predict the performance of the anaerobic digestion process, using 16S rRNA genomics as the basis for the analysis [75]. Interactions between microorganisms are highly important and influence the activity of microbial communities. Syntrophic interactions among different species are key examples of microbial interactions, where microbes exchange electrons either via soluble molecules or directly from cell to cell in an interdependent way. ML was recently used to predict the type of syntrophic interaction that prevails in microbial communities by using a Bayesian network approach [76]. This analysis incorporated not only 16S rRNA sequencing but also metagenomics and metatranscriptomics data.

### Navigating the frontier: challenges and future horizons in AI innovation

AI in biology research faces several major obstacles that must be addressed through close collaboration between biologists and computer scientists. Such interdisciplinary collaborations are essential to exploit the full potential of AI in life sciences [77].

#### Tackling technical challenges in AI-based research

A summary of topics that represent challenges in AI-based research is provided in Table 1. For each topic, the description and its connection to ML and/or DL are highlighted. Additionally, the topics are characterized based on 7 main technical challenges: (i) noisy datasets, (ii) high dimensionality, (iii) omics data integration, (iv) interpretability, (v) computational requirements, (vi) FAIR (Findable, Accessible, Interoperable, Reusable) principles, and (vii) data size and diversity.

**Table 1:** Major technical challenges in AI-based research

Technical challenge	Description	Connection to ML and DL
**1. Noisy datasets**		
*Impact on model performance*	Noisy or erroneous data can degrade AI model performance, leading to inaccurate predictions, especially in high-precision fields like life sciences.	**ML**: Often struggles with noisy data unless advanced preprocessing is applied. **DL**: Sensitive to noise, impacting performance.
*Data cleaning*	Effective noise reduction and robust data cleaning are essential but challenging, particularly at large scales.	**ML**: Requires preprocessing techniques to handle noisy data. **DL**: Needs data cleaning to improve model accuracy.
**2. High dimensionality**		
*Curse of dimensionality*	High-dimensional data can lead to overfitting, making models perform well on training data but poorly on unseen data.	**ML**: Can overfit if dimensionality is not managed; requires feature selection. **DL**: Needs strategies to handle high dimensions.
*Feature selection*	Identifying relevant features from a large number of variables is complex and requires advanced techniques to prevent redundancy and enhance model efficiency.	**ML**: Involves sophisticated techniques for effective feature selection. **DL**: Uses embedded feature selection or reduction techniques.
**3. Omics data integration**		
*Heterogeneity*	Omics data from various sources (e.g., genomics, proteomics) are often heterogeneous, differing in scale, format, and noise, complicating integration.	**ML**: Requires methods to handle heterogeneous data. **DL**: Needs effective data fusion strategies for multiomics.
*Data fusion*	Developing methods for effective multiomics data fusion that preserves biological context and relationships is an ongoing challenge.	**ML**: Must integrate diverse data types. **DL**: Benefits from advanced fusion techniques for comprehensive analysis.
**4. Interpretability of results**		
*Complex models*	Deep learning models, especially those with complex architectures, can act as “black boxes,” making it hard to interpret how conclusions are reached.	**ML**: Generally more interpretable than DL but still faces challenges. **DL**: Requires explainability techniques for transparency.
*Explainability techniques*	Emerging techniques like sHapley additive exPlanations (SHAP) or local intrepretable model-agnostic explanations (LIME) offer ways to explain AI decisions but may not always provide comprehensive or intuitive insights.	**ML**: May utilize various explainability methods. **DL**: Needs specific techniques for understanding model behavior.
**5. Computational requirements**		
*Resource intensity*	Training state-of-the-art AI models, particularly deep learning models, requires significant computational resources, including high-performance GPUs and extensive memory.	**ML**: Generally less resource-intensive but can still require significant computational power. **DL**: Highly resource-demanding.
*Scalability*	Ensuring algorithms scale efficiently with increasing data sizes and complexity without excessive computational costs is a critical challenge.	**ML**: Needs to manage scalability efficiently. **DL**: Must handle large-scale data and complex models effectively.
**6. Importance of FAIR principles**		
*Findable, Accessible, Interoperable, Reusable (FAIR)*	Adhering to FAIR principles for data and scripts is essential for reproducibility and collaboration but challenging, particularly in standardizing metadata and documentation.	**ML**: Requires well-documented datasets for reproducibility. **DL**: Benefits from FAIR practices for consistent data use.
*Data sharing*	Facilitating access to well-documented, standardized datasets while maintaining privacy and security can be complex.	**ML**: Needs secure and standardized data-sharing practices. **DL**: Requires access to high-quality, FAIR-compliant datasets.
**7. Data size and diversity**		
*Scalability of models*	Handling and processing large-scale datasets requires models that can manage and learn from vast amounts of data without compromising performance.	**ML**: Must be scalable to handle large data. **DL**: Efficiently manages large datasets but with high computational costs.
*Bias and generalization*	Ensuring data diversity to avoid biases and ensure models generalize well across different populations or conditions is crucial. Imbalanced datasets can lead to skewed results.	**ML**: Needs diverse data to prevent bias. **DL**: Requires careful data handling to ensure generalization across conditions.

Importantly, data curation and integration across biological subdisciplines continue to pose significant challenges, requiring the development of new theories and predictive models tailored to biology [77]. A significant problem is the lack of standardized formats across different biological disciplines, which not only complicates the handling of file formats [78] but also makes it difficult to interpret data generated by specialists of each omics data type. Ethical concerns, particularly for animal sciences, and privacy issues surrounding data usage need to be addressed, along with ensuring the reliability and safety of AI models through robust validation and transparency. The explainability of AI methods in biological data science remains a significant challenge, as many current approaches lack interpretability. This can lead to decreased trustworthiness and reliability in decision-making processes. Moreover, improving the interpretability of ML-based models in life science is crucial, as it allows a better understanding of the biological mechanisms behind the models (e.g., by helping to identify important biomarkers, biological pathways, or features that contribute to a specific process) [79].

#### The scarcity of labeled data for training AI models

Labeling large amounts of data has become one of the main bottleneck in the development of AI systems [80]. Over the past 15 years, advanced ML models, particularly those based on deep neural networks (DNNs), have enabled unprecedented results in a variety of fields, including omics research in life sciences [81]. However, these models require vast amounts of labeled training data, which in many practical scenarios are either unavailable or very arduous to obtain [82, 83]. Creating hand-labeled training datasets is expensive and time-consuming, often taking months or years to develop, particularly when domain expertise is required. In response to this technical challenge, a subfield of ML know as *weakly supervised learning*, a concept developed back in the 1960s, has evolved into an approach capable of generating large training datasets more rapidly. These datasets, though noisier and of lower quality, are constructed via strategies such as using cheaper annotators, programmatic scripts, or more creative and high-level input from domain experts. In principle, these techniques offer higher-level or less precise forms of supervision, which, while less accurate, are faster and easier to obtain than manual annotation [84]. Another approach motivated by the same goal is *semi-supervised learning*, which strives to create large training datasets by combining a small amount of labeled data with a much larger amount of unlabeled data [85]. Omics-based research in life sciences has quickly adopted solutions derived from these approaches across various applications, such as molecular pathways status prediction in cancer [86] or protein-DNA binding prediction [87], and in the field of plant sciences (applications specific to plant and field phenomics) [88–91]. These examples provide evidence of the effectiveness of *weakly* and *semi-supervised learning* when applied to omics science and indicate a promising future direction.

#### AI for the prediction and annotation of metabolites

Recent developments in AI-based metabolite annotation reflects significant advancements in the application of ML and DL techniques to improve the accuracy and efficiency of metabolite identification and characterization in mass spectrometry–based studies [92]. As an example, the chemical language model “DeepMet” utilizes CNNs to learn features from raw MS/MS spectral data and predict human metabolite identities [93]. Similarly, the “MetFID” model uses ANNs to predict molecular fingerprints from MS/MS data, enhancing annotation accuracy compared to existing tools [94]. Computational annotation strategies, including peak grouping, ion adduction analysis, and incorporation of biological knowledge, help overcome the limitations of accurate mass searching alone [95]. ML-based approaches and molecular networking have shown promise in large-scale metabolite annotation, particularly in natural product discovery [96]. Another compelling ML-based tool includes the “PeakDecoder” algorithm, which enables metabolite annotation and accurate profiling in multidimensional mass spectrometry measurements [97]. Despite the availability of ML-based tools for metabolite annotation, inconsistencies in their benchmarking hinder users from selecting the most appropriate method for their research, highlighting the need for standardized evaluation practices [96].

In the context of ecosystem metabolomics, computational methods can now predict previously unobserved metabolites in new microbial communities by leveraging paired metabolome and metagenome data, achieving over 50% accuracy for related metabolites [98]. Additionally, knowledge-based and ML-driven approaches are being developed to refine metabolite identification and analyze primary microbial metabolism in mixed samples [99]. This demonstrates that predictive metabolomics can aid experimental design and reveal valuable insights into numerous community profiles where only metagenomic data are available.

#### AI-based gene annotation

Advances in genomics have been largely driven by the increasing throughput and lower cost of DNA sequencing. This has made it possible to sequence thousands of individual genomes within a species and a large number of new species. While generating sequencing data has become a relatively straightforward task, the subsequent processing steps to produce a genome assembly with structural annotations of genomic elements (e.g., genes, promoters, and regulatory elements) and gene functional annotations still represent a challenge. Long-read sequencing technologies have alleviated some of these issues, particularly for genome assembly, but the structural annotation of genes, especially in novel genomes, remains problematic in the absence of other extrinsic data sources. Well-known structural annotation tools, such as AUGUSTUS [100], use hidden Markov models (HMMs) for intrinsic *ab initio* gene finding. A recent *ab initio* gene calling tool, Helixer [101], uses DNNs combined with HMMs to identify genes in genomes without the need for extrinsic data and has shown promising results. Gene functional annotation has traditionally relied on homology to characterize proteins for ascribing a function to newly identified genes. The bottleneck of this methodology is mainly due to knowledge gaps that are producing annotation of genes of “unknown function.” DeepGO [102] is a tool that employs DL methods and interactive networks to annotate protein sequences with Gene Ontology (GO) terms. A later improvement, DeepGOPlus [103] removed many of the restrictions of the earlier version and no longer needs the interaction networks. DeepGOPlus has the additional advantage of being species agnostic and gives equally good results from protein sequences derived from genomes of newly sequenced species and clades.

#### FAIR practices for omics data and AI

Despite the advances outlined above, challenges in standardizing methods and interpreting results persist, highlighting the need for FAIR practices and proper benchmarking to ensure reproducibility and reliability in multiomics and AI research. In this context, ontologies play a crucial role by tagging datasets with metadata, thereby improving data understanding and interoperability [104]. They define domain-specific concepts and relationships, making data both human- and machine-readable for easier reuse. However, identifying relevant ontologies can be difficult due to the large amount available. For example, as of September 2024, 1,147 different ontologies are available in BioPortal [105], including 24 specific for plants and 37 for animal science. As ML becomes increasingly indispensable, ensuring data privacy, algorithmic fairness, and transparency will be paramount for maintaining public trust and ensuring equitable access to the benefits of ML-driven advancements [106]. Additionally, many open data sources in the life sciences are not yet fully FAIR-compliant, with issues related to the absence of proper metadata, inadequate data documentation, and the lack of crosslinking between datasets. This requires significant effort to upgrade their FAIRness for integration into semantic web platforms [107]. While the FAIR principles aim to enhance machine readability and processing of scientific data, concerns have been raised about potential epistemic losses, such as a reduction in semantic freedom and the displacement of human expertise, which could discourage trust in AI [108]. To address skepticism and foster trust among stakeholders, a more balanced discussion of both the benefits and epistemic costs of implementing FAIR is needed. Remarkably, a systematic review of 124 LC/MS metabolomics software that subsequently retained 61 for detailed analysis based on FAIR Principles for Research Software (FAIR4RS) criteria reported that software fulfillment of these criteria ranged from 21.6% to 71.8%, with no significant improvement in FAIRness over time [109]. Key issues identified included the lack of semantic annotation (0%, i.e., no software had semantic annotation of key information), low registration on Zenodo with DOIs (6.3%), limited containerization of code or use of virtual machines (14.5%), and insufficiently documented functions in code (16.7%). This recent work highlights clear caveats that need to be addressed in further Big Data–based life science research. To further advance the FAIR principles, collaboration between researchers, data scientists, and data managers is more than ever needed.

### Concluding remarks

In conclusion, AI has already transformed biomedical research by accelerating drug discovery, enhancing clinical trials, and providing powerful tools for analyzing complex biological data [110]. Its ability to optimize processes, reduce costs, and increase precision is revolutionizing how researchers approach biological challenges. The 2020s is the decade of AI applied to biology: as AI continues to advance, its impact on animal, plant, and environmental research will be paramount. AI is reshaping animal research by enhancing data analysis, improving animal welfare, and reducing reliance on traditional testing methods. Through predictive modeling, AI helps refine experimental designs, minimizing the number of animals used while increasing the accuracy of results. It also supports the monitoring of animal behavior and health, contributing to better care and more ethical practices. The growing role of AI in animal research will likely lead to more humane, efficient, and scientifically robust studies. Additionally, the evolution of ML in plant biology, ranging from its early explorations to its current prominence as a transformative tool, demonstrates its remarkable potential. As ML continues to advance, its integration with other AI techniques, real-time data processing, and ethical considerations, including agroecological transitions, will shape the future of plant biology research and agricultural practices. In a wider context, AI is making significant strides in environmental research by providing sophisticated tools for monitoring ecosystems, predicting climate patterns, and analyzing environmental data. Its ability to process vast amounts of information and identify complex patterns helps in understanding and mitigating the impacts of climate change, pollution, and habitat loss. AI promises to enhance our capacity for environmental stewardship, driving more effective and data-driven strategies to protect and sustain our planet.

## Abbreviations

AI: artificial intelligence; ANN: artificial neural network; cDNA: complementary DNA; CNN: convolutional neural network; DL: deep learning; DNN: deep neural network; ESI: electrospray ionization; GC: gas chromatography; GO: Gene Ontology; GP: genomic prediction; GTB: gradient tree boosting; HMM: hidden Markov model; HR: high resolution; LC: liquid chromatography; LIME: local intrepretable model-agnostic explanations; MALDI: matrix-assisted laser desorption ionization; ML: machine learning; MS: mass spectrometry; NGS: next-generation sequencing; OPLS-DA: orthogonal partial least squares discriminant analysis; RF: random forest; RNA-seq: RNA sequencing; SHAP: sHapley additive exPlanations; SNP: single nucleotide polymorphic; SVM: support vector machine.

## Supplementary Material

giaf057_Authors_Response_To_Reviewer_Comments_original_submission

giaf057_GIGA-D-24-00489_original_submission

giaf057_GIGA-D-24-00489_Revision_1

giaf057_Reviewer_1_Report_Original_submission C. Titus Brown -- 1/13/2025

giaf057_Reviewer_2_Report_Original_submission Andrew French -- 2/10/2025

## Data Availability

No data are associated with this article.
