# Supplementary material for: Artificial intelligence: the human response to approach the complexity of big data in biology
Source: Gigascience. 2025 Jun 12;14:giaf057. doi: 10.1093/gigascience/giaf057 (PMC12160488; doi:10.1093/gigascience/giaf057)

GigaScience

Artificial Intelligence: the human response to approach the complexity of big data in biology

--Manuscript Draft--

**Manuscript Number:**	GIGA-D-24-00489R1
**Full Title:**	Artificial Intelligence: the human response to approach the complexity of big data in biology
**Article Type:**	Review
**Funding Information:**	Horizon 2020 Framework Programme (GLOMICAVE 952908) — Dr Biotza Gutierrez Arechederra<br>ANR (MetaboHUB ANR-11-INBS-0010) — Dr Pierre Pétriacq<br>ANR (PHENOME ANR-11-INBS-0012) — Dr Pierre Pétriacq<br>'la Caixa' Foundation (LCF/BQ/PR21/11840001) — Dr Xavier Domingo-Almenara
**Abstract:**	Since the late 2010s, artificial intelligence (AI), encompassing machine learning (ML) and propelled by deep learning (DL), has transformed life science research. It has become a crucial tool for advancing the computational analysis of biological processes, the discovery of natural products, and the study of ecosystem dynamics. This review explores how the rapid increase in high-throughput omics data acquisition has driven the need for AI-based analysis in life sciences, with a particular focus on plant sciences, animal sciences and microbiology. We highlight the role of omics-based predictive analytics in systems biology and innovative AI-based analytical approaches for gaining deeper insights into complex biological systems. Finally, we discuss the importance of FAIR (findable, accessible, interoperable, reusable) principles for omics data, as well as the future challenges and opportunities presented by the increasing use of AI in life sciences.
**Corresponding Author:**	Pierre Pétriacq, PhD, HDR<br>UMR1332: Biologie du Fruit et Pathologie<br>Villenave d'Ornon, FRANCE
**Corresponding Author Secondary Information:**	
**Corresponding Author's Institution:**	UMR1332: Biologie du Fruit et Pathologie
**Corresponding Author's Secondary Institution:**	
**First Author:**	Giovanni Melandri
**First Author Secondary Information:**	
**Order of Authors:**	Giovanni Melandri
	Georges R-Radohery
	Chloé Beaumont
	Sara M. de Cripan
	Coralie Muller
	Luca Piras
	Maria Alcina Pereira
	Andreia Salvador
	Xavier Domingo-Almenara
	Marie Bolger

Powered by Editorial Manager® and ProduXion Manager® from Aries Systems Corporation

	Sophie Colombié
	Sylvain Prigent
	Biotza Gutierrez Arechederra
	Núria Canela Canela
	Pierre Pétriacq, PhD, HDR
**Order of Authors Secondary Information:**	
**Response to Reviewers:**	Dear Editor,<br>Dear Reviewers,<br><br>Thank you for your insightful comments, which have significantly contributed to the enhancement of our manuscript. We have carefully addressed all the reviewers' comments. Additionally, the manuscript has been proofread by a native English speaker to ensure consistency in language quality throughout. We have also verified the cited references to correct any duplication errors that may have been present in the previous version. The revision includes the manuscript with track changes, and a clean version without track changes.<br><br>Sincerely,<br>Dr Pierre Pétriacq, on behalf of all the authors<br><br>p5 Several algorithms from ML are listed with little explanation and few references.<br>- We have revised this section of the manuscript, explaining in more detail the algorithms mentioned and providing additional references (L149-L209 of the revised manuscript).<br><br>p6 Diagram. Why is Omics fully inside deep learning?<br>- Thanks for this valuable verification. We have revised Figure 2, with AI and omics being connected concepts of Data sciences.<br><br>p6 L154 "Advanced DL-based models represent the state of the art" - can you provide a few example references to justify this?<br>- We have provided two references on the role of deep learning algorithms as the most advanced for modelling in biological sciences (L225 of the revised manuscript).<br><br>P7 text section. This needs a proof read, some of the wording is off.<br>- We revised this text section to improve its readability (L237-256 of the revised manuscript).<br><br>P7 Figure 3. The arrows pointing to the bottom row don't make much sense - e.g. Weakly supervised learning doesn't point anywhere?<br>- Thank you for the comment. We have included a revised figure 3 that should address this comment.<br><br>p8 I believe GLOMICAVE is the work of the authors? Therefore, the review should be clear about this and why it is likely mentioned.<br>- We have explained that GLOMICAVE is a project involving all the authors of the review article (L262-263 of the revised manuscript).<br><br>p9 Fig 4. I don't really see the value in this figure as it stands?<br>- We thank you for this valuable verification. We also agree that this figure lacks relevance and have therefore removed it from the revised manuscript.<br><br>p9 L210 "The only feasible strategy" - I don't agree with this statement<br>- We have changed it into “a practical and effective strategy” (L284-285 of the revised manuscript).<br><br>P268 "Microbial Ecology Sciences" - the title of section seems over specific given the broadness of some of the others?

	- We have changed the title of the section to “Microbial Sciences and AI” to make it less specific (L351 of the revised manuscript).<br><br>p14 L329 Weak supervision is not an emerging field. It has been around for a long time.<br>- We have removed the term ‘emerging’ from the description of weakly supervised learning and explained that it is an old concept that has been fully exploited in ML over the last 10 years (L423-426 of the revised manuscript).<br><br>p17 L407 I assume 0% is a typo<br>- We have double-checked the publication and 0% is real, not a typo (https://link.springer.com/article/10.1007/s11306-023-01974-3). For improved clarity, we have precised L507: “0%, i.e. no software had semantic annotation of key information”.
**Additional Information:**	
**Question**	**Response**
Are you submitting this manuscript to a special series or article collection?	No
**Experimental design and statistics**<br><br>Full details of the experimental design and statistical methods used should be given in the Methods section, as detailed in our Minimum Standards Reporting Checklist. Information essential to interpreting the data presented should be made available in the figure legends.<br><br>Have you included all the information requested in your manuscript?	No
If not, please give reasons for any omissions below.<br><br>as follow-up to "**Experimental design and statistics**<br><br>Full details of the experimental design and statistical methods used should be given in the Methods section, as detailed in our Minimum Standards Reporting Checklist. Information essential to interpreting the data presented should be made available in the figure legends.<br><br>Have you included all the information requested in your manuscript?	This is a review manuscript, without Methods section.

Powered by Editorial Manager® and ProduXion Manager® from Aries Systems Corporation

"	
**Resources**<br><br>A description of all resources used, including antibodies, cell lines, animals and software tools, with enough information to allow them to be uniquely identified, should be included in the Methods section. Authors are strongly encouraged to cite Research Resource Identifiers (RRIDs) for antibodies, model organisms and tools, where possible.<br><br>Have you included the information requested as detailed in our Minimum Standards Reporting Checklist?	No
If not, please give reasons for any omissions below.<br><br>as follow-up to "**Resources**<br><br>A description of all resources used, including antibodies, cell lines, animals and software tools, with enough information to allow them to be uniquely identified, should be included in the Methods section. Authors are strongly encouraged to cite Research Resource Identifiers (RRIDs) for antibodies, model organisms and tools, where possible.<br><br>Have you included the information requested as detailed in our Minimum Standards Reporting Checklist?<br><br>"	This is a review manuscript, without Methods section.
**Availability of data and materials**<br><br>All datasets and code on which the conclusions of the paper rely must be either included in your submission or deposited in publicly available repositories (where available and ethically appropriate), referencing such data using	No

a unique identifier in the references and in the "Availability of Data and Materials" section of your manuscript.<br><br>Have you have met the above requirement as detailed in our Minimum Standards Reporting Checklist?	
If not, please give reasons for any omissions below.<br><br>as follow-up to "**Availability of data and materials**<br><br>All datasets and code on which the conclusions of the paper rely must be either included in your submission or deposited in publicly available repositories (where available and ethically appropriate), referencing such data using a unique identifier in the references and in the "Availability of Data and Materials" section of your manuscript.<br><br>Have you have met the above requirement as detailed in our Minimum Standards Reporting Checklist?<br><br>"	This is a review manuscript, without Methods section.
GigaScience has policies and guidelines in place for the use of generative AI-writing tools such as ChatGPT. If you have used such writing tools to assist with writing the manuscript this must be declared and cited in the text. Authors should not list AI-writing tools and other AI-assisted technologies as an author or co-author and should acknowledge that they are fully responsible for text generated or refined by AI-writing tools.<p><br><br>A summary of use (particularly in the introduction or among methods) needs to be included at the end of the paper, and the outputs should also be included as a supplementary file hosted in GigaDB or other open repositories. Please read our guidelines for more information. <p>	No

By submitting to GigaScience, you are aware of the journal's AI-writing tools policy, and if you have declared use of such tools below, you have acknowledged this where appropriate in your manuscript and have made a summary of use and outputs available. <p> <b>AI-assisted writing tools have been used in the preparation of this manuscript?	

Powered by Editorial Manager® and ProduXion Manager® from Aries Systems Corporation

Manuscript_with_track_changes
Click here to access/download;Manuscript;20250402_AI_review_GigaScience

Artificial Intelligence: the human response to approach the complexity of big data in biology

Giovanni Melandri [1,9,♪], Georges R-Radohery [1,♪], Chloé Beaumont [1], Sara M. de Cripan [7], Coralie Muller [1], Luca Piras [2], Maria Alcina Pereira [4,5], Andreia Ferreira Salvador [4,5], Xavier Domingo-Almenara [7,8], Marie Bolger [6], Sophie Colombié [1,3], Sylvain Prigent [1,3], Biotza Gutierrez Arechederra [2], Nuria Canela Canela [7], Pierre Pétriacq [1,3]*

[1] *Univ. Bordeaux, INRAE, UMR1332 BFP, 33140 Villenave d'Ornon, France*
[2] *EURECAT - Technology Centre of Catalonia, Barcelona, Catalonia, Spain*
[3] *Bordeaux Metabolome, MetaboHUB, PHENOME-EMPHASIS, 33140 Villenave d'Ornon, France*
[4] *Centre of Biological Engineering, University of Minho, 4704-553, Braga, Portugal*
[5] *LABBELS – Associate Laboratory, Braga/Guimarães, Portugal.*
[6] Institute of Bio- and Geosciences, IBG-4: Bioinformatics, Forschungszentrum Jülich, CEPLAS, BioSC, Jülich, Germany
[7] *Centre for Omics Sciences (COS), Eurecat - Technology Centre of Catalonia & Rovira i Virgili University joint unit, Unique Scientific and Technical Infrastructures (ICTS), Reus, Catalonia, Spain*
[8] *Department of Electrical, Electronic and Control Engineering (DEEEA), Universitat Rovira i Virgili, Tarragona, Catalonia, Spain*
[9] *School of Plant Sciences, University of Arizona, Tucson, USA*

♪ Equal contribution
* Author for correspondence: pierre.petriacq@inrae.fr

ORCID:

Giovanni Melandri	0000-0002-0877-5009
Georges R-Radohery	0000-0003-1405-3106
Maria Alcina Pereira	0000-0002-7110-1779
Andreia Salvador	0000-0001-6037-4248
Xavier Domingo-Almenara	0000-0002-0133-6863
Marie Bolger	0000-0001-6335-1578
Sophie Colombié	0000-0002-9810-4339
Sylvain Prigent	0000-0001-5146-0347
Biotza Gutierrez Arechederra	0000-0001-7411-2580
Núria Canela Canela	0000-0003-0261-2396
Pierre Pétriacq	0000-0001-8151-7420

ABSTRACT

Since the late 2010s, artificial intelligence (AI), ~~which~~ encompassing~~es~~ machine learning (ML) and ~~is~~ propelled by deep learning (DL), has ~~revolutionised~~transformed life science research. It has become ~~as~~ a crucial tool for advancing the computational ~~evaluation~~analysis of biological processes, the discovery of natural products, and the study of ecosystem ~~functioning~~dynamics. This~~e present~~ review ~~aims to describe~~explores how the ~~dramatic~~rapid increase in high-throughput omics data acquisition has

~~necessitated the application~~driven the need for~~ of~~ AI-based analysis in life sciences, with a particular focus on plant sciences, animal sciences and microbiology. ~~In particular, w~~We highlight the role of omics-based predictive analytics in systems biology and innovative AI-based analytical approaches for ~~a better understanding of~~gaining deeper insights into complex biological systems. Finally, we discuss the importance of FAIR (findable, accessible, interoperable, reusable) principles for omics data, as well as~~ and~~ the future challenges and opportunities ~~associated with~~presented by the increasing use of AI in life sciences.

Keywords: artificial intelligence, machine learning, deep learning, omics, life science, biology

BACKGROUND

The explosion of omics requires Artificial Intelligence in the study of life sciences

In the past two decades, research and society have ~~been living in~~entered the 'big data' era of life sciences. Technological advances have ~~continuously increased~~enhanced our ability to measure qualitative and quantitative variations of internal biological molecules (e.g., DNA, RNA, proteins, metabolites) and phenotypes, making the acquisition of large and complex omics datasets within a single experiments increasingly common.

The explosion of omics data in life sciences ~~started~~began with genomics ~~and~~which was ~~enabled~~driven by the ~~advent~~emergence of DNA Next-Generation Sequencing (NGS) platforms~~,~~ nearly 20 years ago. ~~Since~~While the groundbreaking discovery of the Sanger DNA sequencing method dates back to ~~in~~ the 1970s, it ~~was~~took three decades ~~later that~~for the advent of second-generation short-read sequencing-based NGS to further revolutionise DNA sequencing, ~~provided a giant leap forward in the~~dramatically increasing its affordability and throughput~~ of DNA sequencing~~. This has led to the *de novo* assembly of thousands of animal and plant genomes [1,2] and to the discovery of millions of genome-wide single nucleotide polymorphic (SNP) variants . High-throughput analysis of multiple gene transcripts (i.e., transcriptomics) ~~started~~began in the mid-1990s with the introduction of hybridisation-based microarray technologies. However, it was ~~only from~~not until the 2000s that NGS enabled a more accurate ~~estimation~~assessment of the qualitative and quantitative diversity (e.g., large dynamic range of expression

levels and alternative splicing variants) of messenger RNAs~~ (mRNAs) was enabled by NGS~~. This technique, known as RNA sequencing (RNA-seq), uses NGS to sequence ~~transcript~~ complementary DNAs (cDNAs) derived from RNA transcripts [3,4]. The current third-generation single molecule sequencing technologies (e.g., PacBio and Oxford Nanopore Technologies) have further improved the read length, throughput, and ~~affordability~~accuracy of data collection in the field of genomics and transcriptomics [5,6]. The field of proteomics and metabolomics relies on the use of mass spectrometry (MS) techniques to explore the diversity of proteins and metabolites in both a qualitative and quantitative manner. Although mass spectrometers have been available since the late 1940s, it was their integration with gas or liquid chromatography (GC and LC) and the development of ionisation techniques ~~like~~such as electrospray ionisation (ESI) and matrix-assisted laser desorption ionisation (MALDI) in the late 1980s that truly expanded their application to biological research [7,8]. There are various ionisation techniques in mass spectrometry and electronic impact ionisation that, while historically important for profiling primary compounds of biological samples, have~~s~~ been largely superseded by softer ionisation methods such as ESI and MALDI. These newer techniques are more suitable for analysing biomolecules as they cause less fragmentation and tend to preserve the integrity of molecules during ionisation. ~~Within~~Over the past~~ the last~~ twenty years, the ~~development~~advancement of high-resolution (HR) MS has been crucial ~~to provide a massive boost in~~in significantly enhancing the identification of proteins and metabolites. This has ~~opening up to the large~~driven the widespread application of proteomics and metabolomics in the analysis of complex biological samples [9,10].

Recent advancements in imaging technologies have ~~significantly enhanced~~improved life science research, benefiting not only the medical field [11], but also~~ in~~ plant sciences. The ~~sub~~field of plant/crop phenomics has rapidly evolved ~~thanks~~due to ~~advances~~breakthrough~~t~~s in sensor technology, machine vision, and automation technology [12]. Today, automated, non-invasive, high-throughput imaging and sensor technologies ~~have~~ generate~~d~~ vast~~ an overwhelming~~ amounts of image and sensor data presenting both opportunities and challenges for analysis. ~~.~~

The ~~technology-mediated~~ ability to generate high-throughput large-scale omics data through advanced technologies offers an unprecedented opportunity for~~ in-depth~~ exploratin~~m~~g~~on~~ ~~of~~ the complexity of biological systems in depth. Furthermore, ~~acquiring~~integrating multiple omics datasets from a single

experiment ~~allows the adoption o~~facilitates a ~~f a~~ ‘holistic’ approach which the potential of~~ly~~ ~~enables the understanding of~~revealing how the ‘molecular endophenome’ (at cellular/tissue level) is regulated and connected with the ‘external phenome’ of biological organisms. However, ~~D~~disentangling and deciphering the ~~complexity hidden~~intricate relationships among ~~in the simultaneous variation of~~ tens of thousands (sometimes millions) of molecular variables (i.e., SNPs, transcripts, proteins, and metabolites), which are interconnected ~~with each other~~among themselves and with the final phenotype, has been a major challenge in biological research ~~during the last 20 years~~over the past two decades [13,14]. The use of high-dimensional solutions ~~and~~on complex omics datasets ~~for~~to address~~ing~~ fundamental biological questions ~~is a task that surpasses the analytical abilities~~exceeds the capacity of the human brain. This requires a computer-based analytical approach, which can benefit from the constant improvements in~~of the computational power of~~ machine~~s~~ processing power at all levels (single machine or physical/cloud-based clusters). For these reasons, ‘artificial intelligence’ (AI) has ~~become prominent~~emerged as a key tool in ~~the study of~~ life science~~s~~ research (Fig. 1) with the ~~foreseeable scenario~~expectation that AI will lead or assist in most of the future biological discoveries~~ in the field of biology~~.

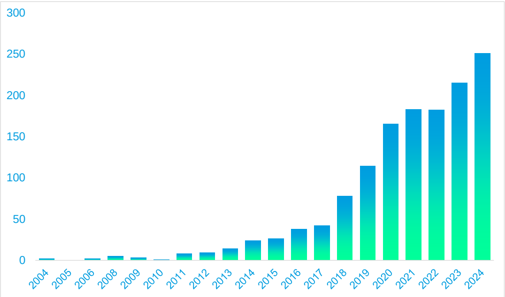

Figure 1. Number of publications ~~searched~~found in PubMed including [artificial intelligence] AND [omics] AND [life sciences] from 2004 to 2024. In total, 1362 publications were found (Sept 19th 2024). Considering the last 20 years, a literature search using the queries [omics] AND [artificial intelligence] AND [life sciences] confirms that AI in life sciences is a rapidly expanding field of research.

Artificial Intelligence, Machine Learning and Deep Learning

Despite its widespread use, the term AI remains an elusive ‘buzzword’. From a scientific perspective, the difficulty in defining AI is associated with the complexity of the concept of intelligence *per se* and with the fact that, despite resurgence of interest in AI started in the 1990s, fast progresses in AI research rapidly developed only from the 2010s~~is only at its beginning~~ and, thus, this field of research is far from reaching a level of maturity that can be translated into a clear definition [15].

Oversimplifying, AI can be considered as a branch of computer science ~~that aims at~~focused on programming ~~a~~ machines (~~essentially~~typically one or more computers) to perform a ~~single~~specific tasks by learning from the information present in specific dataset(s) [16] (Fig. 2). This definition is appropriate only for ‘Artificial Narrow Intelligence’ or ‘Weak AI’, ~~the one that~~which is currently used for many routine and nearly ubiquitous ~~routine~~ applications such as spam filtering, speech recognition, language translation, online advertising, image tagging, etc. However, this ~~The same~~ definition is not accurate for ‘Artificial General Intelligence’ or ‘Artificial Super Intelligence’ which are both still far from being achieved. These forms of AI aim to ~~and are targeted at develop~~developing machines ~~with the ability to~~ capable of learning and understand from data in ways that are comparable~~similar,~~ to or surpass~~perior, to~~ human intelligence [17].

Considering ‘Artificial Narrow Intelligence’ (hereafter AI will refer to this term) and, particularly, its most popular subfield ‘Machine Learning’ (ML), the ‘learning’ feature defines the process of using an algorithm which finds complex patterns in the training data and translates them into an object-level algorithm (such as a model of a domain problem) which, in turn, is able to make predictions about unobserved data. It is in the context of ML that biological research has ~~benefitted~~benefited the most from the use of large and complex omics data [18,19]. Biological data-based ML models have the double target of (1) accurately predicting experimental data and (2) using this predicting ability to inform and direct the efforts of future research. When developing ML models, the characteristics of the training data determine the learning approach. ~~Essential for developing ML models is the combination of ‘data type’ and the kind of ‘algorithm’ used~~. Training data refers to the dataset used to teach an ML model. ~~If the training data that inform the algorithm are not labelled (no tags), the ML model is defined as based on ‘unsupervised’ learning~~ and a key distinction is whether these training data includes annotations, which determine the learning method applied. Training data can be ~~labelled~~labeled or unlabe~~l~~led. ~~Labelled~~Labeled data contains explicit tags,

such as categories or numerical (values, allowing the model to learn from predefined outcomes. When the data are labelledlabeled, the model follows a supervised learning approach. In constrastcontrast, unlabellled data lacks predefined tags, requiring the model to extract patterns and relationships independently— - a process known as unsupervised learning (Fig. 3) [20]. On the contrary, if the same data are labelled (with qualitative or quantitative tags), the ML model is defined as based on 'supervised' learning. Unsupervised ML models are mainly used to deal with clustering problems where the algorithms (e.g., K-means clustering or DBSCAN clustering) find relationships in the overall structure of the training data [20]. In supervised learning the algorithm uses the provided labels as a guide to map data points to specific outcomes or classifications., In contrast, the algorithm independently examine the unlabeled dataset in unsupervised learning and identify patterns, clusters, or relationships without external hints.

Machine learning is built on a few fundamental algorithms that serve as the foundation for more advanced techniques [21] (Domingos, 2015). Here, we focus on a non-exhaustive list of these algorithms, particularly those that are interpretable and can clarify the importance of each variable in making predictions. This interpretability is especially valuable in plantlife sciences, where it allows for a thorough utilization of information found in omics data—such as genomics, proteomics, and metabolomics—to uncover biological insights [22](van Dijk et al., 2020).

First, linear regression, used in supervised learning, predicts a continuous target variable by establishing a linear relationship between inputs and outputs and adjusting parameters to minimise the difference between expected and actual values. Linear regression is highly interpretable, as it establishes a clear linear relationship between input features and the target variable, allowing a straightforward understanding of how changes in each input affect the predicted outcome. The training process involves iterative adjustments to reduce prediction errors, often guided by optimisation techniques [23] (Schober, Boer, & Schwarte, 2018). Linear regression forms the basis for methods like Ridge and Lasso regression, which incorporate penalties to mitigate overfitting, where the model performs well on the training data but poorly on new, unseen data, by constraining model complexity [24][2[24]4] (Swindel, 2013). These extensions enhance robustness and inform the weight adjustment mechanisms central to neural networks, demonstrating its role as a building block in machine learning [25] (Goodfellow et al. 2016)

Formatted: Strikethrough

Commented [GM1]: This is repeating what is written in L141-142

Formatted: Strikethrough

Next, Support Vector Machines (SVMs) address classification by identifying an optimal boundary that maximises the distance to the nearest data points, which are known as support vectors. The support vectors provide insight into which data points are most crucial for the classification boundary. By examining these vectors and their corresponding features, one can infer which aspects of the data are influential in decision-making. For datasets where linear separation is infeasible, SVMs employ kernel functions—such as polynomial or radial basis functions—to transform the data into a higher-dimensional space, enabling complex separations [26]~~(Cortes & Vapnik, 1995)~~. This emphasis on margin maximisation and spatial transformation influences modern deep learning architectures, notably in convolutional neural networks, where kernel-based operations are prevalent [27]. ~~(Mairal et al. 2014)~~

Decision trees, another supervised learning approach, partition the feature space into distinct regions based on threshold values applied to input variables. Criteria that maximize class separation, such as reducing impurity (e.g., Gini index) or minimizing prediction variance for regression tasks, determine these splits. Their interpretability—from clear, rule-based decisions—makes them particularly appealing for applications requiring transparency, such as omics-driven research (Breiman, Friedman, Olshen, & Stone, 1984). Moreover, integrating them into ensemble methods like random forests, where multiple trees vote to enhance accuracy, or gradient-boosted trees, which iteratively refine predictions, amplifies their utility. These ensembles illustrate how decision trees evolve into robust predictive tools [28]. ~~(Friedman, 2001).~~

We take as the last example Naïve Bayes, which offers a probabilistic framework for classification. It assumes that features are independent within each class and uses probabilities to determine the most likely class for a given set of data. By applying Bayes' theorem, it calculates how likely something belongs to a specific category based on past data. Hence, a Naïve Bayes classifier provides probabilities for each class rather than hard classifications. Further~~n~~more each feature's contribution to the final decision can be calculated based on its likelihood of occurrence on each class thus giving a strong interpretability to the model. However, assumption like data independence could be unrealistic for biological data. Naïve Bayes forms the basis for more advanced probabilistic models, like Bayesian networks. A Bayesian network extends the Naïve Bayes classifier by allowing dependencies between variables, unlike Naïve Bayes, which assumes all features are conditionally independent given the class label. It represents a probabilistic

graphical model where nodes (variables) have directed edges (dependencies) between them [29]~~(Pearl, 1988)~~.

~~Supervised ML models are mainly used for classification problems if the training data are labelled with discrete classes or categories, and for regression problems if the data are labelled by a continuous set of values. For both classification and regression ML models, the most common and traditionally used algorithms are linear regressions with or without regularisation (e.g., ridge regression, lasso, elastic net), support vector-based models (SVM or SVR, with both linear and non-linear kernels), tree-based algorithms, such as random forest (RF), and Bayesian classifiers [22,23]. A key feature of these algorithms in the field of life science is their easy interpretability in terms of estimating the importance of each variable for the prediction model, thus allowing a full exploitability of the information carried by omics data.~~

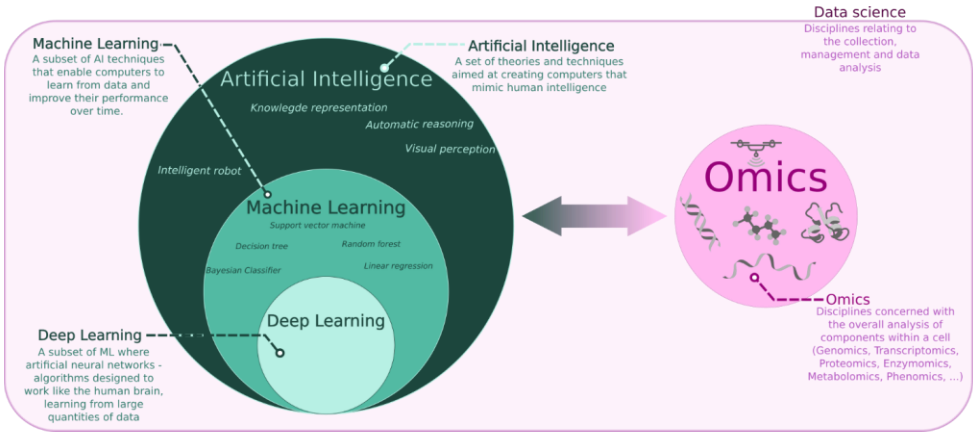

Figure 2. ***Data science in the era of Artificial Intelligence, Machine Learning and Deep Learning: A Dynamic Schematic Breakdown.***

Since its formal introduction in 2006, deep learning (DL) [32], based on diverse artificial neural networks (ANN) algorithms, has further boosted the use of ML in many fields of research, particularly in speech recognition and image analysis [33] but also in the biological field, such as in regulatory genomics and protein classification [34,35] (Fig. 3). Advanced DL-based models represent the state-of-the-art of prediction accuracy in biological sciences [36,37]. Nevertheless, they require the availability of very large-

scale training data (with an associated high computational demand) and their interpretation remains elusive (they are often referred to as 'black-box models'), with this elusiveness representing a limitation in biological experiments involving omics data for which identifying the most important predicting features and feature combinations is of primary importance [38]. Thus, when research is aimed at better understanding the functioning of biological systems, DL-based models are still difficult to be commonly applied [31,39]. It is also for these reasons that in a society where AI algorithms are becoming more central than ever before in all aspects of our daily life, the concepts of 'interpretable ML' and 'explainable AI' are gaining an always increasing attention and importance [35,40].

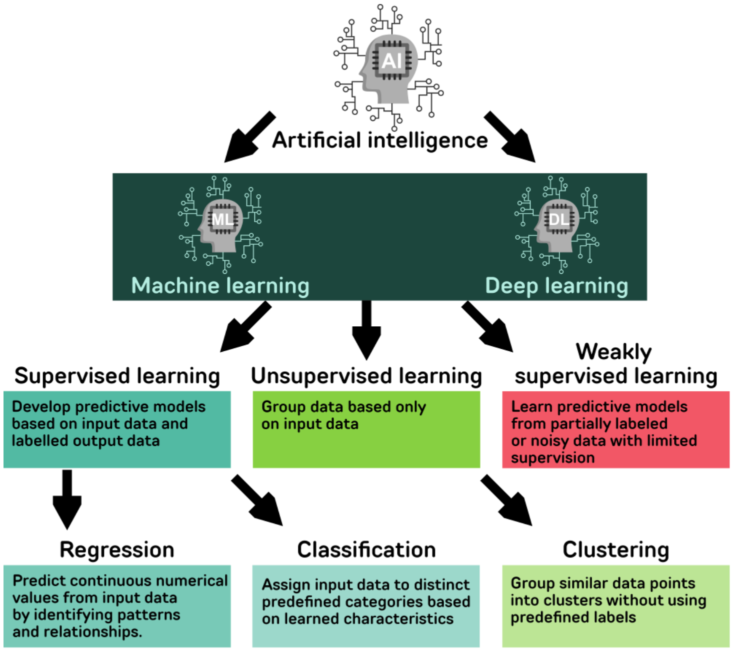

Figure 3. Major approaches in machine learning and deep learning.

Multi-omics integration for ML analysis

As mentioned above, innovations in high-throughput acquisition of different omics data from single experiments are now enabling ~~to~~ capturinge different layers~~high levels~~ of biological complexity. In fact, ~~scaling up~~application of omics approaches, such as transcriptomic, proteomics, and metabolomics [41], to

large diversity panels and/or sample ~~sets~~~~cohort studies holds~~holds significant promise for unravelling the complexity of living systems. Despite their overall potential for discovery~~This complexity can be handled by ML algorithms but~~, the diverse nature of omics data~~,~~ acquired by different technological platforms, requires the use of integration strategies to ~~allow~~ for ~~an effective use of their~~effectively harness their complementary information. Recent advances in multi-omics analysis have ~~only~~ been made possible ~~thanks to~~by the development of various tools and methods ~~able to~~that can resolve the~~for integrating~~ heterogeneous nature of biological datasets~~, thus allowing for~~ enabling their effective integration. Notably, consensus orthogonal partial least squares discriminant analysis (OPLS-DA) has emerged as an effective strategy for fusing ~~multiblock~~ multi-omics data, combining multiple kernel learning with OPLS-DA [42]. The *mixOmics* R package provides a variety of multivariate methods for integrating omics datasets, including extensions of 'Projection to Latent Structure' models for discriminant analysis and molecular signature identification [43]. Additionally, ~~machine learning~~ ML techniques, such as network-based diffusion and DL, are increasingly used to capture complex non-linear associations in multi-omics data [44]. Among the available R resources, packages such *moiraine* (https://plant-food-research-open.github.io/moiraine/) ~~also cover a~~provide a range of integrative methods for multi-omics analyses, including sPLS and DIABLO from the *mixOmics* package [43], sO2PLS from the *OmicsPLS* package [45] and MOFA and MEFISTO from the *MOFA2* package [46].

AI-based analysis of omics data in the fields of Plant Sciences, Animal Sciences, and Microbial Sciences

International initiatives are thriving in the field of AI-based analysis of omics data, aiming to advance the discovery of genotype-phenotype relationships ~~(Fig. 4)~~. One~~An~~ such example is the *GLOMICAVE* project (GLobal OMIC data integration on Animal, Vegetal and Environment sectors), an international project that involves all the authors of this review paper. *GLOMICAVE* ~~which~~ has created an innovative digital platform that connects genotype to phenotype through Big Data Analytics and AI, utilising extensive public and experimental omic datasets (https://glomicave.eu/). Likewise, cloud-based platforms like HiOmics offer a comprehensive analysis of biomedical large-scale omics data [47]. Such projects aim to facilitate the analysis of primary data and support large-scale omics experiments, thereby enhancing the utility of omics data on a massive scale and deepening our understanding of entire biological systems. In line with

GLOMICAVE, and considering that ~~since~~ the medical field has been extensively examined from an AI perspective, this review focuses on relevant applications from plant, animal and microbial sciences.

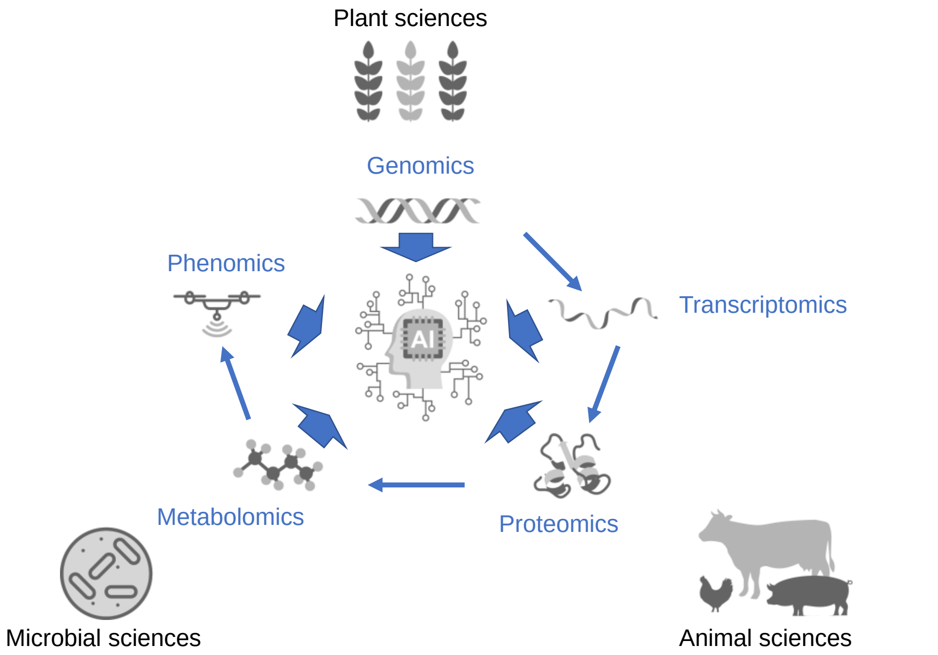

~~***Figure 4. Graphical Representation of Omics Data and Their Integration with AI in Biological Systems.***~~

Plant Sciences and AI

The explosion of omics has radically transformed research in ~~the field of~~ plant sciences ~~and~~, simultaneously~~,~~ driving the need for ~~required the application of~~ ML to ~~deal~~handle ~~with~~ datasets characterised by high complexity and dimensionality. A paradigmatic example is ~~represented by~~ plant phenomics, which ~~just in a few years~~has rapidly shifted from ~~being~~ a promising research sector with the potential of bridging the gap with genomic advances, to becoming a widespread tool in plant and crop sciences [48]. This ~~fast~~rapid progress was enabled by ~~coupling~~integrating ~~single and multiple~~ advanced sensors and imag~~ers~~ing technologies (e.g., RGB, multispectral, hyperspectral, thermal, and fluorescence cameras and sensors)

towith unmanned aerial vehicles (UAVs or drones) and ground robots, which are able to collect high-throughput phenotyping data. Approaches based on ML algorithms are now a practical and effective the only feasible strategy tofor extracting traits and features from massive amounts of imaging- and sensor-based data. DL algorithms (e.g., convolutional neural networks-CNNs) show the highest versatility and success when applied toin image-based plant phenotyping,. These algorithms are particularly effective especially toin predicting the effects of biotic and abiotic stresses [49,50] or forand enabling rapid and accurate diagnostics of plant diseases [51]. Additionally, AI applications in root system architecture image analysis are emerging as crucial tools for improving this understudied field of research, that has thewhich holds significant potential to boost a "Second Green Revolution" in agriculture [52]. Plant breeding is another branch of plant science that washas been radically transformed by genomic advances, with breeders increasingly relying on genome-wide SNP markers-based genomic prediction (GP) to increase the speedaccelerate of genetic gains for target traits in crops. Classic GP models are based on best linear unbiased prediction (BLUP), howeverbut, efforts to develop new ML-based and improved GP algorithms are ongoing [53]. Furthermore, different sources of non-genetic variability, and non-additive modes of gene action have made modelthe choice and implementation of GP models challenging for improving complex plant traits, such as biomass and crop yield [54]. AOne possible solution to this problem is to useincorporate other genome-to-phenome intermediate omics data (e.g., transcriptomics, proteomics, metabolomics) into the for improving GP models to enhance their accuracy and predictive power [55]. The potential of ML models based on single intermediate omics, and particularly metabolomics, the omics layer closer to the phenotype, has been demonstrated for the accurate prediction of crop yield has been demonstrated, notably in maize [56] and rice [57,58]. However, for plant breeding applications, the integration of large, highly dimensional, and 'noisy' omics datasets for complex trait prediction remains a challenging field of study. This challenge that will require the use of ML/DLL techniques, leveraging their and its superior capability for big data analytics to effectively handle the complexity and scale of these datasets [59]. Interestingly, in recent studies have highlighted innovative approaches in, metabolomics-based ML prediction of plant complex traits showedshowing innovative routes to identify breeding targets for plant improvement. For example, Colantonio *et al.* identified candidate metabolites acting as fruit flavor enhancers and suppressors by metabolomics-based ML prediction of tomato and blueberry fruit flavor

profiles [60]. In ~~the context of~~efforts to improve~~ing~~ plant tolerance to abiotic stress, Dussarrat *et al.* ~~performed~~applied a holistic ML prediction approach on~~of~~ environmental adaptations based on the multi-species metabolome of plants collected in the Atacama desert. This revealed a core set of metabolites targets for extreme climate resilience (sugars, stress-related amino acids, hormones and antioxidants including phenolics and major redox buffers) [61].

Animal Sciences and AI

Modern biotechnologies, bio-sensing hardware, and IT infrastructure have led to a high-throughput data collection era in livestock management, ~~pushing~~driving the need for faster and more efficient computational methods. While traditional information sources in animal breeding included phenotype and pedigree ~~information~~data, ~~animal breeding~~the field is ~~now~~ increasingly ~~relying~~incorporating ~~on~~ genomic data such as SNPs, gene annotations, metabolic pathways, protein interaction networks, gene expression, and protein structure information~~,~~. ~~which~~These data can enhance trait predictions and improve our understanding of the underlying biological phenotypes [62]. ~~Although there have been~~Despite these advancements in animal genetics, many challenges still persis~~t~~. The widespread adoption of omics technologies is hindered by high cost and ~~requirement~~the need for expertise across diverse fields~~ hamper the wide adoption of omics technologies~~. ~~Additionally, a~~Accurate recording of phenotypic data and population/sample size are other constraints that need to be addressed. However, ~~the use of~~ omics technologies ~~has~~have shown ~~its~~their potential to identify superior and disease-resistant animals at an early stage [63]. For example, the metabolomes of healthy and unhealthy chickens were characterised and compared using untargeted mass spectrometry metabolomics [64]. ~~They~~Researchers were able to accurately distinguish chicken health status in multiple countries using a~~n~~ random~~R~~ forest ~~F~~-based ML model. This ~~using~~approach utilised raw mass spectrometry signals (unannotated m/z values) as input features, ~~in turn bypassing~~effectively overc~~v~~oming one of the ~~main~~primary limitations of~~in~~ untargeted metabolomics: the need for metabolite annotation and identificatio~~n~~n ~~of metabolites~~. The ~~utilisation~~use of ML models in animal breeding has recently ~~gained~~attracted interest due to their exceptional flexibility and ~~their~~ ability to capture patterns in large, noisy datasets [65]. For ~~example~~instance, Gradient Tree Boosting (GTB) has ~~been used as an accurate~~proven to be an effective ML algorithm for predicting different breeding values. GTB-based models

have~~also~~ identified a subset of genes contributing ~~genes~~ to feed efficiency in growing pigs using muscle transcriptome data [66]. The potential of combining metagenomics, metatranscriptomics and metabolomics data was evaluated in rumen content, demonstrating their value - as predictive markers for feed efficiency and ~~described~~ their potential applications for selecting cows with high feed efficiency [67]. ~~They used an~~By using a RF-based model they were able to predict feed efficiency ~~based on a~~using a preselected set of metabolites associated with this trait. Antimicrobial-resistant microorganisms ~~are a major concern~~pose significant challenges in livestock farming. ~~In a~~A recent study [68] evaluated~~,~~ ten supervised learning classifiers ~~were evaluated~~ to predict *E. coli* strains susceptible ~~and~~or resistant ~~strains of *E. coli* for~~to 26 different antimicrobials using whole genome shotgun sequencing in intensive poultry farming. This ~~study found~~findings provided evidence of transmissible drug resistance in food-producing animals, which has contributed to the emergence of drug resistance in zoonotic pathogens.

Microbial ~~Ecology~~ Sciences and AI

Microorganisms exist naturally in microbial communities and establish multiple interactions between each other and with their hosts. Omics experiments play a crucial role in ~~are central to allow the~~ studying these microorganisms in their ~~of~~ natural~~ly~~ environments, eliminating ~~occurring microorganisms without~~ the need for their isolation and cultivation. However, interpreting omics information and ~~linking~~integrating ~~the~~ results ~~obtained by~~from different studies ~~is still~~remains challenging~~,~~ due to the complexiti~~y~~ of omics data ~~complexity~~. AI has been increasingly applied to help ~~in the interpretation~~interpret ~~of~~the variations ~~present~~found in microbial communities ~~but mostly, although not restricted,~~, particularly in the human microbiome and ~~in the context of~~ it's relationship to health and disease [69–71]. ~~Regarding~~In the field of environmental microbiology, recent reviews have highlighted major developments in the application of ML ~~applied~~ to microbial ecology omics ~~were recently reviewed~~ [72]. This approach has been ~~almost exclusively~~primarily applied to omics experiments ~~containing~~using 16S rRNA gene sequencing data~~, i.e.,~~ which provides taxonomic information on microbial communities~~,~~. ~~instead of shotgun metagenomics which provides both taxonomic and functional information, or to other omics approaches such as metaproteomics and metabolomics.~~ RF-based ML architecture has been ~~the common choice given~~widely used due to its ~~facility~~ease of ~~in~~ implementation, interpretation, low cost, and the requirement of less data~~, when~~ compared

with DL [72]. Nevertheless, other ML algorithms~~ have also been applied in the microbiology field~~, such as~~namely~~ Naïve Bayes (NB), SVM, and KNN, ~~methods~~ have also been applied in the microbiology field [73]. In microbial ecology, the main objective of ML has been to predict the presence of certain microbes (e.g., microbial bioindicators, predicting environmental pollution, and key microbes affecting the performance of biotechnological processes), as well as to predict microbe-microbe ~~interactions~~ and microbe-host interactions~~,~~ and ~~also to support~~facilitate data mining [72,73]. For example, in the particular case of anaerobic digestion microbiology (a biotechnological process in which organic waste is converted to methane by microbial communities), there are ~~a few~~several studies on AI applied to omics data. Three different algorithms, i.e., linear regression, SVM and RF regression, were used to predict the production of medium-chain carboxylates, based on microbial community dynamics (16S rRNA) ~~on~~and bioreactor's productivity data.~~,~~ This study~~and~~ concluded that RF regression was the ~~best~~most effective algorithm for this task [74]. Similarly, another study compared six different ML algorithms, namely GLMNET, RF, NNET, KNN, SVM, and extreme gradient boosting (XGBOOST), to predict the performance of the anaerobic digestion process, using 16S rRNA genomics as the basis for the analysis [75]. Interactions between microorganisms are highly important and influence the activity of microbial communities. Syntrophic interactions among different species are ~~good~~key examples of ~~key~~ microbial interactions, ~~by which~~where microbes exchange electrons either via soluble molecules or directly from cell to cell~~,~~ in an interdependent way. ML was recently used to predict the type of syntrophic interaction that prevails in microbial communities by using a Bayesian network approach [76] ~~using~~. This analysis incorpoerated not only~~, in addition to taxonomic information~~ ~~(~~16S rRNA sequencing~~)~~ but~~,~~ also metagenomics and metatranscriptomics data.

Navigating the frontier: challenges and future horizons in AI innovation

AI in biology research faces several major obstacles that ~~require overcoming~~must be addressed through close collaboration between biologists~~y~~ and computer scien~~c~~tists.~~e~~ ~~researchers~~ Such interdisciplinary collaboerations are essential to exploit the full potential of AI in life sciences [77].

Tackling Technical Challenges in AI-Based Research

A ~~list~~summary of topics that represent challenges in AI-based research ~~are summarised~~is provided in Table 1 (end of the document). For each topic, the description and its~~Besides the description and its~~ connection to ML and/or DL are highlighted.~~,~~ Additionally, ~~each~~the topics are characterised~~is grouped~~ based on seven main technical challenges: (1) noisy datasets; (2) high dimensionality; (3) omics data integration; (4) interpretability; (5) computational requirements; (6) FAIR principles; (7) data size and diversity.

Importantly, data curation and integration across biological subdisciplines ~~remain difficult~~continu~~t~~es to pose significant challe~~a~~nges, requiring the development of new theories and predictive models tailored to biology [77]. A significant problem is the lack of standardised formats across different biological disciplines, which not only complicates ~~both not only~~ the handling of file formats [78] but also makes it difficult to ~~the~~ interpret~~ation of~~ data generated by specialists of each omics data type. Ethical concerns, particularly for animal sciences ~~mostly~~, and privacy issues surrounding data usage need to be addressed, along ~~with~~ ensuring the reliability and safety of AI models through robust validation and transparency. The explainability of AI methods in biological data science ~~is~~remains a significant challenge, as many current approaches lack interpretability.~~,~~ This can~~potentially~~ lead~~ing~~ to decreased trustworthiness and reliability in decision-making processes. Moreover, improving the interpretability of ML-based models in life science~~,~~ ~~improving the interpretability of ML-based models~~ is crucial, as it allows ~~to~~a better understand of the biological mechanisms behind the models.~~,~~ F~~f~~or example, by helping to identify important biomarkers, biological pathways or features that contribute to a specific process [79].

The scarcity of labelled data for training AI models

L~~In recent years, l~~abelling large amounts of data has become one of the main bottleneck in the development of AI systems [80]. ~~In the last~~Over the past fifteen years, advanced ML models, particularly those based on ~~such as the ones based on~~ deep neural networks (DNN), have ~~allowed achieving~~enabled unprecedented results in a variety of fields, including omics research in life sciences [81]. However~~T~~, these models require ~~large~~vast amounts of labeled training ~~labelled~~ data, which in many practical scenarios ~~is~~are either unavailable or very arduous to obtain [82,83]. ~~In fact,~~ Creating hand-labelled training datasets ~~are~~is expensive and time-consuming~~ to create~~, often taking months or years ~~for~~to develop~~ large benchmark sets~~,

~~or~~particularly when domain expertise is required. In response to this technical challenge, a~~n emerging~~ subfield of ML~~,~~ know as ~~called~~ *weakly supervised learning*, a concept developed back in the 1960s, has ~~developed,~~evolved ~~over the last 10 years,~~ into an approach ~~able~~capable of ~~to~~ generating~~e~~ ~~that aims to create~~ large training datasets more rapidly. These datasets, though noisier~~,~~ and of lower-quality~~, but larger-scale training sets~~ are constructed via strategies such as using cheaper annotators, programmatic scripts, or more creative and high-level input from domain experts. In principle, these techniques offer higher-level~~,~~ or ~~otherwise~~ less precise~~,~~ forms of supervision, which, ~~however~~while less accurate, are faster and easier to ~~provide~~obtain than manual annotation [84]. Another approach motivated by the same goal is *semi-supervised learning*, which strives to create large training datasets by combining a small amount of labeled data with a much larger amount of unlabeled data [85]. Omics-based research in life sciences has quickly adopted solutions derived from these approaches ~~in a~~across variousety ~~of~~ applications, such as molecular pathways status prediction in cancer [86] or protein-DNA binding prediction [87], and in the field of plant sciences ~~as well with(~~ applications specific to plant and field phenomics) [88–91]. These ~~works~~examples provide evidence of the effectiveness of *weakly* and *semi-supervised learning* when applied to omics science and ~~they~~ indicate a promising future ~~research~~ direction.

AI for the prediction and annotation of metabolites

Recent ~~research~~developments in AI-based metabolite annotation reflects significant advancements in the applicationeying of ML and DL techniques to improve the accuracy and efficiency of metabolite identification and characterisation in mass spectrometry-based studies [92]. As an example, the chemical language model ‘DeepMet’ utilizes CNNs to learn features from raw MS/MS spectral data and predict human metabolite identities [93]. Similarly, the ‘MetFID’ model uses ANNs to predict molecular fingerprints from MS/MS data, enhancing annotation accuracy compared to existing tools [94]. Computational annotation strategies, including peak grouping, ion adduction analysis, and incorporation of biological knowledge, help overcome the limitations of accurate mass searching alone [95]. ML-based approaches and molecular networking have shown promise in large-scale metabolite annotation, particularly in natural product discovery [96]. Another compelling ML-based tool includes the ‘PeakDecoder’ algorithm which enables metabolite annotation and accurate profiling in multidimensional mass spectrometry measurements [97].

~~However, d~~Despite the availability of ML-based tools for metabolite annotation, inconsistencies in their benchmarking hinder users from selecting the most appropriate method for their research, highlighting the need for standardised evaluation practices [96].

In the context of ecosystem metabolomics, computational methods can now predict previously unobserved metabolites in new microbial communities by leveraging paired metabolome and metagenome data, achieving over 50% accuracy for related metabolites [98]. Additionally, knowledge-based and ML-driven approaches are being developed to refine metabolite identification and analyze primary microbial metabolism in mixed samples [99]. This demonstrates that predictive metabolomics can aid experimental design and reveal valuable insights into numerous community profiles where only metagenomic data is available.

AI-based gene annotation

Advances in genomics have been largely driven by the increasing throughput~~ and, thus,~~ and lower~~ing the~~ cost of DNA sequencing~~ers~~. This~~ which~~ has ~~enabled~~made it possible to sequence thousands of individual genomes within a species and a large number of new species. While generating sequencing data has become a relatively straightforward task, the subsequent processing steps to produce a genome assembly with structural annotations of genomic elements (e.g., genes, promoters, and regulatory elements) and gene functional annotations still represent a challenge. Long-read sequencing technologies have alleviated some of these issues, particularly for genome assembly but the structural annotation of genes, especially in novel genomes, remains problematic in the absence of other extrinsic data sources. Well-known structural annotation tools, such as AUGUSTUS [100], use Hidden-Markov-Models (HMMs) for intrinsic *ab initio* gene finding. A recent *ab initio* gene calling tool, Helixer [101], uses DNNs combined with HMMs to identify genes in ~~all plant genomes~~in genomes without the need for extrinsic data and has shown promising results. Gene functional annotation has traditionally relied on homology to characterise proteins for ascribing a function to newly identified genes. The bottleneck of this methodology is mainly due to knowledge gaps that are producing annotation of genes of ‘unknown function’. DeepGO [102] is a tool which employs DL methods and interactive networks to annotate protein sequences with gene ontology (GO) terms. A later improvement, DeepGOPlus [103] removed many of the restrictions of the earlier version

and no longer needs the interaction networks. DeepGOPlus has the additional advantage of being species agnostic and gives equally good results from protein sequences derived from genomes of newly sequenced species and clades.

FAIR practices for omics data and AI

Despite ~~all~~ the advances ~~already~~outlined~~ described~~ above, challenges in standardising methods and interpreting results persist, highlighting the need for FAIR (Findable, Accessible, Interoperable, Reusable) practices and proper benchmarking to ensure reproducibility and reliability in multi-omics and AI research. ~~As such~~In this context, ontologies ~~are valuable for~~play a crucial role by tagging datasets with metadata~~,~~ thereby ~~enhancing~~improving data understanding and interoperability [104]. They define domain-specific concepts and relationships, making data both human- and machine-readable for easier reuse. However, identifying relevant ontologies can be ~~hard~~difficult due to the large amount available. For example, as of September 2024, 1,147 different ontologies are available in BioPortal [105], including 24 specific for plants and 37 for animal science. ~~Importantly, a~~As ML becomes increasingly indispensable, ensuring data privacy, algorithmic fairness, and transparency will be paramount ~~to~~for maintaining public trust and ensuring equitable access to the benefits of ML-driven advancements [106]. ~~Besides~~Additionally, many open data sources in the life sciences are not yet fully FAIR-compliant, ~~which includes~~with issues ~~with~~related to the absence of~~ the existence of~~ proper metadata, inadequate data documentation, and the lack of ~~-~~crosslinking between datasets. This require significant effort to upgrade their FAIRness for integration into semantic web platforms [107]. While the FAIR principles aim to enhance machine-readability and processing of scientific data, concerns have been raised about potential epistemic losses, such as ~~the~~a reduction in~~of~~ semantic freedom and the displacement of human expertise, ~~hence~~which could discourage~~ing~~ ~~humans~~trust in ~~from trusting~~ AI [108]. To address s~~c~~kepticism and foster trust among stakeholders, a more balanced discussion of both the benefits and epistemic costs of implementing FAIR is needed. Remarkably, a systematic review of 124 LCMS metabolomics software that subsequently retained 61 for detailed analysis based on FAIR Principles for Research Software (FAIR4RS) criteria reported that software fulfilment of these criteria ranged from 21.6% to 71.8%, with no significant improvement in FAIRness over time [109]. Key issues identified included the lack of semantic annotation (0%, *i.e.* no software had semantic annotation

of key information), low registration on Zenodo with DOIs (6.3%), ~~low~~limited containerisation of code or use of virtual machines (14.5%), and insufficiently documented functions in code (16.7%). This recent work ~~thus~~ highlights clear caveats that need to be addressed in further big data-based life science research. To further ~~promote the~~ advanc~~ement of~~e the FAIR principles, collaboration between researchers, data scientists, and data managers is more than ever needed.

Concluding remarks

In conclusion, AI has already transformed biomedical research by accelerating drug discovery, enhancing clinical trials, and providing powerful tools for analysing complex biological data [110]. Its ability to optimize processes, reduce costs, and increase precision is revolutionising how researchers approach ~~medical~~biological challenges. The 2020s is the decade of AI applied to biology: as AI continues to advance, its impact on animal, plant and environmental research will be paramount. AI is reshaping animal research by ~~improving~~enhancing data analysis, ~~enhancing~~improving animal welfare, and reducing ~~the need for~~reliance on traditional testing methods. Through predictive ~~modelling~~modeling, AI helps refine experimental designs, minimising the number of animals used while increasing the accuracy of results. It also ~~aids~~supports the ~~in~~ monitoring of animal behaviour and health, ~~leading~~contributing to better care and more ethical practices. The growing role of AI in animal research will likely lead to more humane, efficient, and scientifically robust studies. ~~Besides~~Additionally, the evolution of ML in plant biology, ranging from its early explorations to its current prominence as a transformative too~~l is a testament to its~~l, demonstrates its remarkable potential. As ML continues to advance~~s~~, its integration with other AI techniques, real-time data processing, and ethical considerations, including agroecological transitions, will shape the future of plant biology research and agricultural practices. In a wider context, AI is making significant strides in environmental research by providing sophisticated tools for monitoring ecosystems, predicting climate patterns, and analysing environmental data. Its ability to process vast amounts of information and identify complex patterns helps in understanding and mitigating the impacts of climate change, pollution, and habitat loss. AI promises to enhance our capacity for environmental stewardship, driving more effective and data-driven strategies to protect and sustain our planet.

ACKNOWLEDGEMENTS

The authors are grateful for financial support from the European Commission's Horizon 2020 Research and Innovation program via the GLOMICAVE (grant agreement no. 952908), MetaboHUB (ANR-11-INBS-0010) and PHENOME (ANR-11-INBS-0012) projects. XD was supported by "La Caixa" Foundation (ID 100010434) via the Junior Leader Fellowship LCF/BQ/PR21/11840001.

COMPETING INTERESTS

The authors declare that they have no competing interests.

REFERENCES

1. Stephens ZD, Lee SY, Faghri F, Campbell RH, Zhai C, Efron MJ, et al.. Big data: Astronomical or genomical? *PLoS Biology*. 2015; doi: 10.1371/journal.pbio.1002195.

2. Giani AM, Gallo GR, Gianfranceschi L, Formenti G. Long walk to genomics: History and current approaches to genome sequencing and assembly. *Computational and Structural Biotechnology Journal*. The Authors; 2020; doi: 10.1016/j.csbj.2019.11.002.

3. Wang Z, Gerstein M, Snyder M. RNA-Seq: a revolutionary tool for transcriptomics. *Nature Reviews Genetics*. 2009; doi: 10.1038/nrg2484.

4. Lowe R, Shirley N, Bleackley M, Dolan S, Shafee T. Transcriptomics technologies. *PLoS Computational Biology*. 2017; doi: 10.1371/journal.pcbi.1005457.

5. Amarasinghe SL, Su S, Dong X, Zappia L, Ritchie ME, Gouil Q. Opportunities and challenges in long-read sequencing data analysis - Genome Biology - Full Text. *Genome Biology*. Genome Biology; 21:1–162020;

6. Marx V. Method of the year: long-read sequencing. *Nature Methods*. Springer US; 2023; doi: 10.1038/s41592-022-01730-w.

7. Griffiths J. A Brief History of Mass Spectrometry. *Analytical Chemistry*. Wiley; 2008; doi: 10.1021/ac8013065.

8. McLafferty FW. A century of progress in molecular mass spectrometry. *Annual Review of Analytical Chemistry*. 2011; doi: 10.1146/annurev-anchem-061010-114018.

9. Mann M, Kelleher NL. Precision proteomics: The case for high resolution and high mass accuracy. *Proceedings of the National Academy of Sciences of the United States of America*. 2008; doi: 10.1073/pnas.0800788105.

10. Alseekh S, Fernie AR. Metabolomics 20 years on: what have we learned and what hurdles remain? *Plant Journal*. 2018; doi: 10.1111/tpj.13950.

11. Hussain S, Mubeen I, Ullah N, Shah SSUD, Khan BA, Zahoor M, et al.. Modern Diagnostic Imaging Technique Applications and Risk Factors in the Medical Field: A Review. *BioMed Research International*. 2022; doi: 10.1155/2022/5164970.

12. Yang W, Feng H, Zhang X, Zhang J, Doonan JH, Batchelor WD, et al.. Crop Phenomics and High-Throughput Phenotyping: Past Decades, Current Challenges, and Future Perspectives. *Molecular Plant*. Elsevier Ltd; 2020; doi: 10.1016/j.molp.2020.01.008.

13. Joyce AR, Palsson B. The model organism as a system: Integrating “omics” data sets. *Nature Reviews Molecular Cell Biology*. 2006; doi: 10.1038/nrm1857.

14. Picard M, Scott-Boyer MP, Bodein A, Périn O, Droit A. Integration strategies of multi-omics data for machine learning analysis. *Computational and Structural Biotechnology Journal*. The Author(s); 2021; doi: 10.1016/j.csbj.2021.06.030.

15. Wang P. On Defining Artificial Intelligence. *Journal of Artificial General Intelligence*. 2019; doi: 10.2478/jagi-2019-0002.

16. Samoili S, López Cobo M, Gómez E, De Prato G, Martínez-Plumed F, Delipetrev B. AI watch: defining Artificial Intelligence : towards an operational definition and taxonomy of artificial intelligence. Luxembourg: Publications Office of the European Union;

17. Kaplan A, Haenlein M. Siri, Siri, in my hand: Who’s the fairest in the land? On the interpretations, illustrations, and implications of artificial intelligence. *Business Horizons*. “Kelley School of Business, Indiana University”; 2019; doi: 10.1016/j.bushor.2018.08.004.

18. Murdoch WJ, Singh C, Kumbier K, Abbasi-Asl R, Yu B. Definitions, methods, and applications in interpretable machine learning. *Proceedings of the National Academy of Sciences of the United States of America*. 2019; doi: 10.1073/pnas.1900654116.

19. Li R, Li L, Xu Y, Yang J. Machine learning meets omics: applications and perspectives. *Briefings in Bioinformatics*. 2021; doi: 10.1093/bib/bbab460.

20. Sohail A, Arif F. Supervised and unsupervised algorithms for bioinformatics and data science. *Prog Biophys Mol Biol*. 2020; doi: 10.1016/j.pbiomolbio.2019.11.012.

21. Domingos P. The master algorithm: how the quest for the ultimate learning machine will remake our world. New York: Basic Books, a member of the Perseus Books Group;

22. van Dijk ADJ, Kootstra G, Kruijer W, de Ridder D. Machine learning in plant science and plant breeding. *iScience*. 2021; doi: 10.1016/j.isci.2020.101890.

23. Schneider A, Hommel G, Blettner M. Linear Regression Analysis. *Deutsches Ärzteblatt international*. 2010; doi: 10.3238/arztebl.2010.0776.

24. Swindel BF. Geometry of Ridge Regression Illustrated. *The American Statistician*. 1981; doi: 10.1080/00031305.1981.10479296.

25. Goodfellow I, Bengio Y, Courville A. Deep learning. Cambridge, Mass: The MIT press;

26. Cortes C, Vapnik V. Support-vector networks. *Mach Learn*. 1995; doi: 10.1007/BF00994018.

27. Mairal J, Koniusz P, Harchaoui Z, Schmid C. Convolutional Kernel Networks. arXiv;

28. Friedman JH. Greedy function approximation: A gradient boosting machine. *Ann Statist*. 2001; doi: 10.1214/aos/1013203451.

29. . Probabilistic Reasoning in Intelligent Systems. Elsevier;

30. Silva JCF, Teixeira RM, Silva FF, Brommonschenkel SH, Fontes EPB. Machine learning approaches and their current application in plant molecular biology: A systematic review. *Plant Science*. Elsevier; 2019; doi: 10.1016/j.plantsci.2019.03.020.

31. Greener JG, Kandathil SM, Moffat L, Jones DT. A guide to machine learning for biologists. *Nature Reviews Molecular Cell Biology*. Springer US; 2021; doi: 10.1038/s41580-021-00407-0.

32. Hinton GE, Osindero S, Teh Y-W. A Fast Learning Algorithm for Deep Belief Nets. *Neural Computation*. 2006; doi: 10.1162/neco.2006.18.7.1527.

33. Lecun Y, Bengio Y, Hinton G. Deep learning. *Nature*. 2015; doi: 10.1038/nature14539.

34. Senior AW, Evans R, Jumper J, Kirkpatrick J, Sifre L, Green T, et al.. Improved protein structure prediction using potentials from deep learning. *Nature*. Springer US; 2020; doi: 10.1038/s41586-019-1923-7.

35. Novakovsky G, Dexter N, Libbrecht MW, Wasserman WW, Mostafavi S. Obtaining genetics insights from deep learning via explainable artificial intelligence. *Nature Reviews Genetics*. Springer US; 2023; doi: 10.1038/s41576-022-00532-2.

36. Mahmud M, Kaiser MS, McGinnity TM, Hussain A. Deep Learning in Mining Biological Data. *Cogn Comput*. 2021; doi: 10.1007/s12559-020-09773-x.

37. Sapoval N, Aghazadeh A, Nute MG, Antunes DA, Balaji A, Baraniuk R, et al.. Current progress and open challenges for applying deep learning across the biosciences. *Nat Commun*. 2022; doi: 10.1038/s41467-022-29268-7.

38. Ching T, Himmelstein DS, Beaulieu-Jones BK, Kalinin AA, Do BT, Way GP, et al.. Opportunities and obstacles for deep learning in biology and medicine. Journal of the Royal Society Interface.

39. Xu C, Jackson SA. Machine learning and complex biological data The revolution of biological techniques and demands for new data mining methods. *Genome Biology*. Genome Biology; 20:1–42019;

40. Adadi A, Berrada M. Peeking Inside the Black-Box: A Survey on Explainable Artificial Intelligence (XAI). *IEEE Access*. IEEE; 2018; doi: 10.1109/ACCESS.2018.2870052.

41. Hajjar G, Barros Santos MC, Bertrand-Michel J, Canlet C, Castelli F, Creusot N, et al.. Scaling-up metabolomics: Current state and perspectives. *TrAC - Trends in Analytical Chemistry*. 2023; doi: 10.1016/j.trac.2023.117225.

42. Boccard J, Rutledge DN. A consensus orthogonal partial least squares discriminant analysis (OPLS-DA) strategy for multiblock Omics data fusion. *Analytica Chimica Acta*. Elsevier B.V.; 2013; doi: 10.1016/j.aca.2013.01.022.

43. Rohart F, Gautier B, Singh A, Lê Cao KA. mixOmics: An R package for 'omics feature selection and multiple data integration. *PLoS Computational Biology*. 2017; doi: 10.1371/journal.pcbi.1005752.

44. Cominetti O, Agarwal S, Oller-Moreno S. Editorial: Advances in methods and tools for multi-omics data analysis. *Frontiers in Molecular Biosciences*. 2023; doi: 10.3389/fmolb.2023.1186822.

45. el Bouhaddani S, Uh HW, Jongbloed G, Hayward C, Klarić L, Kiełbasa SM, et al.. Integrating omics datasets with the OmicsPLS package. *BMC Bioinformatics*. BMC Bioinformatics; 2018; doi: 10.1186/s12859-018-2371-3.

46. Argelaguet R, Velten B, Arnol D, Dietrich S, Zenz T, Marioni JC, et al.. Multi-Omics Factor Analysis—a framework for unsupervised integration of multi-omics data sets. *Molecular Systems Biology*. 2018; doi: 10.15252/msb.20178124.

47. Li W, Zhang Z, Xie B, He Y, He K, Qiu H, et al.. HiOmics: A cloud-based one-stop platform for the comprehensive analysis of large-scale omics data. *Computational and Structural Biotechnology Journal*. Elsevier B.V.; 2024; doi: 10.1016/j.csbj.2024.01.002.

48. Yang W, Feng H, Zhang X, Zhang J, Doonan JH, Batchelor WD, et al.. Crop Phenomics and High-Throughput Phenotyping: Past Decades, Current Challenges, and Future Perspectives. *Molecular Plant*. Elsevier Ltd; 2020; doi: 10.1016/j.molp.2020.01.008.

49. Singh AK, Ganapathysubramanian B, Sarkar S, Singh A. Deep Learning for Plant Stress Phenotyping: Trends and Future Perspectives. *Trends in Plant Science*. Elsevier Ltd; 2018; doi: 10.1016/j.tplants.2018.07.004.

50. Islam S, Reza MN, Samsuzzaman S, Ahmed S, Cho YJ, Noh DH, et al.. Machine vision and artificial intelligence for plant growth stress detection and monitoring: A review. *Precision Agriculture Science and Technology*. 2024; doi: 10.12972/pastj.20240003.

51. Natarajan S, Chakrabarti P, Margala M. Robust diagnosis and meta visualizations of plant diseases through deep neural architecture with explainable AI. *Scientific Reports*. Nature Publishing Group UK; 2024; doi: 10.1038/s41598-024-64601-8.

52. Weihs BJ, Heuschele DJ, Tang Z, York LM, Zhang Z, Xu Z. The State of the Art in Root System Architecture Image Analysis Using Artificial Intelligence: A Review. *Plant Phenomics*. 2024; doi: 10.34133/plantphenomics.0178.

53. Azodi CB, Bolger E, McCarren A, Roantree M, de los Campos G, Shiu SH. Benchmarking parametric and machine learning models for genomic prediction of complex traits. *G3: Genes, Genomes, Genetics*. 2019; doi: 10.1534/g3.119.400498.

54. Rice BR, Lipka AE. Diversifying maize genomic selection models. *Molecular Breeding*. Molecular Breeding; 2021; doi: 10.1007/s11032-021-01221-4.

55. Tong H, Nikoloski Z. Machine learning approaches for crop improvement: Leveraging phenotypic and genotypic big data. *Journal of Plant Physiology*. Elsevier GmbH; 2021; doi: 10.1016/j.jplph.2020.153354.

56. Riedelsheimer C, Czedik-Eysenberg A, Grieder C, Lisec J, Technow F, Sulpice R, et al.. Genomic and metabolic prediction of complex heterotic traits in hybrid maize. *Nature Genetics*. Nature Publishing Group; 2012; doi: 10.1038/ng.1033.

57. Xu S, Xu Y, Gong L, Zhang Q. Metabolomic prediction of yield in hybrid rice. *Plant Journal*. 2016; doi: 10.1111/tpj.13242.

58. Melandri G, Monteverde E, Riewe D, AbdElgawad H, McCouch SR, Bouwmeester H. Can biochemical traits bridge the gap between genomics and plant performance? A study in rice under drought. *Plant Physiology*. 2022; doi: 10.1093/plphys/kiac053.

59. Yan J, Wang X. Machine learning bridges omics sciences and plant breeding. *Trends in Plant Science*. Elsevier Ltd; 2023; doi: 10.1016/j.tplants.2022.08.018.

60. Colantonio V, Ferrão LF V., Tieman DM, Bliznyuk N, Sims C, Klee HJ, et al.. Metabolomic selection for enhanced fruit flavor. *Proceedings of the National Academy of Sciences*. 2022; doi: 10.1073/pnas.2115865119.

61. Dussarrat T, Prigent S, Latorre C, Bernillon S, Flandin A, Díaz FP, et al.. Predictive metabolomics of multiple Atacama plant species unveils a core set of generic metabolites for extreme climate resilience. *New Phytologist*. 2022; doi: 10.1111/nph.18095.

62. Nayeri S, Sargolzaei M, Tulpan D. A review of traditional and machine learning methods applied to animal breeding. *Animal Health Research Reviews*. 2019; doi: 10.1017/S1466252319000148.

63. Chakraborty D, Sharma N, Kour S, Sodhi SS, Gupta MK, Lee SJ, et al.. Applications of Omics Technology for Livestock Selection and Improvement. *Frontiers in Genetics*. 2022; doi: 10.3389/fgene.2022.774113.

64. Wolthuis JC, Magnúsdóttir S, Stigter E, Tang YF, Jans J, Gilbert M, et al.. Multi-country metabolic signature discovery for chicken health classification. *Metabolomics*. Springer US; 2023; doi: 10.1007/s11306-023-01973-4.

65. Chafai N, Hayah I, Houaga I, Badaoui B. A review of machine learning models applied to genomic prediction in animal breeding. *Frontiers in Genetics*. 2023; doi: 10.3389/fgene.2023.1150596.

66. Messad F, Louveau I, Koffi B, Gilbert H, Gondret F. Investigation of muscle transcriptomes using gradient boosting machine learning identifies molecular predictors of feed efficiency in growing pigs. *BMC Genomics*. BMC Genomics; 2019; doi: 10.1186/s12864-019-6010-9.

67. Xue MY, Xie YY, Zhong Y, Ma XJ, Sun HZ, Liu JX. Integrated meta-omics reveals new ruminal microbial features associated with feed efficiency in dairy cattle. *Microbiome*. BioMed Central; 2022; doi: 10.1186/s40168-022-01228-9.

68. Peng Z, Maciel-Guerra A, Baker M, Zhang X, Hu Y, Wang W, et al.. Whole-genome sequencing and gene sharing network analysis powered by machine learning identifies antibiotic resistance sharing between animals, humans and environment in livestock farming. PLoS Computational Biology.

69. Pasolli E, Truong DT, Malik F, Waldron L, Segata N. Machine Learning Meta-analysis of Large Metagenomic Datasets: Tools and Biological Insights. *PLoS Computational Biology*. 2016; doi: 10.1371/journal.pcbi.1004977.

70. Topçuoğlu BD, Lesniak NA, Ruffin MT, Wiens J, Schloss PD. A framework for effective application of machine learning to microbiome-based classification problems. *mBio*. 2020; doi: 10.1128/mBio.00434-20.

71. Krause T, Wassan JT, Mc Kevitt P, Wang H, Zheng H, Hemmje M. Analyzing Large Microbiome Datasets Using Machine Learning and Big Data. *BioMedInformatics*. 2021; doi: 10.3390/biomedinformatics1030010.

72. McElhinney JMWR, Catacutan MK, Mawart A, Hasan A, Dias J. Interfacing Machine Learning and Microbial Omics: A Promising Means to Address Environmental Challenges. *Frontiers in Microbiology*. 2022; doi: 10.3389/fmicb.2022.851450.

73. Qu K, Guo F, Liu X, Lin Y, Zou Q. Application of machine learning in microbiology. *Frontiers in Microbiology*. 2019; doi: 10.3389/fmicb.2019.00827.

74. Liu B, Sträuber H, Saraiva J, Harms H, Silva SG, Kasmanas JC, et al.. Machine learning-assisted identification of bioindicators predicts medium-chain carboxylate production performance of an anaerobic mixed culture. *Microbiome*. BioMed Central; 2022; doi: 10.1186/s40168-021-01219-2.

75. Long F, Wang L, Cai W, Lesnik K, Liu H. Predicting the performance of anaerobic digestion using machine learning algorithms and genomic data. *Water Research*. Elsevier Ltd; 2021; doi: 10.1016/j.watres.2021.117182.

76. Yuan H, Wang X, Lin TY, Kim J, Liu WT. Disentangling the syntrophic electron transfer mechanisms of Candidatus geobacter eutrophica through electrochemical stimulation and

machine learning. *Scientific Reports*. Nature Publishing Group UK; 2021; doi: 10.1038/s41598-021-94628-0.

77. Hassoun S, Jefferson F, Shi X, Stucky B, Wang J, Rosa E. Artificial Intelligence for Biology. *Integrative and Comparative Biology*. 2021; doi: 10.1093/icb/icab188.

78. Thessen AE, Patterson DJ. Data issues in the life sciences. *ZooKeys*. 2011; doi: 10.3897/zookeys.150.1766.

79. Sidak D, Schwarzerová J, Weckwerth W, Waldherr S. Interpretable machine learning methods for predictions in systems biology from omics data. *Frontiers in Molecular Biosciences*. 2022; doi: 10.3389/fmolb.2022.926623.

80. Zhou ZH. A brief introduction to weakly supervised learning. *National Science Review*. 2018; doi: 10.1093/nsr/nwx106.

81. Zhang Z, Zhao Y, Liao X, Shi W, Li K, Zou Q, et al.. Deep learning in omics: A survey and guideline. *Briefings in Functional Genomics*. 2019; doi: 10.1093/bfgp/ely030.

82. Camargo G, Bugatti PH, Saito PTM. Active semi-supervised learning for biological data classification. *PLoS ONE*. 2020; doi: 10.1371/journal.pone.0237428.

83. Huang D, Song B, Wei J, Su J, Coenen F, Meng J. Weakly supervised learning of RNA modifications from low-resolution epitranscriptome data. *Bioinformatics*. 2021; doi: 10.1093/bioinformatics/btab278.

84. Ratner A, De Sa C, Wu S, Selsam D, Ré C. Data programming: Creating large training sets, quickly. *Advances in Neural Information Processing Systems*. :3574–82 2016;

85. van Engelen JE, Hoos HH. A survey on semi-supervised learning. *Machine Learning*. Springer US; 2020; doi: 10.1007/s10994-019-05855-6.

86. Bilal M, Raza SEA, Azam A, Graham S, Ilyas M, Cree IA, et al.. Development and validation of a weakly supervised deep learning framework to predict the status of molecular pathways and key mutations in colorectal cancer from routine histology images: a retrospective study. *The Lancet Digital Health*. The Author(s). Published by Elsevier Ltd. This is an Open Access article under the CC BY-NC-ND 4.0 license; 2021; doi: 10.1016/S2589-7500(21)00180-1.

87. Zhang Q, Zhu L, Bao W, Huang DS. Weakly-Supervised Convolutional Neural Network Architecture for Predicting Protein-DNA Binding. *IEEE/ACM Transactions on Computational Biology and Bioinformatics*. IEEE; 2020; doi: 10.1109/TCBB.2018.2864203.

88. Ghosal S, Zheng B, Chapman SC, Potgieter AB, Jordan DR, Wang X, et al.. A weakly supervised deep learning framework for sorghum head detection and counting. *Plant Phenomics*. AAAS; 2019; doi: 10.34133/2019/1525874.

89. Petti D, Li C. Weakly-supervised learning to automatically count cotton flowers from aerial imagery. *Computers and Electronics in Agriculture*. Elsevier B.V.; 2022; doi: 10.1016/j.compag.2022.106734.

90. Chen J, Deng X, Wen Y, Chen W, Zeb A, Zhang D. Weakly-supervised learning method for the recognition of potato leaf diseases. *Artificial Intelligence Review*. Springer Netherlands; 2023; doi: 10.1007/s10462-022-10374-3.

91. Yan J, Wang X. Unsupervised and semi-supervised learning: the next frontier in machine learning for plant systems biology. *The Plant Journal*. 2022; doi: 10.1111/tpj.15905.

92. Sen P, Lamichhane S, Mathema VB, McGlinchey A, Dickens AM, Khoomrung S, et al.. Deep learning meets metabolomics: A methodological perspective. *Briefings in Bioinformatics*. 2021; doi: 10.1093/bib/bbaa204.

93. Wang F, Liigand J, Tian S, Arndt D, Greiner R, Wishart DS. CFM-ID 4.0: More Accurate ESI-MS/MS Spectral Prediction and Compound Identification. *Analytical Chemistry*. 2021; doi: 10.1021/acs.analchem.1c01465.

94. Fan Z, Alley A, Ghaffari K, Ressom HW. MetFID: artificial neural network-based compound fingerprint prediction for metabolite annotation. *Metabolomics*. Springer US; 2020; doi: 10.1007/s11306-020-01726-7.

95. Domingo-Almenara X, Montenegro-Burke JR, Benton HP, Siuzdak G. Annotation: A Computational Solution for Streamlining Metabolomics Analysis. *Analytical Chemistry*. 2018; doi: 10.1021/acs.analchem.7b03929.

96. de Jonge NF, Mildau K, Meijer D, Louwen JJR, Bueschl C, Huber F, et al.. Good practices and recommendations for using and benchmarking computational metabolomics metabolite annotation tools. *Metabolomics*. Springer US; 2022; doi: 10.1007/s11306-022-01963-y.

97. Bilbao A, Munoz N, Kim J, Orton DJ, Gao Y, Poorey K, et al.. PeakDecoder enables machine learning-based metabolite annotation and accurate profiling in multidimensional mass spectrometry measurements. *Nature Communications*. Springer US; 2023; doi: 10.1038/s41467-023-37031-9.

98. Mallick H, Franzosa EA, Mclver LJ, Banerjee S, Sirota-Madi A, Kostic AD, et al.. Predictive metabolomic profiling of microbial communities using amplicon or metagenomic sequences. *Nat Commun*. Nature Publishing Group; 2019; doi: 10.1038/s41467-019-10927-1.

99. Bartmanski BJ, Rocha M, Zimmermann-Kogadeeva M. Recent advances in data- and knowledge-driven approaches to explore primary microbial metabolism. *Current Opinion in Chemical Biology*. 2023; doi: 10.1016/j.cbpa.2023.102324.

100. Stanke M, Diekhans M, Baertsch R, Haussler D. Using native and syntenically mapped cDNA alignments to improve de novo gene finding. *Bioinformatics*. 2008; doi: 10.1093/bioinformatics/btn013.

101. Holst F, Bolger A, Günther C, Maß J, Triesch S, Kindel F, et al.. Helixer–de novoPrediction of Primary Eukaryotic Gene Models Combining Deep Learning and a Hidden Markov Model. *bioRxiv*. 2023; doi: 10.1101/2023.02.06.527280.

102. Kulmanov M, Khan MA, Hoehndorf R. DeepGO: Predicting protein functions from sequence and interactions using a deep ontology-aware classifier. *Bioinformatics*. 2018; doi: 10.1093/bioinformatics/btx624.

103. Kulmanov M, Hoehndorf R. DeepGOPlus: Improved protein function prediction from sequence. *Bioinformatics*. 2020; doi: 10.1093/bioinformatics/btz595.

104. Dumschott K, Dörpholz H, Laporte MA, Brilhaus D, Schrader A, Usadel B, et al.. Ontologies for increasing the FAIRness of plant research data. *Frontiers in Plant Science*. 2023; doi: 10.3389/fpls.2023.1279694.

105. Whetzel PL, Noy NF, Shah NH, Alexander PR, Nyulas C, Tudorache T, et al.. BioPortal: Enhanced functionality via new Web services from the National Center for Biomedical Ontology to access and use ontologies in software applications. *Nucleic Acids Research*. 2011; doi: 10.1093/nar/gkr469.

106. Gardezi M, Joshi B, Rizzo DM, Ryan M, Prutzer E, Brugler S, et al.. Artificial intelligence in farming: Challenges and opportunities for building trust. *Agronomy Journal*. 2024; doi: 10.1002/agj2.21353.

107. Kamdar MR, Musen MA. An empirical meta-analysis of the life sciences linked open data on the web. *Scientific Data*. Springer US; 2021; doi: 10.1038/s41597-021-00797-y.

108. Chatterjee A, Swierstra T. Making FAIR Trustworthy. *SocArXiv*. 2021; doi: 10.31235/osf.io/x4csm.

109. Du X, Dastmalchi F, Ye H, Garrett TJ, Diller MA, Liu M, et al.. Evaluating LC-HRMS metabolomics data processing software using FAIR principles for research software. *Metabolomics*. 2023; doi: 10.1007/s11306-023-01974-3.

110. Leite ML, de Loiola Costa LS, Cunha VA, Kreniski V, de Oliveira Braga Filho M, da Cunha NB, et al.. Artificial intelligence and the future of life sciences. *Drug Discovery Today*. Elsevier Ltd; 2021; doi: 10.1016/j.drudis.2021.07.002.

841 **Table 1. Major technical challenges in AI-based research**

Technical Challenge	Description	Connection to ML and DL
**1. Noisy Datasets**		
Impact on Model Performance	Noisy or erroneous data can degrade AI model performance, leading to inaccurate predictions, especially in high-precision fields like life sciences.	**ML**: Often struggles with noisy data unless advanced preprocessing is applied. **DL**: Sensitive to noise, impacting performance.
Data Cleaning	Effective noise reduction and robust data cleaning are essential but challenging, particularly at large scales.	**ML**: Requires preprocessing techniques to handle noisy data. **DL**: Needs data cleaning to improve model accuracy.
**2. High Dimensionality**		
Curse of Dimensionality	High-dimensional data can lead to overfitting, making models perform well on training data but poorly on unseen data.	**ML**: Can overfit if dimensionality is not managed; requires feature selection. **DL**: Needs strategies to handle high dimensions.
Feature Selection	Identifying relevant features from a large number of variables is complex and requires advanced techniques to prevent redundancy and enhance model efficiency.	**ML**: Involves sophisticated techniques for effective feature selection. **DL**: Uses embedded feature selection or reduction techniques.
**3. Omics Data Integration**		
Heterogeneity	Omics data from various sources (e.g., genomics, proteomics) are often heterogeneous, differing in scale, format, and noise, complicating integration.	**ML**: Requires methods to handle heterogeneous data. **DL**: Needs effective data fusion strategies for multi-omics.
Data Fusion	Developing methods for effective multi-omics data fusion that preserves biological context and relationships is an ongoing challenge.	**ML**: Must integrate diverse data types. **DL**: Benefits from advanced fusion techniques for comprehensive analysis.
**4. Interpretability of Results**		
Complex Models	Deep learning models, especially those with complex architectures, can act as "black boxes," making it hard to interpret how conclusions are reached.	**ML**: Generally more interpretable than DL but still faces challenges. **DL**: Requires explainability techniques for transparency.
Explainability Techniques	Emerging techniques like SHAP or LIME offer ways to explain AI decisions but may not always provide comprehensive or intuitive insights.	**ML**: May utilize various explainability methods. **DL**: Needs specific techniques for understanding model behavior.

**5. Computational Requirements**		
Resource Intensity	Training state-of-the-art AI models, particularly deep learning models, requires significant computational resources, including high-performance GPUs and extensive memory.	**ML**: Generally less resource-intensive but can still require significant computational power. **DL**: Highly resource-demanding.
Scalability	Ensuring algorithms scale efficiently with increasing data sizes and complexity without excessive computational costs is a critical challenge.	**ML**: Needs to manage scalability efficiently. **DL**: Must handle large-scale data and complex models effectively.
**6. Importance of FAIR Principles**		
Findable, Accessible, Interoperable, Reusable (FAIR)	Adhering to FAIR principles for data and scripts is essential for reproducibility and collaboration but challenging, particularly in standardising metadata and documentation.	**ML**: Requires well-documented datasets for reproducibility. **DL**: Benefits from FAIR practices for consistent data use.
Data Sharing	Facilitating access to well-documented, standardised datasets while maintaining privacy and security can be complex.	**ML**: Needs secure and standardised data-sharing practices. **DL**: Requires access to high-quality, FAIR-compliant datasets.
**7. Data Size and Diversity**		
Scalability of Models	Handling and processing large-scale datasets requires models that can manage and learn from vast amounts of data without compromising performance.	**ML**: Must be scalable to handle large data. **DL**: Efficiently manages large datasets but with high computational costs.
Bias and Generalisation	Ensuring data diversity to avoid biases and ensure models generalize well across different populations or conditions is crucial. Imbalanced datasets can lead to skewed results.	**ML**: Needs diverse data to prevent bias. **DL**: Requires careful data handling to ensure generalisation across conditions.

Manuscript_without_track_changes
Click here to access/download;Manuscript;20250402_AI_review_GigaScience

Artificial Intelligence: the human response to approach the complexity of big data in biology

Giovanni Melandri [1,9,♪], Georges R-Radohery [1,♪], Chloé Beaumont [1], Sara M. de Cripan [7], Coralie Muller [1], Luca Piras [2], Maria Alcina Pereira [4,5], Andreia Ferreira Salvador [4,5], Xavier Domingo-Almenara [7,8], Marie Bolger [6], Sophie Colombié [1,3], Sylvain Prigent [1,3], Biotza Gutierrez Arechederra [2], Nuria Canela Canela [7], Pierre Pétriacq [1,3] *

[1] *Univ. Bordeaux, INRAE, UMR1332 BFP, 33140 Villenave d'Ornon, France*
[2] *EURECAT - Technology Centre of Catalonia, Barcelona, Catalonia, Spain*
[3] *Bordeaux Metabolome, MetaboHUB, PHENOME-EMPHASIS, 33140 Villenave d'Ornon, France*
[4] *Centre of Biological Engineering, University of Minho, 4704-553, Braga, Portugal*
[5] *LABBELS – Associate Laboratory, Braga/Guimarães, Portugal.*
[6] Institute of Bio- and Geosciences, IBG-4: Bioinformatics, Forschungszentrum Jülich, CEPLAS, BioSC, Jülich, Germany
[7] *Centre for Omics Sciences (COS), Eurecat - Technology Centre of Catalonia & Rovira i Virgili University joint unit, Unique Scientific and Technical Infrastructures (ICTS), Reus, Catalonia, Spain*
[8] *Department of Electrical, Electronic and Control Engineering (DEEEA), Universitat Rovira i Virgili, Tarragona, Catalonia, Spain*
[9] *School of Plant Sciences, University of Arizona, Tucson, USA*

[♪] Equal contribution
* Author for correspondence: pierre.petriacq@inrae.fr

ORCID:

Giovanni Melandri	0000-0002-0877-5009
Georges R-Radohery	0000-0003-1405-3106
Maria Alcina Pereira	0000-0002-7110-1779
Andreia Salvador	0000-0001-6037-4248
Xavier Domingo-Almenara	0000-0002-0133-6863
Marie Bolger	0000-0001-6335-1578
Sophie Colombié	0000-0002-9810-4339
Sylvain Prigent	0000-0001-5146-0347
Biotza Gutierrez Arechederra	0000-0001-7411-2580
Núria Canela Canela	0000-0003-0261-2396
Pierre Pétriacq	0000-0001-8151-7420

ABSTRACT

Since the late 2010s, artificial intelligence (AI), encompassing machine learning (ML) and propelled by deep learning (DL), has transformed life science research. It has become a crucial tool for advancing the computational analysis of biological processes, the discovery of natural products, and the study of ecosystem dynamics. This review explores how the rapid increase in high-throughput omics data acquisition has driven the need for AI-based analysis in life sciences, with a particular focus on plant

sciences, animal sciences and microbiology. We highlight the role of omics-based predictive analytics in systems biology and innovative AI-based analytical approaches for gaining deeper insights into complex biological systems. Finally, we discuss the importance of FAIR (findable, accessible, interoperable, reusable) principles for omics data, as well as the future challenges and opportunities presented by the increasing use of AI in life sciences.

Keywords: artificial intelligence, machine learning, deep learning, omics, life science, biology

BACKGROUND

The explosion of omics requires Artificial Intelligence in the study of life sciences

In the past two decades, research and society have entered the 'big data' era of life sciences. Technological advances have enhanced our ability to measure qualitative and quantitative variations of internal biological molecules (e.g., DNA, RNA, proteins, metabolites) and phenotypes, making the acquisition of large and complex omics datasets within a single experiments increasingly common.

The explosion of omics data in life sciences began with genomics which was driven by the emergence of DNA Next-Generation Sequencing (NGS) platforms nearly 20 years ago. While the groundbreaking discovery of the Sanger DNA sequencing method dates back to the 1970s, it took three decades for the advent of second-generation short-read sequencing-based NGS to further revolutionise DNA sequencing, dramatically increasing its affordability and throughput. This has led to the *de novo* assembly of thousands of animal and plant genomes [1,2] and to the discovery of millions of genome-wide single nucleotide polymorphic (SNP) variants . High-throughput analysis of multiple gene transcripts (i.e., transcriptomics) began in the mid-1990s with the introduction of hybridisation-based microarray technologies. However, it was not until the 2000s that NGS enabled a more accurate assessment of the qualitative and quantitative diversity (e.g., large dynamic range of expression levels and alternative splicing variants) of messenger RNAs. This technique, known as RNA sequencing (RNA-seq), uses NGS to sequence complementary DNAs (cDNAs) derived from RNA transcripts[3,4]. The current third-generation single molecule sequencing technologies (e.g., PacBio and Oxford Nanopore Technologies) have further improved the read length, throughput, and accuracy of data collection in the field of genomics and

transcriptomics [5,6]. The field of proteomics and metabolomics relies on the use of mass spectrometry (MS) techniques to explore the diversity of proteins and metabolites in both a qualitative and quantitative manner. Although mass spectrometers have been available since the late 1940s, it was their integration with gas or liquid chromatography (GC and LC) and the development of ionisation techniques such as electrospray ionisation (ESI) and matrix-assisted laser desorption ionisation (MALDI) in the late 1980s that truly expanded their application to biological research [7,8]. There are various ionisation techniques in mass spectrometry and electronic impact ionisation that, while historically important for profiling primary compounds of biological samples, have been largely superseded by softer ionisation methods such as ESI and MALDI. These newer techniques are more suitable for analysing biomolecules as they cause less fragmentation and tend to preserve the integrity of molecules during ionisation. Over the past twenty years, the advancement of high-resolution (HR) MS has been crucial in significantly enhancing the identification of proteins and metabolites. This has driven the widespread application of proteomics and metabolomics in the analysis of complex biological samples [9,10].

Recent advancements in imaging technologies have improved life science research, benefiting not only the medical field[11], but also plant sciences. The field of plant/crop phenomics has rapidly evolved due to breakthroughs in sensor technology, machine vision, and automation technology [12]. Today, automated, non-invasive, high-throughput imaging and sensor technologies generate vast amounts of image and sensor data presenting both opportunities and challenges for analysis.

The ability to generate high-throughput large-scale omics data through advanced technologies offers an unprecedented opportunity for explorating the complexity of biological systems in depth. Furthermore, integrating multiple omics datasets from a single experiment facilitates a ‘holistic’ approach which the potential of revealing how the ‘molecular endophenome’ (at cellular/tissue level) is regulated and connected with the ‘external phenome’ of biological organisms. However, disentangling and deciphering the intricate relationships among tens of thousands (sometimes millions) of molecular variables (i.e., SNPs, transcripts, proteins, and metabolites), which are interconnected among themselves and with the final phenotype, has been a major challenge in biological research over the past two decades [13,14]. The use of high-dimensional solutions on complex omics datasets to address fundamental biological questions exceeds the capacity of the human brain. This requires a computer-based analytical approach, which can

benefit from the constant improvements in machine processing power at all levels (single machine or physical/cloud-based clusters). For these reasons, ‘artificial intelligence’ (AI) has emerged as a key tool in life science research (Fig. 1) with the expectation that AI will lead or assist in most of the future biological discoveries.

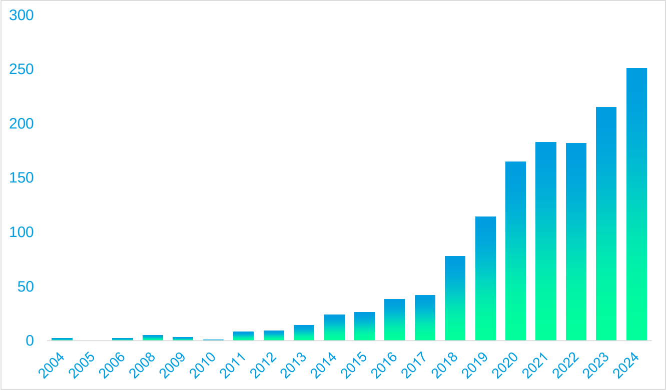

Figure 1. Number of publications found in PubMed including [artificial intelligence] AND [omics] AND [life sciences] from 2004 to 2024. In total, 1362 publications were found (Sept 19th 2024). Considering the last 20 years, a literature search using the queries [omics] AND [artificial intelligence] AND [life sciences] confirms that AI in life sciences is a rapidly expanding field of research.

Artificial Intelligence, Machine Learning and Deep Learning

Despite its widespread use, the term AI remains an elusive ‘buzzword’. From a scientific perspective, the difficulty in defining AI is associated with the complexity of the concept of intelligence *per se* and with the fact that, despite resurgence of interest in AI started in the 1990s, fast progresses in AI research rapidly developed only from the 2010s and, thus, this field of research is far from reaching a level of maturity that can be translated into a clear definition [15].

Oversimplifying, AI can be considered as a branch of computer science focused on programming machines (typically one or more computers) to perform a specific tasks by learning from the information present in specific dataset(s) [16] (Fig. 2). This definition is appropriate only for ‘Artificial Narrow Intelligence’ or ‘Weak AI’, which is currently used for many routine and nearly ubiquitous applications such as spam filtering, speech recognition, language translation, online advertising, image tagging, etc. However, this

definition is not accurate for ‘Artificial General Intelligence’ or ‘Artificial Super Intelligence’ which are both still far from being achieved. These forms of AI aim to develop machines capable of learning and understand from data in ways that are comparable to or surpass human intelligence [17].

Considering ‘Artificial Narrow Intelligence’ (hereafter AI will refer to this term) and, particularly, its most popular subfield ‘Machine Learning’ (ML), the ‘learning’ feature defines the process of using an algorithm which finds complex patterns in the training data and translates them into an object-level algorithm (such as a model of a domain problem) which, in turn, is able to make predictions about unobserved data. It is in the context of ML that biological research has benefited the most from the use of large and complex omics data [18,19]. Biological data-based ML models have the double target of (1) accurately predicting experimental data and (2) using this predicting ability to inform and direct the efforts of future research. When developing ML models, the characteristics of the training data determine the learning approach. . Training data refers to the dataset used to teach an ML model, and a key distinction is whether these training data includes annotations, which determine the learning method applied. Training data can be labeled or unlabeled. Labeled data contains explicit tags, such as categories or numerical values, allowing the model to learn from predefined outcomes. When the data are labeled, the model follows a supervised learning approach. In contrast, unlabeled data lacks predefined tags, requiring the model to extract patterns and relationships independently - a process known as unsupervised learning (Fig. 3) [20]. On the contrary, if the same data are labelled (with qualitative or quantitative tags), the ML model is defined as based on ‘supervised’ learning. Unsupervised ML models are mainly used to deal with clustering problems where the algorithms (e.g., K-means clustering or DBSCAN clustering) find relationships in the overall structure of the training data [20]. In supervised learning the algorithm uses the provided labels as a guide to map data points to specific outcomes or classifications. ~~In contrast, the algorithm independently examine the unlabeled dataset in unsupervised learning and identify patterns, clusters, or relationships without external hints.~~

Machine learning is built on a few fundamental algorithms that serve as the foundation for more advanced techniques [21]. Here, we focus on a non-exhaustive list of these algorithms, particularly those that are interpretable and can clarify the importance of each variable in making predictions. This interpretability is especially valuable in life sciences, where it allows for a thorough utilization of information

found in omics data—such as genomics, proteomics, and metabolomics—to uncover biological insights [22].

First, linear regression, used in supervised learning, predicts a continuous target variable by establishing a linear relationship between inputs and outputs and adjusting parameters to minimise the difference between expected and actual values. Linear regression is highly interpretable, as it establishes a clear linear relationship between input features and the target variable, allowing a straightforward understanding of how changes in each input affect the predicted outcome. The training process involves iterative adjustments to reduce prediction errors, often guided by optimisation techniques [23]. Linear regression forms the basis for methods like Ridge and Lasso regression, which incorporate penalties to mitigate overfitting, where the model performs well on the training data but poorly on new data, by constraining model complexity [24]. These extensions enhance robustness and inform the weight adjustment mechanisms central to neural networks, demonstrating its role as a building block in machine learning [25].

Next, Support Vector Machines (SVMs) address classification by identifying an optimal boundary that maximises the distance to the nearest data points, which are known as support vectors. The support vectors provide insight into which data points are most crucial for the classification boundary. By examining these vectors and their corresponding features, one can infer which aspects of the data are influential in decision-making. For datasets where linear separation is infeasible, SVMs employ kernel functions—such as polynomial or radial basis functions—to transform the data into a higher-dimensional space, enabling complex separations [26]. This emphasis on margin maximisation and spatial transformation influences modern deep learning architectures, notably in convolutional neural networks, where kernel-based operations are prevalent [27].

Decision trees, another supervised learning approach, partition the feature space into distinct regions based on threshold values applied to input variables. Criteria that maximize class separation, such as reducing impurity (e.g., Gini index) or minimizing prediction variance for regression tasks, determine these splits. Their interpretability—from clear, rule-based decisions—makes them particularly appealing for applications requiring transparency, such as omics-driven research (Breiman, Friedman, Olshen, & Stone, 1984). Moreover, integrating them into ensemble methods like random forests, where multiple trees vote to

enhance accuracy, or gradient-boosted trees, which iteratively refine predictions, amplifies their utility. These ensembles illustrate how decision trees evolve into robust predictive tools [28].

We take as the last example Naïve Bayes, which offers a probabilistic framework for classification. It assumes that features are independent within each class and uses probabilities to determine the most likely class for a given set of data. By applying Bayes' theorem, it calculates how likely something belongs to a specific category based on past data. Hence, a Naïve Bayes classifier provides probabilities for each class rather than hard classifications. Furthermore each feature's contribution to the final decision can be calculated based on its likelihood of occurrence on each class thus giving a strong interpretability to the model. However, assumption like data independence could be unrealistic for biological data. Naïve Bayes forms the basis for more advanced probabilistic models, like Bayesian networks. A Bayesian network extends the Naïve Bayes classifier by allowing dependencies between variables, unlike Naïve Bayes, which assumes all features are conditionally independent given the class label. It represents a probabilistic graphical model where nodes (variables) have directed edges (dependencies) between them [29].

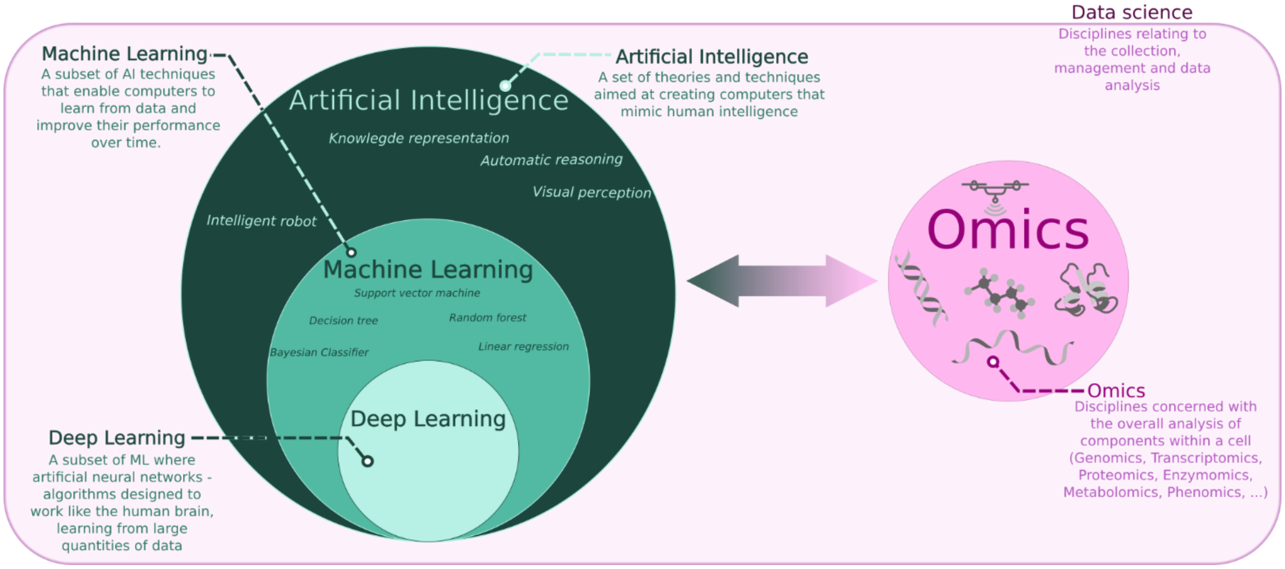

Figure 2. Data science in the era of Artificial Intelligence, Machine Learning and Deep Learning: A Dynamic Schematic Breakdown.

Since its formal introduction in 2006, deep learning (DL) [32], based on diverse artificial neural networks (ANN) algorithms, has further boosted the use of ML in many fields of research, particularly in speech recognition and image analysis [33] but also in the biological field, such as in regulatory genomics

and protein classification [34,35] (Fig. 3). Advanced DL-based models represent the state-of-the-art of prediction accuracy in biological sciences [36,37]. Nevertheless, they require the availability of very large-scale training data (with an associated high computational demand) and their interpretation remains elusive (they are often referred to as 'black-box models'), with this elusiveness representing a limitation in biological experiments involving omics data for which identifying the most important predicting features and feature combinations is of primary importance [38]. Thus, when research is aimed at better understanding the functioning of biological systems, DL-based models are still difficult to be commonly applied [31,39]. It is also for these reasons that in a society where AI algorithms are becoming more central than ever before in all aspects of our daily life, the concepts of 'interpretable ML' and 'explainable AI' are gaining an always increasing attention and importance [35,40].

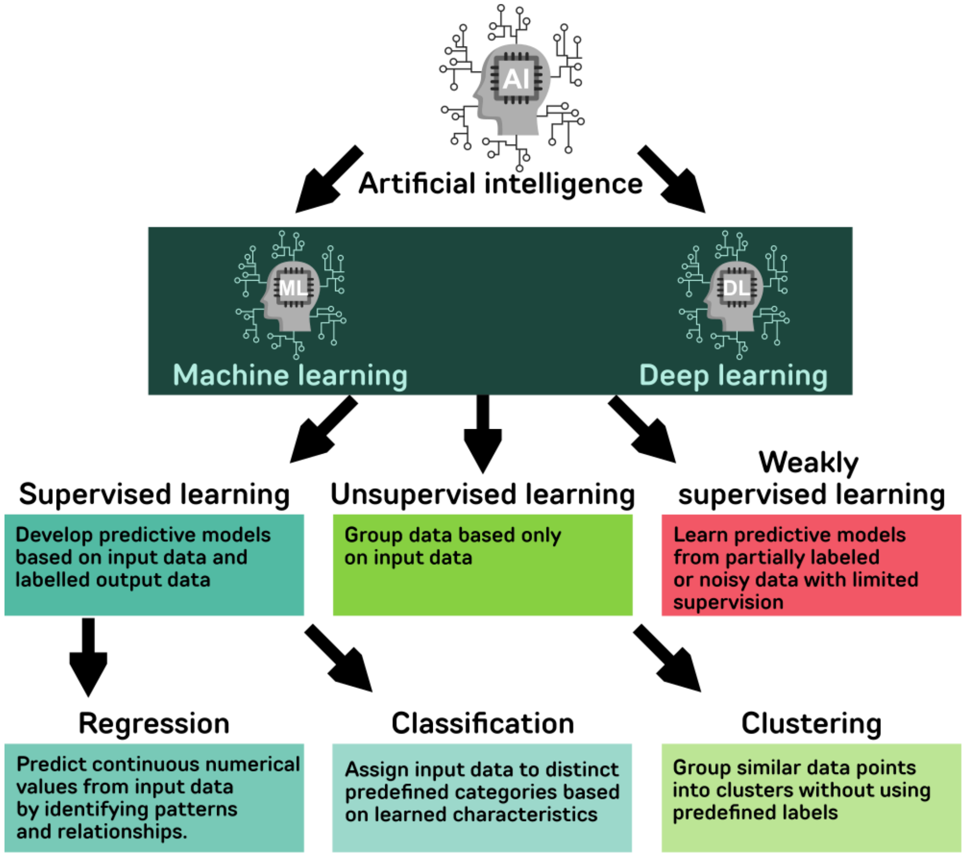

Figure 3. Major approaches in machine learning and deep learning.

Multi-omics integration for ML analysis

As mentioned above, innovations in high-throughput acquisition of different omics data from single experiments are now enabling capturing different layers of biological complexity. In fact, application of omics approaches, such as transcriptomic, proteomics, and metabolomics [41], to large diversity panels and/or sample holds significant promise for unravelling the complexity of living systems. Despite their overall potential for discovery, the diverse nature of omics data acquired by different technological platforms, requires the use of integration strategies to effectively harness their complementary information. Recent advances in multi-omics analysis have been made possible by the development of various tools and methods that can resolve the heterogeneous nature of biological datasets, enabling their effective integration. Notably, consensus orthogonal partial least squares discriminant analysis (OPLS-DA) has emerged as an effective strategy for fusing multi-omics data, combining multiple kernel learning with OPLS-DA [42]. The *mixOmics* R package provides a variety of multivariate methods for integrating omics datasets, including extensions of 'Projection to Latent Structure' models for discriminant analysis and molecular signature identification [43]. Additionally, ML techniques, such as network-based diffusion and DL, are increasingly used to capture complex non-linear associations in multi-omics data [44]. Among the available R resources, packages such *moiraine* (https://plant-food-research-open.github.io/moiraine/) provide a range of integrative methods for multi-omics analyses, including sPLS and DIABLO from the *mixOmics* package [43], sO2PLS from the *OmicsPLS* package [45] and MOFA and MEFISTO from the *MOFA2* package [46].

AI-based analysis of omics data in the fields of Plant Sciences, Animal Sciences, and Microbial Sciences

International initiatives are thriving in the field of AI-based analysis of omics data, aiming to advance the discovery of genotype-phenotype relationships. One such example is the *GLOMICAVE* project (GLobal OMIC data integration on Animal, Vegetal and Environment sectors), an international project that involves all the authors of this review paper. *GLOMICAVE* has created an innovative digital platform that connects genotype to phenotype through Big Data Analytics and AI, utilising extensive public and experimental omic datasets (https://glomicave.eu/). Likewise, cloud-based platforms like HiOmics offer a comprehensive analysis of biomedical large-scale omics data [47]. Such projects aim to facilitate the analysis of primary

data and support large-scale omics experiments, thereby enhancing the utility of omics data on a massive scale and deepening our understanding of entire biological systems. In line with *GLOMICAVE*, and considering that the medical field has been extensively examined from an AI perspective, this review focuses on relevant applications from plant, animal and microbial sciences.

Plant Sciences and AI

The explosion of omics has radically transformed research in plant sciences, simultaneously driving the need for ML to handle datasets characterised by high complexity and dimensionality. A paradigmatic example is plant phenomics, which has rapidly shifted from a promising research sector with the potential of bridging the gap with genomic advances, to becoming a widespread tool in plant and crop sciences [48]. This rapid progress was enabled by integrating advanced sensors and imaging technologies (e.g., RGB, multispectral, hyperspectral, thermal, and fluorescence cameras and sensors) with unmanned aerial vehicles (UAVs or drones) and ground robots, which are able to collect high-throughput phenotyping data. Approaches based on ML algorithms are now a practical and effective strategy for extracting traits and features from massive amounts of imaging- and sensor-based data. DL algorithms (e.g., convolutional neural networks-CNNs) show the highest versatility and success in image-based plant phenotyping. These algorithms are particularly effective in predicting the effects of biotic and abiotic stresses [49,50] and enabling rapid and accurate diagnostics of plant diseases [51]. Additionally, AI applications in root system architecture image analysis are emerging as crucial tools for improving this understudied field of research, which holds significant potential to boost a "Second Green Revolution" in agriculture [52]. Plant breeding is another branch of plant science that has been radically transformed by genomic advances, with breeders increasingly relying on genome-wide SNP markers-based genomic prediction (GP) to accelerate genetic gains for target traits in crops. Classic GP models are based on best linear unbiased prediction (BLUP), but efforts to develop new ML-based and improved GP algorithms are ongoing [53]. Furthermore, different sources of non-genetic variability, and non-additive modes of gene action have made the choice and implementation of GP models challenging for improving complex plant traits, such as biomass and crop yield [54]. One possible solution to this problem is to incorporate other genome-to-phenome intermediate omics data (e.g., transcriptomics, proteomics, metabolomics) into the GP models to enhance their accuracy

and predictive power [55]. The potential of ML models based on single intermediate omics, particularly metabolomics, the omics layer closer to the phenotype, has been demonstrated for the accurate prediction of crop yield, notably in maize [56] and rice [57,58]. However, for plant breeding applications, the integration of large, highly dimensional, and ‘noisy’ omics datasets for complex trait prediction remains a challenging field of study. This challenge will require the use of ML/DL techniques, leveraging their superior capability for big data analytics to effectively handle the complexity and scale of these datasets [59]. Interestingly, recent studies have highlighted innovative approaches in metabolomics-based ML prediction of plant complex traits showing innovative routes to identify breeding targets for plant improvement. For example, Colantonio *et al.* identified candidate metabolites acting as fruit flavor enhancers and suppressors by metabolomics-based ML prediction of tomato and blueberry fruit flavor profiles [60]. In efforts to improve plant tolerance to abiotic stress, Dussarrat *et al.* applied a holistic ML prediction approach on environmental adaptations based on the multi-species metabolome of plants collected in the Atacama desert. This revealed a core set of metabolites targets for extreme climate resilience (sugars, stress-related amino acids, hormones and antioxidants including phenolics and major redox buffers) [61].

Animal Sciences and AI

Modern biotechnologies, bio-sensing hardware, and IT infrastructure have led to a high-throughput data collection era in livestock management, driving the need for faster and more efficient computational methods. While traditional information sources in animal breeding included phenotype and pedigree data, the field is increasingly incorporating genomic data such as SNPs, gene annotations, metabolic pathways, protein interaction networks, gene expression, and protein structure information. These data can enhance trait predictions and improve our understanding of the underlying biological phenotypes [62]. Despite these advancements in animal genetics, many challenges still persis. The widespread adoption of omics technologies is hindered by high cost and the need for expertise across diverse fields. Accurate recording of phenotypic data and population/sample size are other constraints that need to be addressed. However, omics technologies have shown their potential to identify superior and disease-resistant animals at an early stage [63]. For example, the metabolomes of healthy and unhealthy chickens were characterised and compared using untargeted mass spectrometry metabolomics [64]. Researchers were able to accurately

distinguish chicken health status in multiple countries using a random forest based ML model. This approach utilised raw mass spectrometry signals (unannotated m/z values) as input features, effectively overcoming one of the primary limitations of untargeted metabolomics: the need for metabolite annotation and identification. The use of ML models in animal breeding has recently attracted interest due to their exceptional flexibility and ability to capture patterns in large, noisy datasets [65]. For instance, Gradient Tree Boosting (GTB) has proven to be an effective ML algorithm for predicting different breeding values. GTB-based models have identified a subset of genes contributing to feed efficiency in growing pigs using muscle transcriptome data [66]. The potential of combining metagenomics, metatranscriptomics and metabolomics data was evaluated in rumen content, demonstrating their value as predictive markers for feed efficiency and their potential applications for selecting cows with high feed efficiency [67]. By using a RF-based model they were able to predict feed efficiency using a preselected set of metabolites associated with this trait. Antimicrobial-resistant microorganisms pose significant challenges in livestock farming. A recent study [68] evaluated ten supervised learning classifiers to predict *E. coli* strains susceptible or resistant to 26 different antimicrobials using whole genome shotgun sequencing in intensive poultry farming. This findings provided evidence of transmissible drug resistance in food-producing animals, which has contributed to the emergence of drug resistance in zoonotic pathogens.

Microbial Sciences and AI

Microorganisms exist naturally in microbial communities and establish multiple interactions between each other and with their hosts. Omics experiments play a crucial role in studying these microorganisms in their natural environments, eliminating the need for their isolation and cultivation. However, interpreting omics information and integrating results from different studies remains challenging due to the complexity of omics data. AI has been increasingly applied to help interpret the variations found in microbial communities, particularly in the human microbiome and its relationship to health and disease [69–71]. In the field of environmental microbiology, recent reviews have highlighted major developments in the application of ML to microbial ecology omics [72]. This approach has been primarily applied to omics experiments using 16S rRNA gene sequencing data which provides taxonomic information on microbial communities. ~~instead of shotgun metagenomics which provides both taxonomic and functional information, or to other omics~~

approaches such as metaproteomics and metabolomics. RF-based ML architecture has been widely used due to its ease of implementation, interpretation, low cost, and the requirement of less data compared with DL [72]. Nevertheless, other ML algorithms, such as Naïve Bayes (NB), SVM, and KNN, have also been applied in the microbiology field [73]. In microbial ecology, the main objective of ML has been to predict the presence of certain microbes (e.g., microbial bioindicators, predicting environmental pollution, and key microbes affecting the performance of biotechnological processes), as well as to predict microbe-microbe and microbe-host interactions and facilitate data mining [72,73]. For example, in the particular case of anaerobic digestion microbiology (a biotechnological process in which organic waste is converted to methane by microbial communities), there are several studies on AI applied to omics data. Three different algorithms, i.e., linear regression, SVM and RF regression, were used to predict the production of medium-chain carboxylates, based on microbial community dynamics (16S rRNA) and bioreactor's productivity data. This study concluded that RF regression was the most effective algorithm for this task [74]. Similarly, another study compared six different ML algorithms, namely GLMNET, RF, NNET, KNN, SVM, and extreme gradient boosting (XGBOOST), to predict the performance of the anaerobic digestion process, using 16S rRNA genomics as the basis for the analysis [75]. Interactions between microorganisms are highly important and influence the activity of microbial communities. Syntrophic interactions among different species are key examples of microbial interactions, where microbes exchange electrons either via soluble molecules or directly from cell to cell in an interdependent way. ML was recently used to predict the type of syntrophic interaction that prevails in microbial communities by using a Bayesian network approach [76] . This analysis incorporated not only 16S rRNA sequencing but also metagenomics and metatranscriptomics data.

Navigating the frontier: challenges and future horizons in AI innovation

AI in biology research faces several major obstacles that must be addressed through close collaboration between biologists and computer scientists. Such interdisciplinary collaborations are essential to exploit the full potential of AI in life sciences [77].

Tackling Technical Challenges in AI-Based Research

A summary of topics that represent challenges in AI-based research is provided in Table 1 (end of the document). For each topic, the description and its connection to ML and/or DL are highlighted. Additionally, the topics are characterised based on seven main technical challenges: (1) noisy datasets; (2) high dimensionality; (3) omics data integration; (4) interpretability; (5) computational requirements; (6) FAIR principles; (7) data size and diversity.

Importantly, data curation and integration across biological subdisciplines continues to pose significant challenges, requiring the development of new theories and predictive models tailored to biology [77]. A significant problem is the lack of standardised formats across different biological disciplines, which not only complicates the handling of file formats [78] but also makes it difficult to interpret data generated by specialists of each omics data type. Ethical concerns, particularly for animal sciences , and privacy issues surrounding data usage need to be addressed, along ensuring the reliability and safety of AI models through robust validation and transparency. The explainability of AI methods in biological data science remains a significant challenge, as many current approaches lack interpretability. This can lead to decreased trustworthiness and reliability in decision-making processes. Moreover, improving the interpretability of ML-based models in life science is crucial, as it allows a better understand of the biological mechanisms behind the models. For example, by helping to identify important biomarkers, biological pathways or features that contribute to a specific process [79].

The scarcity of labelled data for training AI models

Labelling large amounts of data has become one of the main bottleneck in the development of AI systems [80]. Over the past fifteen years, advanced ML models, particularly those based on deep neural networks (DNN), have enabled unprecedented results in a variety of fields, including omics research in life sciences [81]. However, these models require vast amounts of labeled training data, which in many practical scenarios are either unavailable or very arduous to obtain [82,83]. Creating hand-labelled training datasets is expensive and time-consuming, often taking months or years to develop, particularly when domain expertise is required. In response to this technical challenge, a subfield of ML know as *weakly supervised learning*, a concept developed back in the 1960s, has evolved into an approach capable of generating large training datasets more rapidly. These datasets, though noisier and of lower-quality are constructed via

strategies such as using cheaper annotators, programmatic scripts, or more creative and high-level input from domain experts. In principle, these techniques offer higher-level or less precise forms of supervision, which, while less accurate, are faster and easier to obtain than manual annotation [84]. Another approach motivated by the same goal is *semi-supervised learning*, which strives to create large training datasets by combining a small amount of labeled data with a much larger amount of unlabeled data [85]. Omics-based research in life sciences has quickly adopted solutions derived from these approaches across various applications, such as molecular pathways status prediction in cancer [86] or protein-DNA binding prediction [87], and in the field of plant sciences (applications specific to plant and field phenomics) [88–91]. These examples provide evidence of the effectiveness of *weakly* and *semi-supervised learning* when applied to omics science and indicate a promising future direction.

AI for the prediction and annotation of metabolites

Recent developments in AI-based metabolite annotation reflects significant advancements in the application of ML and DL techniques to improve the accuracy and efficiency of metabolite identification and characterisation in mass spectrometry-based studies [92]. As an example, the chemical language model 'DeepMet' utilizes CNNs to learn features from raw MS/MS spectral data and predict human metabolite identities [93]. Similarly, the 'MetFID' model uses ANNs to predict molecular fingerprints from MS/MS data, enhancing annotation accuracy compared to existing tools [94]. Computational annotation strategies, including peak grouping, ion adduction analysis, and incorporation of biological knowledge, help overcome the limitations of accurate mass searching alone [95]. ML-based approaches and molecular networking have shown promise in large-scale metabolite annotation, particularly in natural product discovery [96]. Another compelling ML-based tool includes the 'PeakDecoder' algorithm which enables metabolite annotation and accurate profiling in multidimensional mass spectrometry measurements [97]. Despite the availability of ML-based tools for metabolite annotation, inconsistencies in their benchmarking hinder users from selecting the most appropriate method for their research, highlighting the need for standardised evaluation practices [96].

In the context of ecosystem metabolomics, computational methods can now predict previously unobserved metabolites in new microbial communities by leveraging paired metabolome and metagenome data,

achieving over 50% accuracy for related metabolites [98]. Additionally, knowledge-based and ML-driven approaches are being developed to refine metabolite identification and analyze primary microbial metabolism in mixed samples [99]. This demonstrates that predictive metabolomics can aid experimental design and reveal valuable insights into numerous community profiles where only metagenomic data is available.

AI-based gene annotation

Advances in genomics have been largely driven by the increasing throughput and lower cost of DNA sequencing. This has made it possible to sequence thousands of individual genomes within a species and a large number of new species. While generating sequencing data has become a relatively straightforward task, the subsequent processing steps to produce a genome assembly with structural annotations of genomic elements (e.g., genes, promoters, and regulatory elements) and gene functional annotations still represent a challenge. Long-read sequencing technologies have alleviated some of these issues, particularly for genome assembly but the structural annotation of genes, especially in novel genomes, remains problematic in the absence of other extrinsic data sources. Well-known structural annotation tools, such as AUGUSTUS [100], use Hidden-Markov-Models (HMMs) for intrinsic *ab initio* gene finding. A recent *ab initio* gene calling tool, Helixer [101], uses DNNs combined with HMMs to identify genes in in genomes without the need for extrinsic data and has shown promising results. Gene functional annotation has traditionally relied on homology to characterise proteins for ascribing a function to newly identified genes. The bottleneck of this methodology is mainly due to knowledge gaps that are producing annotation of genes of ‘unknown function’. DeepGO [102] is a tool which employs DL methods and interactive networks to annotate protein sequences with gene ontology (GO) terms. A later improvement, DeepGOPlus [103] removed many of the restrictions of the earlier version and no longer needs the interaction networks. DeepGOPlus has the additional advantage of being species agnostic and gives equally good results from protein sequences derived from genomes of newly sequenced species and clades.

FAIR practices for omics data and AI

Despite the advances outlined above, challenges in standardising methods and interpreting results persist, highlighting the need for FAIR (Findable, Accessible, Interoperable, Reusable) practices and proper benchmarking to ensure reproducibility and reliability in multi-omics and AI research. In this context, ontologies play a crucial role by tagging datasets with metadata thereby improving data understanding and interoperability [104]. They define domain-specific concepts and relationships, making data both human- and machine-readable for easier reuse. However, identifying relevant ontologies can be difficult due to the large amount available. For example, as of September 2024, 1,147 different ontologies are available in BioPortal [105], including 24 specific for plants and 37 for animal science. As ML becomes increasingly indispensable, ensuring data privacy, algorithmic fairness, and transparency will be paramount for maintaining public trust and ensuring equitable access to the benefits of ML-driven advancements [106]. Additionally, many open data sources in the life sciences are not yet fully FAIR-compliant, with issues related to the absence of proper metadata, inadequate data documentation, and the lack of crosslinking between datasets. This require significant effort to upgrade their FAIRness for integration into semantic web platforms [107]. While the FAIR principles aim to enhance machine-readability and processing of scientific data, concerns have been raised about potential epistemic losses, such as a reduction in semantic freedom and the displacement of human expertise, which could discourage trust in AI [108]. To address skepticism and foster trust among stakeholders, a more balanced discussion of both the benefits and epistemic costs of implementing FAIR is needed. Remarkably, a systematic review of 124 LCMS metabolomics software that subsequently retained 61 for detailed analysis based on FAIR Principles for Research Software (FAIR4RS) criteria reported that software fulfilment of these criteria ranged from 21.6% to 71.8%, with no significant improvement in FAIRness over time [109]. Key issues identified included the lack of semantic annotation (0%, *i.e.* no software had semantic annotation of key information), low registration on Zenodo with DOIs (6.3%), limited containerisation of code or use of virtual machines (14.5%), and insufficiently documented functions in code (16.7%). This recent work highlights clear caveats that need to be addressed in further big data-based life science research. To further advance the FAIR principles, collaboration between researchers, data scientists, and data managers is more than ever needed.

Concluding remarks

In conclusion, AI has already transformed biomedical research by accelerating drug discovery, enhancing clinical trials, and providing powerful tools for analysing complex biological data [110]. Its ability to optimize processes, reduce costs, and increase precision is revolutionising how researchers approach biological challenges. The 2020s is the decade of AI applied to biology: as AI continues to advance, its impact on animal, plant and environmental research will be paramount. AI is reshaping animal research by enhancing data analysis, improving animal welfare, and reducing reliance on traditional testing methods. Through predictive modeling, AI helps refine experimental designs, minimising the number of animals used while increasing the accuracy of results. It also supports the monitoring of animal behaviour and health, contributing to better care and more ethical practices. The growing role of AI in animal research will likely lead to more humane, efficient, and scientifically robust studies. Additionally, the evolution of ML in plant biology, ranging from its early explorations to its current prominence as a transformative tool, demonstrates its remarkable potential. As ML continues to advance, its integration with other AI techniques, real-time data processing, and ethical considerations, including agroecological transitions, will shape the future of plant biology research and agricultural practices. In a wider context, AI is making significant strides in environmental research by providing sophisticated tools for monitoring ecosystems, predicting climate patterns, and analysing environmental data. Its ability to process vast amounts of information and identify complex patterns helps in understanding and mitigating the impacts of climate change, pollution, and habitat loss. AI promises to enhance our capacity for environmental stewardship, driving more effective and data-driven strategies to protect and sustain our planet.

ACKNOWLEDGEMENTS

The authors are grateful for financial support from the European Commission's Horizon 2020 Research and Innovation program via the GLOMICAVE (grant agreement no. 952908), MetaboHUB (ANR-11-INBS-0010) and PHENOME (ANR-11-INBS-0012) projects. XD was supported by "La Caixa" Foundation (ID 100010434) via the Junior Leader Fellowship LCF/BQ/PR21/11840001.

COMPETING INTERESTS

The authors declare that they have no competing interests.

REFERENCES

1. Stephens ZD, Lee SY, Faghri F, Campbell RH, Zhai C, Efron MJ, et al.. Big data: Astronomical or genomical? *PLoS Biology*. 2015; doi: 10.1371/journal.pbio.1002195.

2. Giani AM, Gallo GR, Gianfranceschi L, Formenti G. Long walk to genomics: History and current approaches to genome sequencing and assembly. *Computational and Structural Biotechnology Journal*. The Authors; 2020; doi: 10.1016/j.csbj.2019.11.002.

3. Wang Z, Gerstein M, Snyder M. RNA-Seq: a revolutionary tool for transcriptomics. *Nature Reviews Genetics*. 2009; doi: 10.1038/nrg2484.

4. Lowe R, Shirley N, Bleackley M, Dolan S, Shafee T. Transcriptomics technologies. *PLoS Computational Biology*. 2017; doi: 10.1371/journal.pcbi.1005457.

5. Amarasinghe SL, Su S, Dong X, Zappia L, Ritchie ME, Gouil Q. Opportunities and challenges in long-read sequencing data analysis - Genome Biology - Full Text. *Genome Biology*. Genome Biology; 21:1–162020;

6. Marx V. Method of the year: long-read sequencing. *Nature Methods*. Springer US; 2023; doi: 10.1038/s41592-022-01730-w.

7. Griffiths J. A Brief History of Mass Spectrometry. *Analytical Chemistry*. Wiley; 2008; doi: 10.1021/ac8013065.

8. McLafferty FW. A century of progress in molecular mass spectrometry. *Annual Review of Analytical Chemistry*. 2011; doi: 10.1146/annurev-anchem-061010-114018.

9. Mann M, Kelleher NL. Precision proteomics: The case for high resolution and high mass accuracy. *Proceedings of the National Academy of Sciences of the United States of America*. 2008; doi: 10.1073/pnas.0800788105.

10. Alseekh S, Fernie AR. Metabolomics 20 years on: what have we learned and what hurdles remain? *Plant Journal*. 2018; doi: 10.1111/tpj.13950.

11. Hussain S, Mubeen I, Ullah N, Shah SSUD, Khan BA, Zahoor M, et al.. Modern Diagnostic Imaging Technique Applications and Risk Factors in the Medical Field: A Review. *BioMed Research International*. 2022; doi: 10.1155/2022/5164970.

12. Yang W, Feng H, Zhang X, Zhang J, Doonan JH, Batchelor WD, et al.. Crop Phenomics and High-Throughput Phenotyping: Past Decades, Current Challenges, and Future Perspectives. *Molecular Plant*. Elsevier Ltd; 2020; doi: 10.1016/j.molp.2020.01.008.

13. Joyce AR, Palsson B. The model organism as a system: Integrating "omics" data sets. *Nature Reviews Molecular Cell Biology*. 2006; doi: 10.1038/nrm1857.

14. Picard M, Scott-Boyer MP, Bodein A, Périn O, Droit A. Integration strategies of multi-omics data for machine learning analysis. *Computational and Structural Biotechnology Journal*. The Author(s); 2021; doi: 10.1016/j.csbj.2021.06.030.

15. Wang P. On Defining Artificial Intelligence. *Journal of Artificial General Intelligence*. 2019; doi: 10.2478/jagi-2019-0002.

16. Samoili S, López Cobo M, Gómez E, De Prato G, Martínez-Plumed F, Delipetrev B. AI watch: defining Artificial Intelligence : towards an operational definition and taxonomy of artificial intelligence. Luxembourg: Publications Office of the European Union;

17. Kaplan A, Haenlein M. Siri, Siri, in my hand: Who's the fairest in the land? On the interpretations, illustrations, and implications of artificial intelligence. *Business Horizons*. "Kelley School of Business, Indiana University"; 2019; doi: 10.1016/j.bushor.2018.08.004.

18. Murdoch WJ, Singh C, Kumbier K, Abbasi-Asl R, Yu B. Definitions, methods, and applications in interpretable machine learning. *Proceedings of the National Academy of Sciences of the United States of America*. 2019; doi: 10.1073/pnas.1900654116.

19. Li R, Li L, Xu Y, Yang J. Machine learning meets omics: applications and perspectives. *Briefings in Bioinformatics*. 2021; doi: 10.1093/bib/bbab460.

20. Sohail A, Arif F. Supervised and unsupervised algorithms for bioinformatics and data science. *Prog Biophys Mol Biol*. 2020; doi: 10.1016/j.pbiomolbio.2019.11.012.

21. Domingos P. The master algorithm: how the quest for the ultimate learning machine will remake our world. New York: Basic Books, a member of the Perseus Books Group;

22. van Dijk ADJ, Kootstra G, Kruijer W, de Ridder D. Machine learning in plant science and plant breeding. *iScience*. 2021; doi: 10.1016/j.isci.2020.101890.

23. Schneider A, Hommel G, Blettner M. Linear Regression Analysis. *Deutsches Ärzteblatt international*. 2010; doi: 10.3238/arztebl.2010.0776.

24. Swindel BF. Geometry of Ridge Regression Illustrated. *The American Statistician*. 1981; doi: 10.1080/00031305.1981.10479296.

25. Goodfellow I, Bengio Y, Courville A. Deep learning. Cambridge, Mass: The MIT press;

26. Cortes C, Vapnik V. Support-vector networks. *Mach Learn*. 1995; doi: 10.1007/BF00994018.

27. Mairal J, Koniusz P, Harchaoui Z, Schmid C. Convolutional Kernel Networks. arXiv;

28. Friedman JH. Greedy function approximation: A gradient boosting machine. *Ann Statist*. 2001; doi: 10.1214/aos/1013203451.

29. . Probabilistic Reasoning in Intelligent Systems. Elsevier;

30. Silva JCF, Teixeira RM, Silva FF, Brommonschenkel SH, Fontes EPB. Machine learning approaches and their current application in plant molecular biology: A systematic review. *Plant Science*. Elsevier; 2019; doi: 10.1016/j.plantsci.2019.03.020.

31. Greener JG, Kandathil SM, Moffat L, Jones DT. A guide to machine learning for biologists. *Nature Reviews Molecular Cell Biology*. Springer US; 2021; doi: 10.1038/s41580-021-00407-0.

32. Hinton GE, Osindero S, Teh Y-W. A Fast Learning Algorithm for Deep Belief Nets. *Neural Computation*. 2006; doi: 10.1162/neco.2006.18.7.1527.

33. Lecun Y, Bengio Y, Hinton G. Deep learning. *Nature*. 2015; doi: 10.1038/nature14539.

34. Senior AW, Evans R, Jumper J, Kirkpatrick J, Sifre L, Green T, et al.. Improved protein structure prediction using potentials from deep learning. *Nature*. Springer US; 2020; doi: 10.1038/s41586-019-1923-7.

35. Novakovsky G, Dexter N, Libbrecht MW, Wasserman WW, Mostafavi S. Obtaining genetics insights from deep learning via explainable artificial intelligence. *Nature Reviews Genetics*. Springer US; 2023; doi: 10.1038/s41576-022-00532-2.

36. Mahmud M, Kaiser MS, McGinnity TM, Hussain A. Deep Learning in Mining Biological Data. *Cogn Comput*. 2021; doi: 10.1007/s12559-020-09773-x.

37. Sapoval N, Aghazadeh A, Nute MG, Antunes DA, Balaji A, Baraniuk R, et al.. Current progress and open challenges for applying deep learning across the biosciences. *Nat Commun*. 2022; doi: 10.1038/s41467-022-29268-7.

38. Ching T, Himmelstein DS, Beaulieu-Jones BK, Kalinin AA, Do BT, Way GP, et al.. Opportunities and obstacles for deep learning in biology and medicine. Journal of the Royal Society Interface.

39. Xu C, Jackson SA. Machine learning and complex biological data The revolution of biological techniques and demands for new data mining methods. *Genome Biology*. Genome Biology; 20:1–42019;

40. Adadi A, Berrada M. Peeking Inside the Black-Box: A Survey on Explainable Artificial Intelligence (XAI). *IEEE Access*. IEEE; 2018; doi: 10.1109/ACCESS.2018.2870052.

41. Hajjar G, Barros Santos MC, Bertrand-Michel J, Canlet C, Castelli F, Creusot N, et al.. Scaling-up metabolomics: Current state and perspectives. *TrAC - Trends in Analytical Chemistry*. 2023; doi: 10.1016/j.trac.2023.117225.

42. Boccard J, Rutledge DN. A consensus orthogonal partial least squares discriminant analysis (OPLS-DA) strategy for multiblock Omics data fusion. *Analytica Chimica Acta*. Elsevier B.V.; 2013; doi: 10.1016/j.aca.2013.01.022.

43. Rohart F, Gautier B, Singh A, Lê Cao KA. mixOmics: An R package for ‘omics feature selection and multiple data integration. *PLoS Computational Biology*. 2017; doi: 10.1371/journal.pcbi.1005752.

44. Cominetti O, Agarwal S, Oller-Moreno S. Editorial: Advances in methods and tools for multi-omics data analysis. *Frontiers in Molecular Biosciences*. 2023; doi: 10.3389/fmolb.2023.1186822.

45. el Bouhaddani S, Uh HW, Jongbloed G, Hayward C, Klarić L, Kiełbasa SM, et al.. Integrating omics datasets with the OmicsPLS package. *BMC Bioinformatics*. BMC Bioinformatics; 2018; doi: 10.1186/s12859-018-2371-3.

46. Argelaguet R, Velten B, Arnol D, Dietrich S, Zenz T, Marioni JC, et al.. Multi-Omics Factor Analysis—a framework for unsupervised integration of multi-omics data sets. *Molecular Systems Biology*. 2018; doi: 10.15252/msb.20178124.

47. Li W, Zhang Z, Xie B, He Y, He K, Qiu H, et al.. HiOmics: A cloud-based one-stop platform for the comprehensive analysis of large-scale omics data. *Computational and Structural Biotechnology Journal*. Elsevier B.V.; 2024; doi: 10.1016/j.csbj.2024.01.002.

48. Yang W, Feng H, Zhang X, Zhang J, Doonan JH, Batchelor WD, et al.. Crop Phenomics and High-Throughput Phenotyping: Past Decades, Current Challenges, and Future Perspectives. *Molecular Plant*. Elsevier Ltd; 2020; doi: 10.1016/j.molp.2020.01.008.

49. Singh AK, Ganapathysubramanian B, Sarkar S, Singh A. Deep Learning for Plant Stress Phenotyping: Trends and Future Perspectives. *Trends in Plant Science*. Elsevier Ltd; 2018; doi: 10.1016/j.tplants.2018.07.004.

50. Islam S, Reza MN, Samsuzzaman S, Ahmed S, Cho YJ, Noh DH, et al.. Machine vision and artificial intelligence for plant growth stress detection and monitoring: A review. *Precision Agriculture Science and Technology*. 2024; doi: 10.12972/pastj.20240003.

51. Natarajan S, Chakrabarti P, Margala M. Robust diagnosis and meta visualizations of plant diseases through deep neural architecture with explainable AI. *Scientific Reports*. Nature Publishing Group UK; 2024; doi: 10.1038/s41598-024-64601-8.

52. Weihs BJ, Heuschele DJ, Tang Z, York LM, Zhang Z, Xu Z. The State of the Art in Root System Architecture Image Analysis Using Artificial Intelligence: A Review. *Plant Phenomics*. 2024; doi: 10.34133/plantphenomics.0178.

53. Azodi CB, Bolger E, McCarren A, Roantree M, de los Campos G, Shiu SH. Benchmarking parametric and machine learning models for genomic prediction of complex traits. *G3: Genes, Genomes, Genetics*. 2019; doi: 10.1534/g3.119.400498.

54. Rice BR, Lipka AE. Diversifying maize genomic selection models. *Molecular Breeding*. Molecular Breeding; 2021; doi: 10.1007/s11032-021-01221-4.

55. Tong H, Nikoloski Z. Machine learning approaches for crop improvement: Leveraging phenotypic and genotypic big data. *Journal of Plant Physiology*. Elsevier GmbH; 2021; doi: 10.1016/j.jplph.2020.153354.

56. Riedelsheimer C, Czedik-Eysenberg A, Grieder C, Lisec J, Technow F, Sulpice R, et al.. Genomic and metabolic prediction of complex heterotic traits in hybrid maize. *Nature Genetics*. Nature Publishing Group; 2012; doi: 10.1038/ng.1033.

57. Xu S, Xu Y, Gong L, Zhang Q. Metabolomic prediction of yield in hybrid rice. *Plant Journal*. 2016; doi: 10.1111/tpj.13242.

58. Melandri G, Monteverde E, Riewe D, AbdElgawad H, McCouch SR, Bouwmeester H. Can biochemical traits bridge the gap between genomics and plant performance? A study in rice under drought. *Plant Physiology*. 2022; doi: 10.1093/plphys/kiac053.

59. Yan J, Wang X. Machine learning bridges omics sciences and plant breeding. *Trends in Plant Science*. Elsevier Ltd; 2023; doi: 10.1016/j.tplants.2022.08.018.

60. Colantonio V, Ferrão LF V., Tieman DM, Bliznyuk N, Sims C, Klee HJ, et al.. Metabolomic selection for enhanced fruit flavor. *Proceedings of the National Academy of Sciences*. 2022; doi: 10.1073/pnas.2115865119.

61. Dussarrat T, Prigent S, Latorre C, Bernillon S, Flandin A, Díaz FP, et al.. Predictive metabolomics of multiple Atacama plant species unveils a core set of generic metabolites for extreme climate resilience. *New Phytologist*. 2022; doi: 10.1111/nph.18095.

62. Nayeri S, Sargolzaei M, Tulpan D. A review of traditional and machine learning methods applied to animal breeding. *Animal Health Research Reviews*. 2019; doi: 10.1017/S1466252319000148.

63. Chakraborty D, Sharma N, Kour S, Sodhi SS, Gupta MK, Lee SJ, et al.. Applications of Omics Technology for Livestock Selection and Improvement. *Frontiers in Genetics*. 2022; doi: 10.3389/fgene.2022.774113.

64. Wolthuis JC, Magnúsdóttir S, Stigter E, Tang YF, Jans J, Gilbert M, et al.. Multi-country metabolic signature discovery for chicken health classification. *Metabolomics*. Springer US; 2023; doi: 10.1007/s11306-023-01973-4.

65. Chafai N, Hayah I, Houaga I, Badaoui B. A review of machine learning models applied to genomic prediction in animal breeding. *Frontiers in Genetics*. 2023; doi: 10.3389/fgene.2023.1150596.

66. Messad F, Louveau I, Koffi B, Gilbert H, Gondret F. Investigation of muscle transcriptomes using gradient boosting machine learning identifies molecular predictors of feed efficiency in growing pigs. *BMC Genomics*. BMC Genomics; 2019; doi: 10.1186/s12864-019-6010-9.

67. Xue MY, Xie YY, Zhong Y, Ma XJ, Sun HZ, Liu JX. Integrated meta-omics reveals new ruminal microbial features associated with feed efficiency in dairy cattle. *Microbiome*. BioMed Central; 2022; doi: 10.1186/s40168-022-01228-9.

68. Peng Z, Maciel-Guerra A, Baker M, Zhang X, Hu Y, Wang W, et al.. Whole-genome sequencing and gene sharing network analysis powered by machine learning identifies antibiotic resistance sharing between animals, humans and environment in livestock farming. PLoS Computational Biology.

69. Pasolli E, Truong DT, Malik F, Waldron L, Segata N. Machine Learning Meta-analysis of Large Metagenomic Datasets: Tools and Biological Insights. *PLoS Computational Biology*. 2016; doi: 10.1371/journal.pcbi.1004977.

70. Topçuoğlu BD, Lesniak NA, Ruffin MT, Wiens J, Schloss PD. A framework for effective application of machine learning to microbiome-based classification problems. *mBio*. 2020; doi: 10.1128/mBio.00434-20.

71. Krause T, Wassan JT, Mc Kevitt P, Wang H, Zheng H, Hemmje M. Analyzing Large Microbiome Datasets Using Machine Learning and Big Data. *BioMedInformatics*. 2021; doi: 10.3390/biomedinformatics1030010.

72. McElhinney JMWR, Catacutan MK, Mawart A, Hasan A, Dias J. Interfacing Machine Learning and Microbial Omics: A Promising Means to Address Environmental Challenges. *Frontiers in Microbiology*. 2022; doi: 10.3389/fmicb.2022.851450.

73. Qu K, Guo F, Liu X, Lin Y, Zou Q. Application of machine learning in microbiology. *Frontiers in Microbiology*. 2019; doi: 10.3389/fmicb.2019.00827.

74. Liu B, Sträuber H, Saraiva J, Harms H, Silva SG, Kasmanas JC, et al.. Machine learning-assisted identification of bioindicators predicts medium-chain carboxylate production performance of an anaerobic mixed culture. *Microbiome*. BioMed Central; 2022; doi: 10.1186/s40168-021-01219-2.

75. Long F, Wang L, Cai W, Lesnik K, Liu H. Predicting the performance of anaerobic digestion using machine learning algorithms and genomic data. *Water Research*. Elsevier Ltd; 2021; doi: 10.1016/j.watres.2021.117182.

76. Yuan H, Wang X, Lin TY, Kim J, Liu WT. Disentangling the syntrophic electron transfer mechanisms of Candidatus geobacter eutrophica through electrochemical stimulation and machine learning. *Scientific Reports*. Nature Publishing Group UK; 2021; doi: 10.1038/s41598-021-94628-0.

77. Hassoun S, Jefferson F, Shi X, Stucky B, Wang J, Rosa E. Artificial Intelligence for Biology. *Integrative and Comparative Biology*. 2021; doi: 10.1093/icb/icab188.

78. Thessen AE, Patterson DJ. Data issues in the life sciences. *ZooKeys*. 2011; doi: 10.3897/zookeys.150.1766.

79. Sidak D, Schwarzerová J, Weckwerth W, Waldherr S. Interpretable machine learning methods for predictions in systems biology from omics data. *Frontiers in Molecular Biosciences*. 2022; doi: 10.3389/fmolb.2022.926623.

80. Zhou ZH. A brief introduction to weakly supervised learning. *National Science Review*. 2018; doi: 10.1093/nsr/nwx106.

81. Zhang Z, Zhao Y, Liao X, Shi W, Li K, Zou Q, et al.. Deep learning in omics: A survey and guideline. *Briefings in Functional Genomics*. 2019; doi: 10.1093/bfgp/ely030.

82. Camargo G, Bugatti PH, Saito PTM. Active semi-supervised learning for biological data classification. *PLoS ONE*. 2020; doi: 10.1371/journal.pone.0237428.

83. Huang D, Song B, Wei J, Su J, Coenen F, Meng J. Weakly supervised learning of RNA modifications from low-resolution epitranscriptome data. *Bioinformatics*. 2021; doi: 10.1093/bioinformatics/btab278.

84. Ratner A, De Sa C, Wu S, Selsam D, Ré C. Data programming: Creating large training sets, quickly. *Advances in Neural Information Processing Systems*. :3574–82 2016;

85. van Engelen JE, Hoos HH. A survey on semi-supervised learning. *Machine Learning*. Springer US; 2020; doi: 10.1007/s10994-019-05855-6.

86. Bilal M, Raza SEA, Azam A, Graham S, Ilyas M, Cree IA, et al.. Development and validation of a weakly supervised deep learning framework to predict the status of molecular pathways and key mutations in colorectal cancer from routine histology images: a retrospective study. *The Lancet Digital Health*. The Author(s). Published by Elsevier Ltd. This is an Open Access article under the CC BY-NC-ND 4.0 license; 2021; doi: 10.1016/S2589-7500(21)00180-1.

87. Zhang Q, Zhu L, Bao W, Huang DS. Weakly-Supervised Convolutional Neural Network Architecture for Predicting Protein-DNA Binding. *IEEE/ACM Transactions on Computational Biology and Bioinformatics*. IEEE; 2020; doi: 10.1109/TCBB.2018.2864203.

88. Ghosal S, Zheng B, Chapman SC, Potgieter AB, Jordan DR, Wang X, et al.. A weakly supervised deep learning framework for sorghum head detection and counting. *Plant Phenomics*. AAAS; 2019; doi: 10.34133/2019/1525874.

89. Petti D, Li C. Weakly-supervised learning to automatically count cotton flowers from aerial imagery. *Computers and Electronics in Agriculture*. Elsevier B.V.; 2022; doi: 10.1016/j.compag.2022.106734.

90. Chen J, Deng X, Wen Y, Chen W, Zeb A, Zhang D. Weakly-supervised learning method for the recognition of potato leaf diseases. *Artificial Intelligence Review*. Springer Netherlands; 2023; doi: 10.1007/s10462-022-10374-3.

91. Yan J, Wang X. Unsupervised and semi-supervised learning: the next frontier in machine learning for plant systems biology. *The Plant Journal*. 2022; doi: 10.1111/tpj.15905.

92. Sen P, Lamichhane S, Mathema VB, McGlinchey A, Dickens AM, Khoomrung S, et al.. Deep learning meets metabolomics: A methodological perspective. *Briefings in Bioinformatics*. 2021; doi: 10.1093/bib/bbaa204.

93. Wang F, Liigand J, Tian S, Arndt D, Greiner R, Wishart DS. CFM-ID 4.0: More Accurate ESI-MS/MS Spectral Prediction and Compound Identification. *Analytical Chemistry*. 2021; doi: 10.1021/acs.analchem.1c01465.

94. Fan Z, Alley A, Ghaffari K, Ressom HW. MetFID: artificial neural network-based compound fingerprint prediction for metabolite annotation. *Metabolomics*. Springer US; 2020; doi: 10.1007/s11306-020-01726-7.

95. Domingo-Almenara X, Montenegro-Burke JR, Benton HP, Siuzdak G. Annotation: A Computational Solution for Streamlining Metabolomics Analysis. *Analytical Chemistry*. 2018; doi: 10.1021/acs.analchem.7b03929.

96. de Jonge NF, Mildau K, Meijer D, Louwen JJR, Bueschl C, Huber F, et al.. Good practices and recommendations for using and benchmarking computational metabolomics metabolite annotation tools. *Metabolomics*. Springer US; 2022; doi: 10.1007/s11306-022-01963-y.

97. Bilbao A, Munoz N, Kim J, Orton DJ, Gao Y, Poorey K, et al.. PeakDecoder enables machine learning-based metabolite annotation and accurate profiling in multidimensional mass spectrometry measurements. *Nature Communications*. Springer US; 2023; doi: 10.1038/s41467-023-37031-9.

98. Mallick H, Franzosa EA, Mclver LJ, Banerjee S, Sirota-Madi A, Kostic AD, et al.. Predictive metabolomic profiling of microbial communities using amplicon or metagenomic sequences. *Nat Commun*. Nature Publishing Group; 2019; doi: 10.1038/s41467-019-10927-1.

99. Bartmanski BJ, Rocha M, Zimmermann-Kogadeeva M. Recent advances in data- and knowledge-driven approaches to explore primary microbial metabolism. *Current Opinion in Chemical Biology*. 2023; doi: 10.1016/j.cbpa.2023.102324.

100. Stanke M, Diekhans M, Baertsch R, Haussler D. Using native and syntenically mapped cDNA alignments to improve de novo gene finding. *Bioinformatics*. 2008; doi: 10.1093/bioinformatics/btn013.

101. Holst F, Bolger A, Günther C, Maß J, Triesch S, Kindel F, et al.. Helixer–de novoPrediction of Primary Eukaryotic Gene Models Combining Deep Learning and a Hidden Markov Model. *bioRxiv*. 2023; doi: 10.1101/2023.02.06.527280.

102. Kulmanov M, Khan MA, Hoehndorf R. DeepGO: Predicting protein functions from sequence and interactions using a deep ontology-aware classifier. *Bioinformatics*. 2018; doi: 10.1093/bioinformatics/btx624.

103. Kulmanov M, Hoehndorf R. DeepGOPlus: Improved protein function prediction from sequence. *Bioinformatics*. 2020; doi: 10.1093/bioinformatics/btz595.

104. Dumschott K, Dörpholz H, Laporte MA, Brilhaus D, Schrader A, Usadel B, et al.. Ontologies for increasing the FAIRness of plant research data. *Frontiers in Plant Science*. 2023; doi: 10.3389/fpls.2023.1279694.

105. Whetzel PL, Noy NF, Shah NH, Alexander PR, Nyulas C, Tudorache T, et al.. BioPortal: Enhanced functionality via new Web services from the National Center for Biomedical Ontology to access and use ontologies in software applications. *Nucleic Acids Research*. 2011; doi: 10.1093/nar/gkr469.

106. Gardezi M, Joshi B, Rizzo DM, Ryan M, Prutzer E, Brugler S, et al.. Artificial intelligence in farming: Challenges and opportunities for building trust. *Agronomy Journal*. 2024; doi: 10.1002/agj2.21353.

107. Kamdar MR, Musen MA. An empirical meta-analysis of the life sciences linked open data on the web. *Scientific Data*. Springer US; 2021; doi: 10.1038/s41597-021-00797-y.

108. Chatterjee A, Swierstra T. Making FAIR Trustworthy. *SocArXiv*. 2021; doi: 10.31235/osf.io/x4csm.

109. Du X, Dastmalchi F, Ye H, Garrett TJ, Diller MA, Liu M, et al.. Evaluating LC-HRMS metabolomics data processing software using FAIR principles for research software. *Metabolomics*. 2023; doi: 10.1007/s11306-023-01974-3.

110. Leite ML, de Loiola Costa LS, Cunha VA, Kreniski V, de Oliveira Braga Filho M, da Cunha NB, et al.. Artificial intelligence and the future of life sciences. *Drug Discovery Today*. Elsevier Ltd; 2021; doi: 10.1016/j.drudis.2021.07.002.

Table 1. Major technical challenges in AI-based research

Technical Challenge	Description	Connection to ML and DL
**1. Noisy Datasets**		
Impact on Model Performance	Noisy or erroneous data can degrade AI model performance, leading to inaccurate predictions, especially in high-precision fields like life sciences.	**ML**: Often struggles with noisy data unless advanced preprocessing is applied. **DL**: Sensitive to noise, impacting performance.
Data Cleaning	Effective noise reduction and robust data cleaning are essential but challenging, particularly at large scales.	**ML**: Requires preprocessing techniques to handle noisy data. **DL**: Needs data cleaning to improve model accuracy.
**2. High Dimensionality**		
Curse of Dimensionality	High-dimensional data can lead to overfitting, making models perform well on training data but poorly on unseen data.	**ML**: Can overfit if dimensionality is not managed; requires feature selection. **DL**: Needs strategies to handle high dimensions.
Feature Selection	Identifying relevant features from a large number of variables is complex and requires advanced techniques to prevent redundancy and enhance model efficiency.	**ML**: Involves sophisticated techniques for effective feature selection. **DL**: Uses embedded feature selection or reduction techniques.
**3. Omics Data Integration**		
Heterogeneity	Omics data from various sources (e.g., genomics, proteomics) are often heterogeneous, differing in scale, format, and noise, complicating integration.	**ML**: Requires methods to handle heterogeneous data. **DL**: Needs effective data fusion strategies for multi-omics.
Data Fusion	Developing methods for effective multi-omics data fusion that preserves biological context and relationships is an ongoing challenge.	**ML**: Must integrate diverse data types. **DL**: Benefits from advanced fusion techniques for comprehensive analysis.
**4. Interpretability of Results**		
Complex Models	Deep learning models, especially those with complex architectures, can act as "black boxes," making it hard to interpret how conclusions are reached.	**ML**: Generally more interpretable than DL but still faces challenges. **DL**: Requires explainability techniques for transparency.
Explainability Techniques	Emerging techniques like SHAP or LIME offer ways to explain AI decisions but may not always provide comprehensive or intuitive insights.	**ML**: May utilize various explainability methods. **DL**: Needs specific techniques for understanding model behavior.

**5. Computational Requirements**		
Resource Intensity	Training state-of-the-art AI models, particularly deep learning models, requires significant computational resources, including high-performance GPUs and extensive memory.	**ML**: Generally less resource-intensive but can still require significant computational power. **DL**: Highly resource-demanding.
Scalability	Ensuring algorithms scale efficiently with increasing data sizes and complexity without excessive computational costs is a critical challenge.	**ML**: Needs to manage scalability efficiently. **DL**: Must handle large-scale data and complex models effectively.
**6. Importance of FAIR Principles**		
Findable, Accessible, Interoperable, Reusable (FAIR)	Adhering to FAIR principles for data and scripts is essential for reproducibility and collaboration but challenging, particularly in standardising metadata and documentation.	**ML**: Requires well-documented datasets for reproducibility. **DL**: Benefits from FAIR practices for consistent data use.
Data Sharing	Facilitating access to well-documented, standardised datasets while maintaining privacy and security can be complex.	**ML**: Needs secure and standardised data-sharing practices. **DL**: Requires access to high-quality, FAIR-compliant datasets.
**7. Data Size and Diversity**		
Scalability of Models	Handling and processing large-scale datasets requires models that can manage and learn from vast amounts of data without compromising performance.	**ML**: Must be scalable to handle large data. **DL**: Efficiently manages large datasets but with high computational costs.
Bias and Generalisation	Ensuring data diversity to avoid biases and ensure models generalize well across different populations or conditions is crucial. Imbalanced datasets can lead to skewed results.	**ML**: Needs diverse data to prevent bias. **DL**: Requires careful data handling to ensure generalisation across conditions.

Table 1. Major technical challenges in AI-basec

Technical Challenge	Description
**1. Noisy Datasets**	
Impact on Model Performance	Noisy or erroneous data can degrade AI model performance, leading to inaccurate predictions, especially in high-precision fields like life sciences.
Data Cleaning	Effective noise reduction and robust data cleaning are essential but challenging, particularly at large scales.
**2. High Dimensionality**	
Curse of Dimensionality	High-dimensional data can lead to overfitting, making models perform well on training data but poorly on unseen data.
Feature Selection	Identifying relevant features from a large number of variables is complex and requires advanced techniques to prevent redundancy and enhance model efficiency.
**3. Omics Data Integration**	
Heterogeneity	Omics data from various sources (e.g., genomics, proteomics) are often heterogeneous, differing in scale, format, and noise, complicating integration.
Data Fusion	Developing methods for effective multi-omics data fusion that preserves biological context and relationships is an ongoing challenge.
**4. Interpretability of Results**	
Complex Models	Deep learning models, especially those with complex architectures, can act as "black boxes," making it hard to interpret how conclusions are reached.
Explainability Techniques	Emerging techniques like SHAP or LIME offer ways to explain AI decisions but may not always provide comprehensive or intuitive insights.
**5. Computational Requirements**	

Resource Intensity	Training state-of-the-art AI models, particularly deep learning models, requires significant computational resources, including high-performance GPUs and extensive memory.
Scalability	Ensuring algorithms scale efficiently with increasing data sizes and complexity without excessive computational costs is a critical challenge.
**6. Importance of FAIR Principles**	
Findable, Accessible, Interoperable, Reusable (FAIR)	Adhering to FAIR principles for data and scripts is essential for reproducibility and collaboration but challenging, particularly in standardising metadata and documentation.
Data Sharing	Facilitating access to well-documented, standardised datasets while maintaining privacy and security can be complex.
**7. Data Size and Diversity**	
Scalability of Models	Handling and processing large-scale datasets requires models that can manage and learn from vast amounts of data without compromising performance.
Bias and Generalisation	Ensuring data diversity to avoid biases and ensure models generalize well across different populations or conditions is crucial. Imbalanced datasets can lead to skewed results.

d research

Connection to ML and DL
**ML**: Often struggles with noisy data unless advanced preprocessing is applied. **DL**: Sensitive to noise, impacting performance.
**ML**: Requires preprocessing techniques to handle noisy data. **DL** Needs data cleaning to improve model accuracy.
**ML**: Can overfit if dimensionality is not managed; requires feature selection. **DL**: Needs strategies to handle high dimensions.
**ML**: Involves sophisticated techniques for effective feature selection. **DL**: Uses embedded feature selection or reduction techniques.
**ML**: Requires methods to handle heterogeneous data. **DL**: Needs effective data fusion strategies for multi-omics.
**ML**: Must integrate diverse data types. **DL**: Benefits from advanced fusion techniques for comprehensive analysis.
**ML**: Generally more interpretable than DL but still faces challenges. **DL**: Requires explainability techniques for transparency.
**ML**: May utilize various explainability methods. **DL**: Needs specific techniques for understanding model behavior.

ML: Generally less resource-intensive but can still require significant computational power. **DL** Highly resource-demanding.

ML: Needs to manage scalability efficiently. **DL**: Must handle large-scale data and complex models effectively.

ML: Requires well-documented datasets for reproducibility. **DL** Benefits from FAIR practices for consistent data use.

ML: Needs secure and standardised data-sharing practices. **DL**: Requires access to high-quality, FAIR-compliant datasets.

ML: Must be scalable to handle large data. **DL**: Efficiently manages large datasets but with high computational costs.

ML: Needs diverse data to prevent bias. **DL**: Requires careful data handling to ensure generalisation across conditions.

Figure 1

Click here to access/download;Figure;Figure1.xlsx

Search query: ((artificial intelligence) AND (omics)) AND (life sciences)

Year	Count
2004	2
2005	0
2006	2
2008	5
2009	3
2010	1
2011	8
2012	9
2013	14
2014	24
2015	26
2016	38
2017	42
2018	78
2019	114
2020	165
2021	183
2022	182
2023	215
2024	251

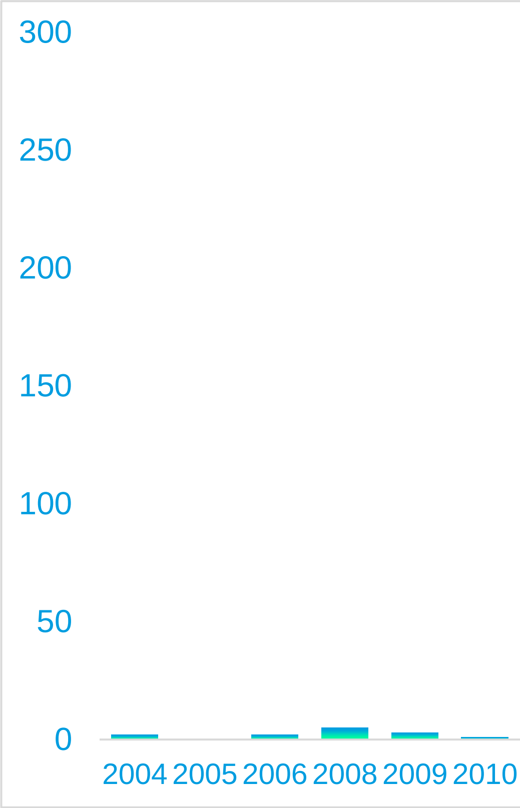

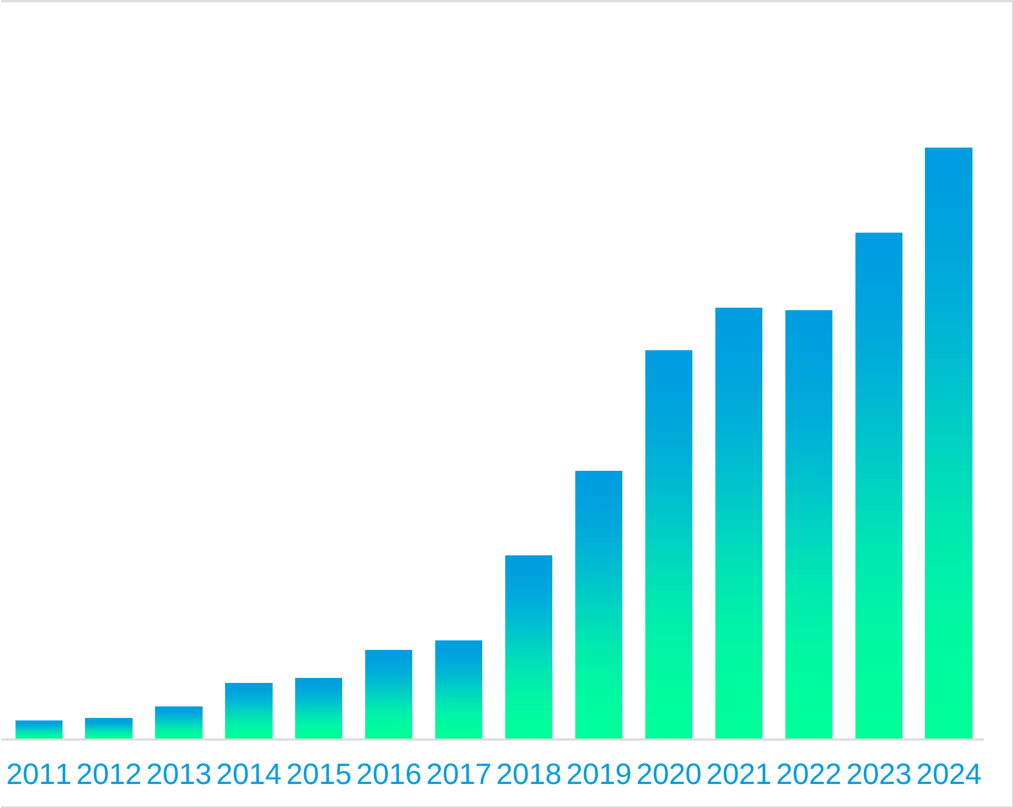
2011
2012
2013
2014
2015
2016
2017
2018
2019
2020
2021
2022
2023
2024

Figure 2

Click here to access/download;Figure;Fig2_review_V2.2.png

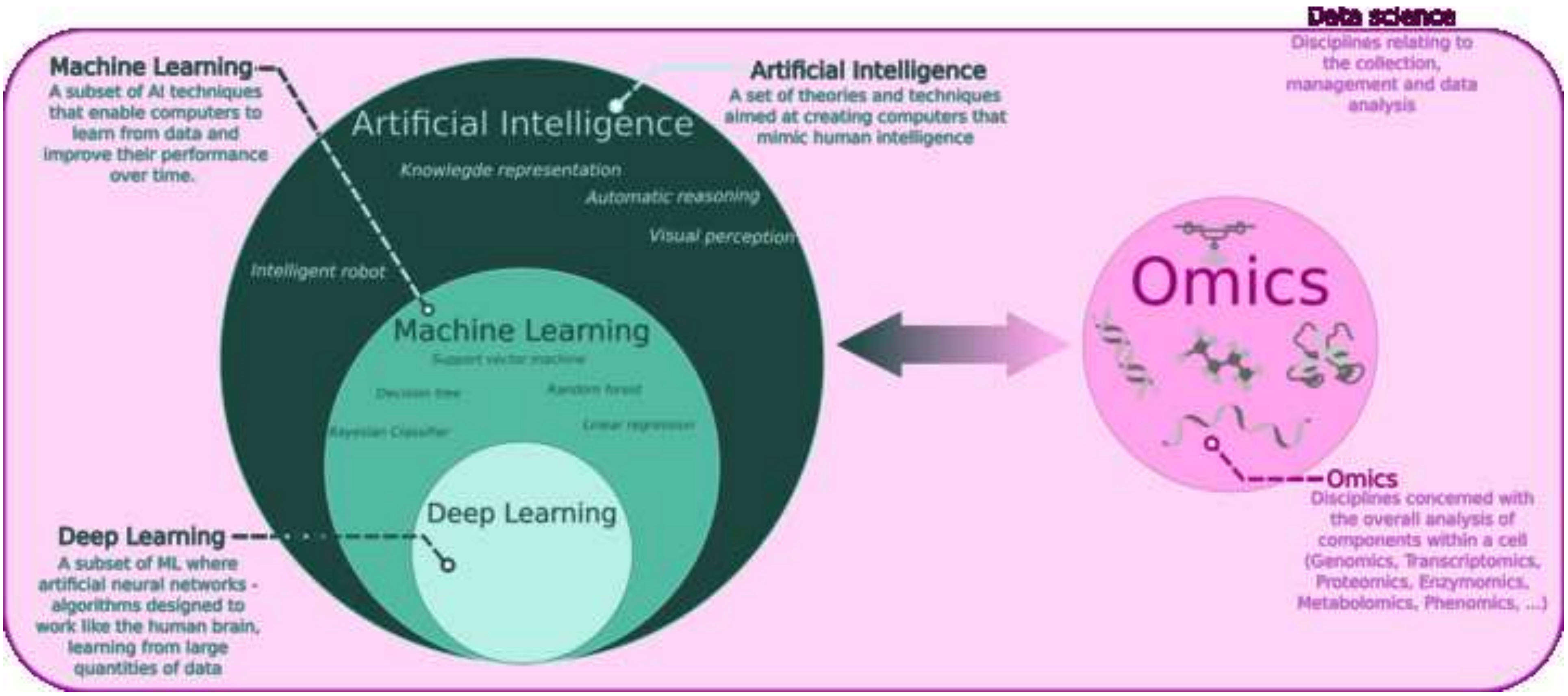

Figure 3

Click here to access/download;Figure;figure3_13022025.png

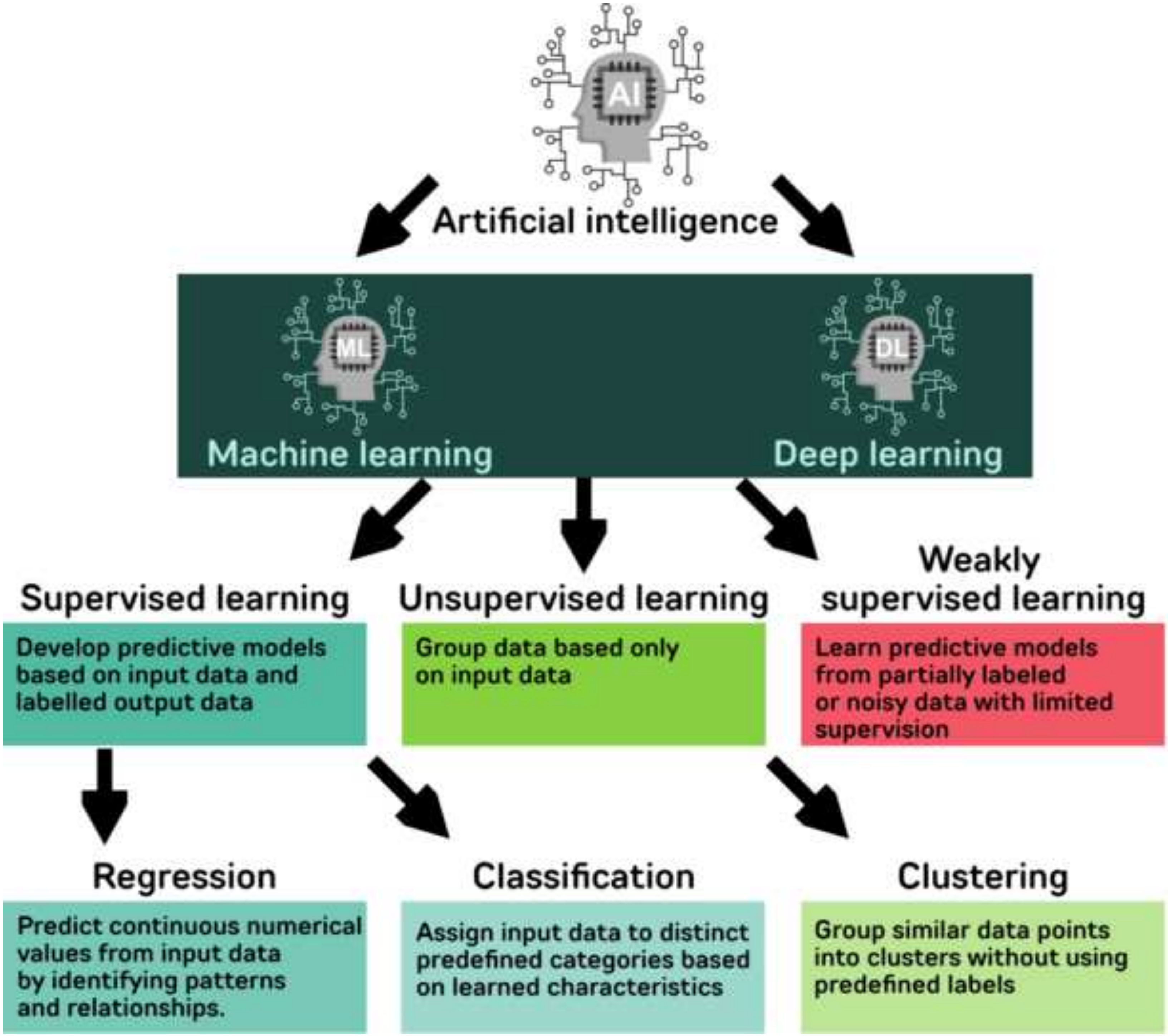

Supplement: giaf057_GIGA-D-24-00489_Revision_1 [file giaf057_giga-d-24-00489_revision_1.pdf]
