# Supplementary material for: Artificial intelligence: the human response to approach the complexity of big data in biology
Source: Gigascience. 2025 Jun 12;14:giaf057. doi: 10.1093/gigascience/giaf057 (PMC12160488; doi:10.1093/gigascience/giaf057)
Supplement: giaf057_Authors_Response_To_Reviewer_Comments_original_submission [file giaf057_authors_response_to_reviewer_comments_original_submission.pdf]

Author's Response To Reviewer Comments

Dear Editor,
Dear Reviewers,

Thank you for your insightful comments, which have significantly contributed to the enhancement of our manuscript. We have carefully addressed all the reviewers' comments. Additionally, the manuscript has been proofread by a native English speaker to ensure consistency in language quality throughout. We have also verified the cited references to correct any duplication errors that may have been present in the previous version. The revision includes the manuscript with track changes, and a clean version without track changes.

Sincerely,
Dr Pierre Pétriacq, on behalf of all the authors

p5 Several algorithms from ML are listed with little explanation and few references.
- We have revised this section of the manuscript, explaining in more detail the algorithms mentioned and providing additional references (L149-L209 of the revised manuscript).

p6 Diagram. Why is Omics fully inside deep learning?
- Thanks for this valuable verification. We have revised Figure 2, with AI and omics being connected concepts of Data sciences.

p6 L154 "Advanced DL-based models represent the state of the art" - can you provide a few example references to justify this?
- We have provided two references on the role of deep learning algorithms as the most advanced for modelling in biological sciences (L225 of the revised manuscript).

P7 text section. This needs a proof read, some of the wording is off.
- We revised this text section to improve its readability (L237-256 of the revised manuscript).

P7 Figure 3. The arrows pointing to the bottom row don't make much sense - e.g. Weakly supervised learning doesn't point anywhere?
- Thank you for the comment. We have included a revised figure 3 that should address this comment.

p8 I believe GLOMICAVE is the work of the authors? Therefore, the review should be clear about this and why it is likely mentioned.
- We have explained that GLOMICAVE is a project involving all the authors of the review article (L262-263 of the revised manuscript).

p9 Fig 4. I don't really see the value in this figure as it stands?
- We thank you for this valuable verification. We also agree that this figure lacks relevance and have therefore removed it from the revised manuscript.

p9 L210 "The only feasible strategy" - I don't agree with this statement
- We have changed it into “a practical and effective strategy” (L284-285 of the revised manuscript).

P268 "Microbial Ecology Sciences" - the title of section seems over specific given the broadness of some of the others?
- We have changed the title of the section to “Microbial Sciences and AI” to make it less specific (L351 of the revised manuscript).

p14 L329 Weak supervision is not an emerging field. It has been around for a long time.
- We have removed the term 'emerging' from the description of weakly supervised learning and explained that it is an old concept that has been fully exploited in ML over the last 10 years (L423-426 of the revised manuscript).

p17 L407 I assume 0% is a typo
- We have double-checked the publication and 0% is real, not a typo (https://link.springer.com/article/10.1007/s11306-023-01974-3). For improved clarity, we have precised L507: "0%, i.e. no software had semantic annotation of key information".

em Editorial Manager®

Cookie Preference Center

We use cookies which are necessary to make our site work. We may also use additional cookies to analyze, improve and personalize our content and your digital experience. For more information, see our Cookie Policy.

You may choose not to allow some types of cookies. However, blocking some types may impact your experience of our site and the services we are able to offer. See the different category headings below to find out more or change your settings.

Allow all

Manage Consent Preferences

Strictly Necessary Cookies

Always active

These cookies are necessary for the website to function and cannot be switched off in our systems. They are usually only set in response to actions made by you which amount to a request for services, such as setting your privacy preferences, logging in or filling in forms. You can set your browser to block or alert you about these cookies, but some parts of the site will not then work. These cookies do not store any personally identifiable information.

Functional Cookies

These cookies enable the website to provide enhanced functionality and personalisation. They may be set by us or by third party providers whose services we have added to our pages. If you do not allow these cookies then some or all of these services may not function properly.

Performance Cookies

These cookies allow us to count visits and traffic sources so we can measure and improve the performance of our site. They help us to know which pages are the most and least popular and see how visitors move around the site.

Targeting Cookies

These cookies may be set through our site by our advertising partners. They may be used by those companies to build a profile of your interests and show you relevant adverts on other sites. If you do not allow these cookies, you will experience less targeted advertising.

Cookie List

Clear

Apply Cancel

Consent Leg.Interest

Confirm my choices

Powered by onetrust
