# Supplementary material for: Artificial intelligence: the human response to approach the complexity of big data in biology
Source: Gigascience. 2025 Jun 12;14:giaf057. doi: 10.1093/gigascience/giaf057 (PMC12160488; doi:10.1093/gigascience/giaf057)
Supplement: giaf057_Reviewer_2_Report_Original_submission — Andrew French -- 2/10/2025 [file giaf057_reviewer_2_report_original_submission.pdf]

Reviewer Report

Title: Artificial Intelligence: the human response to approach the complexity of big data in biology

Version: Original Submission **Date:** 2/10/2025

Reviewer name: Andrew French

Reviewer Comments to Author:

This is potentially a timely review of an important area. The review, whilst short, covers a broad range of topics. To note, despite being interdisciplinary myself, I am approaching the review with a more computational AI background.There are however a number of areas of the review which I don't have a high confidence in, unfortunately. As noted below, I have some concerns with the quality of some figures, and the interpretation of some of the text. Importantly, I don't understand why there are ~200 references listed in the bibliography, but only ~100 cited. Apologies if I am missing a supplemental section? But in the main PDF shared from the journal, only 99 references are cited.I share these points in the hope they are useful in evolving the manuscript in the future:p5 Several algorithms from ML are listed with little explanation and few references.p6 Diagram. Why is Omics fully inside deep learning?p6L154 "Advanced DL-based models represent the state of the art" - can you provide a few example references to justify this?P7 text section. This needs a proof read, some of the wording is off.P7Figure 3. The arrows pointing to the bottom row don't make much sense - e.g. Weakly supervised learning doesn't point anywhere?p8 I believe GLOMICAVE is the work of the authors? Therefore, the review should be clear about this and why it is likely mentioned.p9Fig 4. I don't really see the value in this figure as it stands?p9L210 "The only feasible strategy" - I don't agree with this statementP268 "Microbial Ecology Sciences" - the title of section seems over specific given the broadness of some of the others?p14L329 Weak supervision is not an emerging field. It has been around for a long time.p17 L407 I assume 0% is a typoI would recommend a proof read to fix a few wording issues throughout.

Level of Interest

Please indicate how interesting you found the manuscript: Choose an item.

Quality of Written English

Please indicate the quality of language in the manuscript: Choose an item.

Declaration of Competing Interests

Please complete a declaration of competing interests, considering the following questions:

- Have you in the past five years received reimbursements, fees, funding, or salary from an organisation that may in any way gain or lose financially from the publication of this manuscript, either now or in the future?

- Do you hold any stocks or shares in an organisation that may in any way gain or lose financially from the publication of this manuscript, either now or in the future?
- Do you hold or are you currently applying for any patents relating to the content of the manuscript?
- Have you received reimbursements, fees, funding, or salary from an organization that holds or has applied for patents relating to the content of the manuscript?
- Do you have any other financial competing interests?
- Do you have any non-financial competing interests in relation to this paper?

If you can answer no to all of the above, write 'I declare that I have no competing interests' below. If your reply is yes to any, please give details below.

I declare that I have no competing interests

I agree to the open peer review policy of the journal. I understand that my name will be included on my report to the authors and, if the manuscript is accepted for publication, my named report including any attachments I upload will be posted on the website along with the authors' responses. I agree for my report to be made available under an Open Access Creative Commons CC-BY license (http://creativecommons.org/licenses/by/4.0/). I understand that any comments which I do not wish to be included in my named report can be included as confidential comments to the editors, which will not be published.

Choose an item.

To further support our reviewers, we have joined with Publons, where you can gain additional credit to further highlight your hard work (see: https://publons.com/journal/530/gigascience). On publication of this paper, your review will be automatically added to Publons, you can then choose whether or not to claim your Publons credit. I understand this statement.

Yes Choose an item.
